# Economic valuation of ecosystem services in agriculture and agroforestry: A meta-analysis

**DOI:** 10.1371/journal.pone.0360792

**Published:** 2026-10-16

**Authors:** Mahugnon Maxime Obe, Cokou Patrice Kpadé, Alphonse Singbo, Terence Epule Epule, Vincent Poirier

**Affiliations:** 1 Agriculture and Agri-Food Research Institute, Université du Québec en Abitibi-Témiscamingue, Notre-Dame-du-Nord, Québec, Canada; 2 Department of Agri-Food Economics and Consumer Science, University Laval, Québec, Quebec, Canada; Southern Westphalia University of Applied Sciences - Soest Campus: Fachhochschule Sudwestfalen - Standort Soest, GERMANY

## Abstract

The economic valuation of ecosystem services in agriculture and agroforestry is crucial for sustainable development. However, valuation methods vary significantly across contexts. Using the PRISMA framework, we systematically reviewed 146 studies, combined effect sizes through meta-analytic modeling, and conducted spatial autocorrelation analyses to explore geographic patterns. Market price valuation (MPV) and contingent valuation (CVM) are the most used methods in ecosystem service assessments, accounting for 25.8% and 20.8% of studies, respectively. The CVM and life cycle impact assessment (LCIA) approaches play a key role in interdisciplinary research, connecting all ecosystem service categories. Analysis revealed extreme heterogeneity and significant publication bias for cultural (CULT), provisioning (PROV), and regulating (REG) services. Valuation outcomes are strongly influenced by the choice of methods (such as MPV and benefit transfer methods) and unit standardization practices, with effect sizes decreasing when values are standardized to United States dollars per ha per year (USD/ha/year). Spatial autocorrelation was minimal, indicating the importance of local context in the valuation results. Temporal trends indicate episodic changes in the relative effectiveness of valuation approaches. Empirically, MPV showed greater robustness (18%) than CVM, which exhibited greater sensitivity to data constraints (16%). These findings highlight the need to prioritize data quality and context specificity within standardized valuation frameworks to improve policy relevance and comparability in ecosystem service assessments, particularly for underrepresented CULT and supporting (SUP) services.

## Introduction

According to Lehmann et al. [1] and Achmad et al. [2], agroforestry is a sustainable land-use system based on the deliberate integration of trees or other perennial woody plants with crops and/or livestock on the same land unit. It encompasses several forms of integration, including silvoarable systems, agrisilviculture, silvopastoralism, agrisilvopastoralism, silvofisheries, forest beekeeping, sericulture, and multipurpose tree production systems. This system aims to increase overall land productivity while generating economic, ecological, social, and cultural benefits. Conventional agriculture, also referred to as industrial or modern agriculture, is an agricultural production system characterized by the intensive use of external inputs, technologies, and management practices designed to maximize yields and efficiency. It typically relies on monoculture, synthetic inputs, intensive mechanization, large-scale irrigation, production specialization, genetic selection, and intensive livestock production. In contrast, agroforestry is generally regarded as an agroecological approach that can improve the sustainability of agricultural systems, including through broader integration into both organic and conventional farming systems [3].

Diverse agricultural and agroforestry systems provide essential ecosystem services, defined as the environmental and economic benefits people derive from natural ecosystems, and these services should be considered when evaluating production systems because they underpin agricultural productivity [1]. These are typically classified as PROV (such as food and water), REG (such as climate and disease control), SUP (such as nutrient cycling and soil formation), and CULT (such as recreation and spiritual values) [4]. However, differences in stakeholder values and priorities for ecosystem use and management often lead to disputes over resource allocation [5].

Valuing ecosystem services is crucial for sustainable development because it underscores the economic importance of nature and its contributions to human well-being, especially when expressed through monetary valuation methods that convert complex ecological functions into economic indicators that are easily understood by policymakers, businesses, and land managers [6–10]. These approaches connect ecological integrity with socio-economic goals by enabling cost-benefit analyses (CBA), market-based instruments such as payments for ecosystem services, and resource allocation strategies, thereby supporting conservation efforts and encouraging sustainable agricultural and agroforestry practices [7,8].

Ecosystem services must be valued using methods appropriate to data availability, service characteristics, and valuation objectives. These include direct market valuation (for services traded in markets), indirect techniques such as travel cost and hedonic pricing, stated preference methods such as contingent valuation and choice modelling, and benefit transfer approaches that adapt estimates from existing studies to new contexts [6,8,11–19]. Nonetheless, these methods face ongoing challenges, such as inconsistent terminology and classifications, difficulties in capturing plural and intangible values (like cultural or existence benefits), overlaps among ecosystem service categories and valuation methods, and uncertainties in scaling results from local to broader levels. These issues hinder comparability, reproducibility, and effective integration into policy and management, highlighting the need for comprehensive assessments and critical syntheses to guide best practices [6,12,13,20].

Valuing biodiversity and ecosystem services is crucial for informed decision-making. Yet current approaches face issues such as methodological inconsistencies, inaccurate assessments of non-market and CULT services, and unresolved trade-offs between PROV and REG services. These challenges hinder effective policy development and sustainable land management [21–30]. This study systematically reviews and synthesizes the principal approaches used to estimate the economic value of ecosystem services in agricultural and agroforestry systems. Our objective is to identify the most frequently applied methods, assess their regional and contextual applicability, and evaluate their respective strengths and limitations within agroecosystem contexts. We further posit that substantial heterogeneity and potential publication bias characterize these valuations, owing in part to methodological variation, contextual differences, and inconsistencies in unit standardization.

### Conceptual framework and critical perspectives on economic valuation

Economic valuation of ecosystem services seeks to quantify nature’s value to society, but it captures that value only imperfectly [31,32]. Such value also includes intangible aesthetic, cultural, spiritual, and ethical dimensions that reflect obligations to nature, biodiversity, and future generations, and that cannot always be reduced to monetary terms [13,33–35], and need complementary approaches [36,37].

The utilitarian approach [6,38,39] primarily monetizes ecosystem services [40,41] by emphasizing their usefulness to human socio-economic systems [30,42,43]. However, this framing does not fully capture intrinsic ecological values [44,45], cultural dimensions [46,47], or critical ecosystem thresholds [48,49]. Neoclassical economics extends this logic by assigning monetary estimates to non-use values, making such valuation a central strand of environmental economics [50]. Methods such as contingent valuation and discrete choice experiments infer these values from stated preferences; because they rely on willingness to pay, they remain anthropocentric [38,39,51]. As a result, environmental policy is often structured around CBA reasoning, reinforcing the idea that value is expressed through preferences and utilities within market-based economies [38,52]. At the same time, following Costanza [53] and Groot et al. [54], monetary valuation should be understood as a way of expressing trade-offs in currency units, not as equivalent to market price, privatization, or commodification (S1 Fig).

The value of natural capital [3,19,20] is often undervalued when ecosystems are treated as abundant or insufficiently constrained, despite the complexity of ecosystem services and the need to assess them across multiple value systems [55–58]. Values ranging from economic and social to aesthetic and intrinsic are not always well represented in policy processes, particularly when decision frameworks privilege metrics that are easier to quantify [59,60]. While use values derive from direct human benefits, non-use values [61] including moral and aesthetic significance, are less readily monetized and may be better addressed through non-monetary approaches. Consequently, a plural valuation framework is needed: biophysical and mapping methods assess ecological conditions and functions [62–64], whereas participatory and sociocultural approaches are better suited to eliciting sociocultural values and perspectives on intrinsic value [39]. Costanza [53] and Baveye et al. [34], further argue that intrinsic value is categorically distinct: if understood as a rights-based claim independent of human experience, it cannot be captured through willingness-to-pay methods and does not yet have a widely established assessment framework.

Even when intended to support environmental protection, Gómez‐Baggethun and Ruiz-Pérez [38], argue that monetary valuation can facilitate commodification both discursively and technically. In their account, applying exchange logic to ecosystems makes marketization more likely under prevailing institutional conditions, framing human–nature relations in terms of scarcity and profit. By contrast, Costanza [53] maintains that valuation and commodification are conceptually distinct and should remain institutionally separate. From this perspective, valuation can highlight the public importance of ecosystems without justifying privatization. The central issue, therefore, is not whether valuation should be abandoned, but under what institutional conditions its results are used. Because many ecosystem services have public-good characteristics, monetary valuation does not in itself imply treatment as private market commodities [54]. The disagreement is thus better understood as contextual rather than absolute: the risk of commodification depends on the governance settings through which valuation is translated into policy or markets [38,65] (S1 Fig).

Baveye et al. [34] similarly note that cultural and spiritual values, including identity and heritage, are difficult to monetize and therefore often remain outside economic assessments [46]. Monetization should therefore not be treated as the default approach, particularly where it obscures important non-monetary functions and services [34]. They further argue that monetary valuation becomes increasingly limited as ecosystem services are more complex, large-scale, interconnected, and subtle [66]. The literature accordingly points to complementary approaches, including deliberative monetary valuation [44,67], socio-cultural valuation [47], biophysical indicators [48], shared and social value frameworks [68], and integrative frameworks such as the Life Framework of Values [45], that are generally proposed as complements to, rather than substitutes for, economic valuation. In this vein, Turner et al. [6] advocate a sequential decision-support system embedded in broader multi-criteria assessment and deliberation; Costanza [69] integrating valuation around three sub-goals, efficient allocation, fair distribution, and sustainable scale, within a whole-systems perspective; and Chan et al. [46] argue that effective ecosystem-service decision-making requires distinguishing among multiple value dimensions and addressing non-material values through diverse social-science methods. Accordingly, economic valuation is best treated as one input into decision-making rather than as a standalone criterion [38,54]. Combining monetary and non-monetary methods can capture a broader range of value [10], while deliberative approaches such as MCDA may accommodate stakeholder diversity without requiring systematic monetization [6,34]. This combination can improve transparency, make anthropocentric assumptions explicit, and support institutional vigilance against commodification pathways [53,54] (S1 Fig).

## Materials and methods

### Research design and data collection

The primary research question of this meta-analysis was “In agriculture and agroforestry, how did different methods for estimating the economic value of ecosystem services vary in terms of effectiveness and applicability across various contexts?” To address this research question, the scope was defined by the Population/Problem, Intervention/Practice, Comparison, and Outcome (PICO) components, as presented in Table 1. To ensure methodological rigor, two independent reviewers (MMO and CPK) screened studies for eligibility and extracted data. Discrepancies were resolved through discussion and, when necessary, adjudication by a third reviewer (AS), thereby reducing potential bias in the analysis. A detailed codebook was developed and applied to define valuation methods, ecosystem-service categories, units, and standardization rules, ensuring consistent coding (see S1 Appendix for the codebook).

**Table 1:** PICO framework for systematic review.

Population/Problem	Intervention/ Practice	Comparison	Outcome
Agricultural systems, including agroforestry and livestock practices, that provide ecosystem services	Various methods used to estimate the economic value of ecosystem services (e.g., CVM, market pricing, CBA)	Comparison of different economic valuation methods or comparison of economic values derived from these methods across different agricultural contexts	The estimated economic value of ecosystem services, including the effectiveness and applicability of different valuation methods

To address the above research question, the Boolean search keyword combinations used for records in the databases were as follows: “economic value” OR “valuation methods” AND “ecosystem services” OR “agriculture” OR “agroforestry” AND “contingent valuation” OR “market pricing” OR “cost-benefit analysis” NOT “theoretical” OR “review” OR “opinion”. Electronic academic databases included in the search for relevant items were Google Scholar, Scopus, ISI Web of Science, BASE, Lens.org, Agris, Crossref, Semantic Scholar, JSTOR, Directory of Open Access Journals (DOAJ), Econpapers, and Scientific Research. The literature search was conducted between April and June 2025 and focused on global-scale studies without a time frame. Titles and abstracts were first imported into EndNote, where the initial duplicate references were removed. The data were then transferred to JabRef for a second round of duplicate merging. Finally, the remaining duplicates were manually identified and removed using Excel, resulting in a clean database ready for the screening process.

### Study inclusion and exclusion criteria and screening process

The PICO format was used to define the inclusion criteria. We then applied the inclusion criteria to the publication titles and keywords during filtering. After this process, the abstracts were assessed, the remaining publications were filtered, and the entire document was reviewed. Following Pullin and Stewart [70], whenever doubts arose about inclusion, the case was retained for the next stage of evaluation. To check for data quality and consistency in applying the inclusion criteria, two other reviewers conducted a scoping exercise of 10% of the references [70,71]. The inclusion criteria were applied in a stepwise process, as outlined in Table 2. For this step, a standardized protocol was used. Disagreements between reviewers (MMO, CPK, and AS) were resolved first by consensus and, when consensus could not be reached, through consultation with two other reviewers (TEE and VP). This escalation process was intended to support consistent application of the inclusion criteria and reduce potential selection bias.

**Table 2:** Study inclusion and exclusion criteria.

	Inclusion criteria	Exclusion criteria
**Relevance to topic**	Studies must directly address the economic value of ecosystem services, specifically in the contexts of agriculture, agroforestry, or livestock systems	Studies not focused on the economic valuation of ecosystem services, or those unrelated to agriculture, agroforestry, or livestock
**Type of study**	Empirical research articles, case studies, and systematic reviews that present quantitative or qualitative estimates of economic value and utilize recognized economic valuation methods	Theoretical papers, opinion pieces, and editorials that lack empirical data or economic valuation, and studies that focus solely on ecological assessments without economic implications
**Geographic scope**	Research from any geographic location is eligible, with no regional or country restrictions, to ensure broad and diverse representation of contexts	Research that lacks a specific geographic context or is based on highly localized studies that do not contribute to a broader understanding
**Publication type**	Acceptable sources include peer-reviewed journal articles, conference papers, and reports from reputable organizations or institutions	Non-peer-reviewed articles, book chapters, dissertations, and grey literature, unless they provide significant insights or data not available in peer-reviewed sources
**Language**	Studies published in English or French	Studies not published in English or French
**Method**	Studies that employ recognized or robust economic valuation methods (e.g., contingent valuation, market pricing, CBA, hedonic pricing, travel cost method, choice modeling, benefit transfer)	Studies that do not employ recognized or robust economic valuation methods, or that lack transparency in their valuation methods

This systematic review used the Preferred Reporting Items for Systematic Reviews and Meta-Analysis (PRISMA) protocol to select studies [72]. S2 Fig outlined the search and screening process used to identify the included articles (see S2 Appendix for the completed PRISMA checklist).

The final dataset comprises 146 studies published between 1999 and 2025 (see S3 Appendix for the full list), from which 61 effect sizes were derived. Here, each effect size is defined as a unique publication-year × continent observation. Asia (n = 20) and Europe (n = 15) are the most represented regions, followed by North America (n = 8), Oceania (n = 8), Africa (n = 7), and South America (n = 3). Regarding the ecosystem services covered, provisioning services are represented in 50 of the 61 effect sizes (82.0%), regulating services in 40 (65.6%), cultural services in 17 (27.9%) and supporting services in 7 (11.5%); percentages exceed 100% because several effect sizes cover more than one service category. Agricultural systems are specified for 17 effect sizes (27.9%) across five categories: Agroforestry (n = 5), conventional arable land farming (n = 5), conventional farming (n = 4), community gardens (n = 4), and citrus plantations (n = 3). Because some effect sizes were assigned to more than one agricultural system category, these counts are not mutually exclusive. For the remaining effect sizes, the underlying studies did not report the production system in sufficient detail to allow classification.

### Data extraction strategy

To perform a meta-analysis, data on each ecosystem service were extracted from each publication, including both quantitative and qualitative data. The risk of bias was evaluated using a modified ECOBIAS checklist [73], an established instrument specifically tailored for economic assessments. Two reviewers (MMO and CPK) independently scored each study on 16 items across four domains: study design, data and methods, analysis, and reporting transparency. A third author (AS) resolved any conflicting extractions, and two other authors (TEE and VP) made the final decision on the process with MMO, CPK, and AS. Studies were classified as low risk of bias (65.07%), moderate risk (31.51%), and high risk (2.74%) (see S4 Appendix for the Risk of Bias Assessment Using Adapted ECOBIAS Checklist). If the data from the publications were validated by each author, but summary statistics were not available in the text, data were extracted from tables and graphs or calculated from available raw data. All extraction and processing scripts were implemented in R (version 4.5.1). The workflow used magick for image import and pre-processing, pdftools for reading native and scanned PDF files, openxlsx for exporting structured Excel outputs, dplyr and stringr for data manipulation and text parsing, and png for pixel-level figure reading. Optical character recognition was performed with Tesseract OCR (version 5.5.1; UB Mannheim Windows build) using the English and French language models (eng, fra). Graphical data were digitized with WebPlotDigitizer (version 5; Rohatgi, Automeris; accessed August 2025) [74,75]. GPS coordinate data were compiled using standardized country names matched to reference coordinates in Flourish for visualization and harmonization only; no spatial analysis was performed in Flourish [76,77]. The harmonized coordinates were then imported into GeoDa for all subsequent spatial analyses.

Following the review objectives and the PICO framework, and informed by Ignatyeva et al. [78], Kaval [79], Kay et al. [80], Thomasz et al. [81], Mohammadyari et al. [82], and Tinch et al. [65], the data extracted form captured the following variables from each publication: author(s), year of publication, study type (empirical research, etc.), geographic location, continent, ecosystem services valued (carbon sequestration, water purification, biodiversity, etc.), estimation or valuation method (contingent valuation, market pricing, cost-benefit analysis, etc.), stated rationale for method choice when reported (reasons for choosing it over others, if mentioned), estimated economic values, units of measurement (USD per hectare, per year, etc.), agricultural system or practice (conventional farming, agroforestry, livestock management, etc.), reported strengths or weaknesses of the method used (if mentioned), comparisons with other studies when available, valuation approach (value-based approach, scoring-based approach, normative, etc.), key findings, recommendations, limitations, and suggestions for future research.

Monetary values reported in currencies other than USD were converted to a common USD basis to enable cross-study comparison. Conversions were performed during database construction using publicly available exchange-rate sources at that time (see S1 Appendix for the codebook). Values reported per unit area or in local measurement units were standardized using the corresponding conversion factors (e.g., 1 mu = 0.0667 ha). The extracted data included estimates of central tendency, confidence intervals, and value ranges. When studies did not report means directly, central values were estimated from the reported confidence intervals or ranges. For studies reporting only a range, the representative value was calculated as the arithmetic mean of the lower and upper bounds. For each entry, the original reported value and the standardized USD value were retained in separate columns, preserving the source estimate unchanged.

### Data analysis

Frequency analyses were conducted to identify the most frequently used methods, and sunburst plot were generated to visualize the geographic distribution of method use. Temporal trends in method preferences were assessed by plotting annual frequencies over publication years to identify shifts in research focus and method adoption [9,83,84].

To further explore valuation method trends and interdisciplinary linkages, a graphical network analysis of the relationships between the ecosystem services and valuation methods reported in the reviewed studies was conducted using the igraph package in R [85]. We identified research hotspots and emerging areas of focus in the field. We performed a Kruskal-Wallis rank sum test and Dunn’s tests after checking for normality (Shapiro test) and homogeneity (Levene test) assumptions to compare the precision and magnitude of value estimates across valuation methods and service categories [86,87] after splitting the database with a method-conversion unit variable. The meta-analysis assessed the effects of ecosystem categories, agricultural systems, and valuation methods on monetary value estimates. Random-effects models were used to pool mean values for each ecosystem service, accounting for heterogeneity across studies. Publication bias was assessed using Egger’s test [88,89]. I^2^ statistics were used to quantify and explore sources of heterogeneity [90]. To evaluate the influence of valuation methods, agricultural systems, and ecosystem service categories on reported economic values across the reviewed studies, spanning six continents (Asia, Europe, North America, Oceania, Africa, and South America) from 1999 to 2025, we operationalized the effect size as the mean of log10-transformed ecosystem service values per year, calculated within each year-by-continent group [91]. In this study, ‘effect size’ refers to the mean magnitude of ecosystem-service economic values within each analytical context (year and continent), rather than to a between-group comparative measure. Geary’s test was used to assess the normality of the log-transformed effect size distribution [92]. There were 61 distinct year–continent combinations among the 146 included studies, corresponding to an overall mean of approximately 2.39 studies per combination. As comparable sampling variances or standard errors were not consistently reported across observations, inverse-variance weighting could not be applied reliably. We therefore used unweighted arithmetic means of the log-transformed values within each year_continent group and modeled between-group heterogeneity using a multivariate random-effects model estimated by restricted maximum likelihood (REML).

Accordingly, after calculating the log10-transforming the economic values, the effect size (ES) for each year-by-continent group was calculated as follows:


$$\begin{array}{c} {ES}_{j}=\frac{\text{1}}{n_{j}}\sum_{j=\text{1}}^{n_{j}}{log}_{\text{1}\text{0}}\;(Y_{ij}) \end{array}$$ (1)

where ${ES}_{j}$ is the effect size for group j (defined by year and continent), $n_{j}$ is the number of observations in the group j, and ${log}_{\text{1}\text{0}}\;(Y_{ij})$ are the individual log-transformed ecosystem service values from study i within that group j.

As a fixed-effect model does not account for between-study differences, it is less appropriate than the preferred random-effects model [93]. The multivariate random-effects meta-regression analysis, using a restricted maximum likelihood (REML estimator [94,95], incorporates year_continent as a random effect (while keeping conversion unit as a fixed moderator like other independent variables), where the statistical model is:


$$\begin{array}{cc} {ES}_{j}= & \;\beta_{\text{0}}+\beta_{\text{1}ij}{\left(Agricultural\;system\right)}_{ij}+\beta_{\text{2}ij}{\left(Valuation\;method\right)}_{ij}\\ & +\beta_{\text{3}ij}{\left(Category\;of\;ecosytem\;services\right)}_{ij}+\beta_{\text{4}ij}{\left(Conversion\;unit\right)}_{ij}+\mu_{j}+\varepsilon_{ij} \end{array}$$ (2)

$\beta_{\text{0}}$is the intercept; $\beta_{\text{1}ij}\;,\;\beta_{\text{2}ij},\;\beta_{\text{3}ij},\;\beta_{\text{4}ij}\;$are coefficients for each independent predictor (agricultural systems, valuation methods, conversion unit, ecosystem service categories); ${ES}_{j}$ is the effect size for group j (where group j is Year_Continent); $\mu_{j}\;\sim \;N(\text{0},\;\tau^{\text{2}})$ is the between-study random effect for group j, representing heterogeneity, and $\varepsilon_{ij}\;\sim \;N(\text{0},\;{SE}_{ij}^{\text{2}})$ is the within-study sampling error.

The moderators were coded at the level of the individual estimate as binary indicators (1 = attribute present, 0 = absent) for each ecosystem-service category, each agricultural system, each valuation method, and each value unit. Within each year-by-continent group, these binary indicators were then summed, so that each moderator variable expresses the number of estimates in the group exhibiting the respective attribute. A moderator value of 0 therefore indicates that the attribute does not occur in that group.

To assess multicollinearity among the moderators, variance inflation factors (VIFs) were calculated [96]. We then conducted two reduced models as sensitivity analyses: the first included only the moderators that were statistically significant in the full model, while the second included the non-significant moderators [97,98]. The models were compared using the Akaike Information Criterion (AIC), which penalizes model complexity.

Spatial analysis was performed to map the geographic distribution of studies and their associated estimated values. Choropleth maps and geospatial interpolation techniques were employed to identify regional clusters and gaps in ecosystem valuation methods. Temporal trends in value estimates were visualized using LOESS curves with a serial alpha-adjusted t-test approach, which accounted for temporal dependencies and the smoothing nature of LOESS [19,99].

We systematically assigned coded categories for the reported strengths, weaknesses, and limitations of each ecosystem valuation method. A stacked bar chart and a network visualization were used to present the prevalence of specific limitations and to compare the robustness of the evaluation methods [13,100]. All statistical analyses and visualizations were conducted in R (v.4.5.2) and Microsoft Excel 2019, with spatial analyses in GeoDa (v.1.22.0.14). The supporting figures and appendices are archived on Zenodo (https://doi.org/10.5281/zenodo.22900851), and the datasets, scripts, and tools used for the analyses are available at https://doi.org/10.5281/zenodo.22900698.

## Results

### Valuation methods distribution and trends

The analysis of valuation methods reveals a marked predominance of MPV and CVM, which dominate the landscape of valuation studies (S3 Fig). Both methods, followed by the benefit transfer method (BTM) and the choice experiment (CE) constitute the main pillars of economics assessments of ecosystem services. The large proportion of MPV and CVM underscores their central role in capturing direct market values and stated preferences for non-market services, respectively.

S4 Fig reveals notable geographic disparities in the adoption of valuation methods. Asia, particularly China, shows a clear preference for CVM and MPV, whereas research in Africa, especially in Ethiopia, relies predominantly on BTM. Europe shows marked diversity in method selection, with no single approach strongly dominating.

Trend analysis (S5 Fig) indicates a gradual rebalancing among valuation-method families rather than the replacement of any single technique. Stated-preference methods, particularly CVM and CE, dominated earlier periods but declined in relative terms, while revealed-preference methods expanded, mainly through the steady rise of MPV, which is now the most frequently used individual method. CBA appeared intermittently throughout the period and retained an auxiliary role. Recent years show renewed use of BTM and the emergence of integrative approaches, notably LCIA, alongside the first use of the life satisfaction approach (LSA), suggesting a shift toward more standardized, multidisciplinary assessment. Spatial (SITE) and environmental modelling (EM) remained occasional, indicating limited uptake of computational tools.

### Research hotspot and network analysis

Network analysis (S6 and S7 Figs) identifies CVM and LCIA as economic evaluation central methods. These methods are distinguished by their high connectivity and their ability to link the full range of ecosystem service categories, thereby acting as key bridges in interdisciplinary research. Emerging methods such as the economic assessment model (EAM), life cycle costing (LCC), and the net primary productivity approach (NPP) also appear to have the potential to connect distinct research domains.

### Method effectiveness and contextual drivers

Analysis of the units of measurement (S8 Fig) reveals distinct normalization patterns across service categories. For CULT services, non-spatial monetary units predominate. In contrast, PROV and REG services are more frequently normalized spatially (USD/ha), highlighting the importance of land area in their valuation. SUP services exhibit a distinct diversity, incorporating both area-based and mass-based units.

S9 Fig shows substantial variability in value estimates. PROV and REG services span a broad range and have a high density of log-transformed values, suggesting that economic valuation methods are more readily applied to these service categories. By contrast, CULT and SUP services are assessed with fewer methods and show a narrower, sparser distribution of values, indicating that these categories remain more difficult to value consistently.

As shown in S10 Fig, PROV services exhibit the highest median economic value, which may reflect a closer alignment of current methods with services directly linked to markets rather than greater intrinsic effectiveness. REG services occupy an intermediate position, whereas CULT and SUP services show statistically similar and lower median values; this pattern may also arise from the nature of the available indicators and the heterogeneity of the contexts examined, rather than solely from undervaluation.

PROV services exhibit the highest median economic value (S10 Fig), which may reflect a closer alignment of current methods with services directly linked to markets rather than greater intrinsic effectiveness. REG services occupy an intermediate median value (S10 Fig); which may also reflect the nature of the available indicators and the heterogeneity of the contexts examined, rather than solely undervaluation. S11 Fig illustrates that the methods family comprising MPV and CBA tends to yield higher median valuations. In contrast, stated preference methods such as CVM and the avoided cost method generally produce more conservative estimates, clustering in lower median-valuation categories.

### Spatio-temporal valuation patterns

The choropleth maps (S12 Fig) illustrate the geographic distribution of valuation data, with spatial disparities in valuation methods. However, the spatial autocorrelation analysis, with Moran’s I values close to zero (S13 Fig), indicates no significant global spatial clustering of valuation levels. This pattern suggests that local contextual factors, such as agricultural practices or governance, are more likely to explain valuation variation than geographic proximity alone. The cluster map (S14A Fig) and the Gi* significance map (S14B Fig) further support the lack of strong spatial structure, with only a few regions exhibiting significant clustering.

The LOESS trend, accompanied by bootstrapped 95% confidence intervals, illustrates how the mean (log10) ecosystem service values changed from 1999 to 2025, generally following an upward but variable trajectory (S15 Fig). Statistically significant deviations from the trend, identified using a serially adjusted t-test and marked in red, indicate episodic peaks (notably in the early 2000s and around 2010) as well as downturns that may reflect shifts in valuation methods. This pattern suggests that economic valuations in agriculture and agroforestry fluctuate over time rather than following a consistent trend.

### Meta-analysis of economic valuation of ecosystem services

The meta-analysis indicates that the synthesized monetary value estimates for ecosystem services in agriculture and agroforestry (Table 3) exhibit substantial heterogeneity across all examined categories (CULT, PROV, REG, and SUP). This suggests that most of the observed variability reflects between-study differences rather than sampling random error. Egger’s test suggested potential publication bias in the CULT, PROV, and REG categories, whereas no evidence of such bias was detected for SUP services.

**Table 3:** Meta-analysis of economic valuation per category of ecosystem services.

Categories of ecosystem services	I^2^ (%)	Tau^2^ (Variance)	Egger’s p-value	Effect size (n)
**CULT**	100	0.468	0.0112	17
**PROV**	100	0.61	0.0000	50
**REG**	100	0.854	0.0039	40
**SUP**	100	0.298	0.4511	7

S16 Fig summarizes the composition of the agricultural systems included in the dataset. Perennial cash crops and annual crops exhibit the greatest internal heterogeneity, while within agroforestry, complex and general systems are the most prevalent. This distribution indicates that the analysis encompasses a broad range of agricultural contexts. Statistical analysis (S17 Fig) did not detect significant differences in ecosystem service values among agricultural system types after within-unit standardization (H = 6.00, df = 7, p = 0.539).

Most variables showed acceptable VIFs. However, USD.month had a severe VIF (>10; VIF = 10.17), indicating substantial collinearity with other predictors; therefore, it was excluded from subsequent analyses (see S5 Appendix for the metaregression report with VIF and AIC). Most variables showed acceptable VIFs. A full model including all moderators (AIC = 299.32, pseudo-R^2^ = 59.58%), a reduced model including only the moderators that were significant in the full model (AIC = 286.89, pseudo-R^2^ = 38.48%), and a reduced model including the remaining non-significant moderators (AIC = 314.90, pseudo-R^2^ = 30.22%) were then compared. Although the first reduced model had the lowest AIC, it explained less heterogeneity than the full model. Accordingly, the full model may be preferable if the aim is broader moderator adjustment, whereas the first reduced model is preferable only under an AIC-based parsimony criterion (Table 4).

**Table 4:** Multivariate random-effects meta-regression (REML estimator).

Term (sample size)	Group	Full model	Reduced model with significant coefficient estimates from the full model	Reduced model with non-significant coefficient estimates from the full model
		Estimate ± Standard error (95% confidence intervals)		
Intercept	Other	3.82*** ± 0.72 (2.41, 5.23)	3.33*** ± 0.39 (2.55, 4.10)	3.45*** ± 0.55 (2.37, 4.54)
PROV (50)	Ecosystem	−0.40 ± 0.37 (−1.12, 0.33)		0.04 ± 0.25 (−0.44, 0.52)
REG (40)		−0.85 ± 0.55 (−1.94, 0.23)		0.58* ± 0.35 (−0.10, 1.26)
CULT (17)		−1.57** ± 0.74 (−3.02, −0.12)	−0.59 ± 0.40 (−1.38, 0.20)	
SUP (7)		−0.05 ± 1.21 (−2.42, 2.32)		1.02 ± 0.95 (−0.85, 2.88)
MPV (21)	Valuation	0.86* ± 0.47 (−0.07, 1.79)	0.21 ± 0.23 (−0.24, 0.65)	
CVM (24)		−0.02 ± 0.40 (−0.79, 0.76)		−0.53* ± 0.29 (−1.11, 0.04)
BTM (6)		1.19* ± 0.61 (−0.01, 2.39)	1.19*** ± 0.35 (0.51, 1.87)	
CE (12)		−0.01 ± 0.44 (−0.87, 0.85)		−0.48 ± 0.34 (−1.15, 0.18)
ACM (3)		1.90* ± 0.98 (−0.02, 3.82)	0.96 ± 0.75 (−0.50, 2.42)	
Total economic value (3)		0.89 ± 0.87 (−0.81, 2.60)		0.18 ± 0.79 (−1.36, 1.73)
CBA (4)		1.32 ± 0.90 (−0.45, 3.08)		−0.83 ± 0.69 (−2.18, 0.52)
Bio-economic approach (3)		−2.27 ± 1.47 (−5.15, 0.61)		−1.59 ± 1.05 (−3.64, 0.47)
Replacement cost approach (3)		0.51 ± 1.09 (−1.62, 2.64)		−0.22 ± 0.87 (−1.93, 1.48)
LCIA (3)		2.94** ± 1.46 (0.08, 5.80)	0.38 ± 0.60 (−0.80, 1.57)	
Double-bounded dichotomous choice (2)		1.26** ± 0.62 (0.05, 2.47)	0.44 ± 0.46 (−0.46, 1.33)	
Travel-cost method (4)		1.12 ± 0.81 (−0.48, 2.71)		0.48 ± 0.64 (−0.77, 1.74)
USD/ha (22)	Conversion	0.05 ± 0.46 (−0.85, 0.95)		−0.18 ± 0.34 (−0.85, 0.49)
USD (25)		1.05*** ± 0.41 (0.25, 1.85)	1.01*** ± 0.26 (0.49, 1.53)	
USD/year (18)		0.53 ± 0.45 (−0.35, 1.40)		0.73** ± 0.33 (0.09, 1.36)
USD/ha/year (15)		−1.63** ± 0.65 (−2.90, −0.35)	−1.08*** ± 0.38 (−1.83, −0.33)	
USD/person/year (5)		0.53 ± 0.50 (−0.45, 1.51)		0.09 ± 0.45 (−0.81, 0.98)
Agroforestry (5)	Agricultural	1.18 ± 1.18 (−1.14, 3.49)		1.74* ± 0.91 (−0.04, 3.53)
Conventional arable land farming (5)		−0.32 ± 1.53 (−3.32, 2.69)		0.34 ± 1.28 (−2.17, 2.86)
Citrus plantations (3)		−0.37 ± 1.24 (−2.80, 2.06)		0.74 ± 0.98 (−1.18, 2.67)
Conventional farming (4)		2.60 ± 1.58 (−0.51, 5.70)		1.59 ± 1.32 (−1.00, 4.18)
Community gardens (3)		0.90 ± 1.64 (−2.33, 4.12)		−0.87 ± 1.37 (−3.55, 1.82)
**Model validation statistics**				
k (p fixed moderators)		61 (27)	61 (9)	61 (19)
Moderators heterogeneity (QM)		85.37***	37.02***	25.13
Residual heterogeneity (QE)		456808.36***	1847792.21***	1335150.47***
τ²		2.96	4.50	5.11
I^2^		99.98	99.99	99.99
AIC		299.32	286.89	314.90
ΔAIC		12.42	0.00	28.01
Pseudo-R² (%)		59.58	38.48	30.22

*p < 0.1, **p < 0.05, ***p < 0.01. The sample size for each variable corresponds to the number of the 61 effect sizes in which that variable was coded (> 0). The dependent variable is the effect size (ES), defined as the mean log_10_-transformed economic value (USD) for each year-by-continent group. Accordingly, all coefficient estimates, standard errors, and confidence intervals are expressed in log_10_(USD) units. Each coefficient represents the change in the log_10_-transformed economic value associated with a one-unit increase in the corresponding moderator. Because the scale is logarithmic, estimates can be back-transformed into multiplicative terms (ratios of means) as 10^β^ (e.g., an intercept of 3.82 corresponds to 10^3.82^ ≈ 6.6 × 10^3^ USD in the full model).

Considering a full model, the intercept (β = 3.84, p < 0.001) indicates a strong positive baseline effect size, while CULT services show significantly lower valuations (β = –1.57, p = 0.033), highlighting challenges in capturing their economic value (Table 4). Several methods like MPV, BTM, ACM, LCIA, and double-bounded dichotomous choice are linked to higher effect sizes, and values expressed in USD are significantly larger than those standardized per hectare per year. However, high residual heterogeneity (I^2^ = 99.98%, τ^2^ = 2.96) suggests that unobserved contextual factors still drive much of the variation, emphasizing the importance of method choice and unit standardization when valuing ecosystem services in agriculture and agroforestry.

Most studies show positive effect sizes, with only a few small negative values, indicating a generally positive valuation of ecosystem services across contexts (S18A Fig). However, the wide variation in confidence interval widths and effect magnitudes highlights significant heterogeneity and the need to account for it using moderator variables and random effects, even though the overall trend is consistent with the pooled-average effect from the meta-regression model. The roughly symmetric clustering of points around the summary effect size suggests no strong publication bias or small-study effects in the meta-analysis (S18B Fig). High-precision studies lie near the center; while lower-precision studies are more dispersed, as expected. The balanced spread of positive and negative effect sizes, including a few significant, high-precision estimates consistent with the forest plot, supports the robustness of the overall findings and reduces concerns about selective reporting bias.

### Strengths and weaknesses of valuation methods

S19 Fig compares the strengths and limitations of various valorization methods. MPV has stronger empirical grounding, although it shows only moderate performance in comprehensiveness, policy relevance, and practical feasibility. Its main limitation lies in data scarcity and context sensitivity. By contrast, CVM is empirically robust but constrained by limited data and context dependence, which can limit the generalizability of its findings despite its widespread use.

## Discussion

### Effectiveness and valuation method biases in valuing ecosystem services

Eppink et al. [101] discovered that providing clear monetary information enhances both the pursuit of economic advantages and the prevention of environmental harm. Our results confirm that MPV, CVM, BTM, and CE are widely used, but their effectiveness is context-dependent [29,102–108]. As noted by Haab et al. [109], economists generally prefer revealed-preference methods for valuing the environment because these techniques are based on actual observed behavior. In particular, MPV, as a revealed-preference approach, is highly context-dependent, where preferences may change over time and market imperfections can distort estimated values [19]. This interpretation is consistent with the neoclassical economic model of *Homo economicus*, which assumes that individuals act independently to maximize utility based on relatively stable preferences [110]. Tinch et al. [65] suggest that prices can be adjusted to more accurately reflect social costs and benefits, even in situations where markets fail. The observation by Legesse et al. [19] that non-market valuation methods (CVM, CE) have been applied to estimate ecosystem services across PROV, REG, CULT and SUP categories is consistent with our results. Santoyo et al. [111] noted that CVM is among the most commonly used methods for estimating the value of public goods and services and is widely applied by government institutions. Nijnik and Miller [33] mentioned that BTM is fairly simple to comprehend and implement, which could explain its widespread use in valuation. Richardson et al. [112] further argued that benefit transfer requires careful consideration of diminishing marginal utility, relative scarcity, and substitutability because the method relies on judgment when transferring welfare estimates across contexts. In general, values estimated using benefit transfer and production function methods tended to be higher, while those derived from market pricing were lower, according to Schild et al. [87]. Stutzer and Frey [113] argued that revealed-preference approaches cannot distinguish fully between rational consumer choice and cases in which individuals mispredict utility or face self-control problems. In that context, they identified LSA as a promising emerging method. Our trend analysis likewise indicates increasing use of life-satisfaction approaches, plausibly reflecting their perceived co-benefits and synergies, consistent with VanderWilde and Newell [114]. Frey et al. [115] further noted that LSA builds on subjective well-being research in economics by using reported life satisfaction as an empirical approximation of individual welfare. Li [116] showed that predictive methods are increasingly used to generate spatial predictions across environmental science disciplines. However, the most appropriate valuation method for a given service depends on the valuation purpose as well as the socio-economic and environmental context, as emphasized by Groot et al. [54]. This study demonstrates that the selection of the valuation approach accounts for a greater portion of the variation in monetary values than the type of ecosystem or system. It is consistent with Schild et al. [117], who reported that methodological factors, including valuation method and study extent, strongly influence ecosystem services values. Taken together, these results indicate that variation in monetary valuation outcomes is more strongly associated with methodological choices than with ecosystem characteristics alone.

PROV and REG services are more easily and frequently valued, reflecting their tangible outputs and direct market linkages, whereas CULT and SUP services remain challenging to value and are often undervalued [29,102–107,118,119]. This supports Liu [120], who found that PROV services are valued most highly due to their immediate relevance to people’s well-being and livelihoods, whereas CULT services are valued least, possibly because their importance is less frequently expressed in direct economic terms. VanderWilde and Newell [114] likewise concluded that provisioning, regulation and maintenance ESs have received the most attention to date. Amberber et al. [121] found that PROV services received the most focus among all categories, making up 40.29%, while CULT services were relatively less emphasized, accounting for only 5.97%. This pattern is consistent with previous meta-analyses and reviews, which highlight the dominance of market-based and stated-preference methods and the persistent difficulty in capturing non-market services [102,104–108,118]. Although methodological challenges remain, Plieninger et al. [122] argued that cultural services mapping assessments should be advanced as essential components of cultural landscape management and protection. At the same time, ecosystem services are interdependent, and their relationships are likely highly nonlinear [123]. Soldati et al. [124], therefore called for further research on cultural and supporting services, which remain especially neglected in the literature. In Schmidt et al. [125], 18% to 44% of the variance in monetary values was explained by the selected ecosystem service category, supporting the idea that valuation outcomes are strongly service-dependent.

### Contextual influences and challenges with valuation methods in ecosystem service valuation

The heterogeneity in valuation results underscores the influence of contextual factors, valuation method choices, and unit standardization, reinforcing the notion that ecosystem service values are not universally transferable without careful adaptation [102,104,107,126,127]. Power [123] stated that the values of both market and non-market goods and services vary with biophysical and socioeconomic conditions, indicating that ecosystem service valuation is inherently context dependent. The predominance of MPV and related approaches for tangible services likely reflects their compatibility with existing economic data, while the lower valuations for intangible services point to the need for valuation methods and the integration of socio-cultural perspectives [124,126–129]. Richardson et al. [112] showed that the scarcity and substitutability of ecosystem service flows vary with spatial scale, further underscoring the importance of scale in valuation outcomes.

Legesse et al. [19] argued that the appropriateness of ecosystem valuation methods varies across conditions and methodological constraints. Our results indicate that CVM is empirically robust yet sensitive to methodological choices. This interpretation is consistent with Bostan et al. [130], who found that CVM values aligned with CE, while estimates varied by elicitation format, yielding total values of $97,107.36 and $82,194.09, respectively. Following Eregae et al. [131], the lower comprehensiveness of market price-based valuation methods such as MPV in our findings may reflect the fact that market prices do not capture the full range of ecological values needed to support rational decision-making in ecological conservation. Grounded in *Homo communicus*, where value originates from community consensus and equity rather than private utility, this perspective suggests that conventional survey methods such as CVM may be limited for assessing fairness because they register individual preferences rather than preferences formed through democratic deliberation [110]. Relatedly, *Homo naturalis* emphasizes an ecosystem’s functional contribution to overall system survival, independent of short-term human awareness [110]. From this viewpoint, market-based or stated-preference approaches like MPV and CVM are unable to completely reflect the value rooted in sustainability, since essential ecosystem functions, such as climate regulation and nutrient cycling, occur beyond what individuals can perceive or what is available in the market [110]. More broadly, Costanza [110] argued that a method measuring individual willingness to pay (E-value) should not be treated as a proxy for ecological sustainability (S-value) or social justice (F-value), because these dimensions rest on different evaluative foundations, with context dependency constraints issue. Similarly, Schmidt et al. [125], argued that assuming ecosystem homogeneity is a central error in value-transfer models, because assigned values depend strongly on the local socio-ecological context.

### Policy implications and regional adaptation in ecosystem service valuation

Policy frameworks that rely heavily on economic valuations must recognize the potential undervaluation of intangible services, which are critical for agroecosystem sustainability and resilience [29,102–108,118,119]. The demonstrated regional variability in method usage, shaped by data availability and socio-economic context, suggests that blanket recommendations for valuation techniques are ineffective. Instead, adaptive frameworks that account for local conditions and capacity constraints, especially in resource-limited regions (e.g., Africa), are recommended [121,132,133]. The systematic synthesis of peer-reviewed studies across continents and service types, supplemented by meta-analysis and network analyses, enhances confidence in these findings. Potential biases, such as hypothetical bias in stated-preference methods and data constraints in BTM, warrant cautious interpretation of the comparative effectiveness [106,108].

### Research contribution

This study makes four key contributions: it quantifies extreme heterogeneity and publication bias in economic valuations of agricultural and agroforestry ecosystem services, demonstrating that nearly all variation in values results from differences in methods, contexts, and unit choices rather than chance, and it shows a persistent bias toward higher and more frequent valuation of PROV and REG services because of their tangible outputs and market connections [29,102,132,134,135]. It also highlights the importance of normalization units (with per-hectare-per-year estimates providing more comparable data than lump-sum USD figures). It advocates for integrated, adaptive valuation frameworks that explicitly consider strong context dependency and low spatial autocorrelation, since local ecological and socio-economic conditions are more influential than broad regional patterns in shaping valuation outcomes [102,132,136].

### Novelty of research and approach

The added value and novelty of this study lie in its quantitative meta-analytic framework, which not only identifies valuation methods but also empirically demonstrates the drivers and the extent of heterogeneity and publication bias, showing how choices such as valuation method and unit standardization shape the magnitude and reliability of economic value estimates. Going beyond previous reviews that merely noted high variability [29,137]. This study provides statistical evidence of extreme heterogeneity (I^2^ = 100%) and large unexplained variance (93.06%). Moreover, through spatial analysis, it shows that this residual variation reflects highly local, context-specific factors rather than broad regional patterns, thereby offering a new quantitative basis for shifting ecosystem service valuation toward a stronger focus on local conditions.

Future research should develop hybrid valuation frameworks that combine market data with robust preference-elicitation methods and explicitly incorporate spatiotemporal dynamics to reduce uncertainty [124,126–128]. Given the low spatial autocorrelation observed, long-term, cross-scale, and highly local case studies are needed to disentangle ecological, socio-economic, and cultural drivers of valuation outcomes and to generate the nuanced evidence required for effective ecosystem management.

### Limitations

Exchange rates were obtained from online sources at the time the database was assembled, rather than being harmonized to a single reference date, and monetary values were not adjusted for inflation. Accordingly, the standardized USD values should be interpreted as approximate orders of magnitude rather than precise, directly comparable estimates. Therefore, cross-study comparisons of absolute monetary values are indicative rather than definitive.

Although our meta-analysis offers a valuable overview of economic evaluation approaches, the methods examined are primarily anthropocentric and therefore do not capture non-instrumental ethical considerations, such as claims regarding nature’s intrinsic value. Consequently, the analysis reflects utility-based valuations but does not encompass ethical arguments grounded in intrinsic value, protection, or stewardship. As a result, the evaluative scope may be narrower than the full range of considerations relevant to sustainability and equity.

The regression analysis should therefore be interpreted as a meta-regression of aggregated, log-transformed ecosystem service values, with random effects employed to account for hierarchical structure and between-group heterogeneity, rather than as a conventional inverse-variance-weighted meta-analysis.

The results should be interpreted as estimated associations between the fixed moderators and log-transformed ecosystem service values, accounting for clustering by Year–Continent, rather than as precise effects for individual Year–Continent groups.

Though the multivariate random-effects model accounts for heterogeneity across Year–Continent groups, it may not fully capture the dependence among the aggregated observations used to calculate each study’s central value. This limitation could reduce the precision of the variance estimates.

A further limitation concerns the depth of production-system characterization. Studies could be classified by land-use configuration and production logic, but three dimensions that the agricultural economics literature identifies as determinants of ecosystem service value could not be coded: management intensity (input use, mechanization, organic versus conventional practice), operational scale (farm or landscape size, smallholder versus commercial operation), and institutional context (land tenure, market access, subsidy regimes, collective governance arrangements). These attributes are reported inconsistently and most often not at all in the primary studies.

## Conclusions

This study systematically assessed methods for economically valuing ecosystem services in agricultural and agroforestry systems, addressing three key questions: prevalence, effectiveness across contexts, and limitations. Our findings provide important insights with significant theoretical and practical implications. The research shows that valuation approaches differ significantly in their application and effectiveness. Market-based methods are most common for tangible services, while contingent valuation remains crucial for non-market values. Our analysis confirms that valuation method performance is highly context-dependent, with no single approach being universally superior. The study highlights how market price and contingent valuation methods have become standard, but each has notable challenges. Key findings demonstrate that valuation results vary widely across studies, emphasizing the importance of local context in shaping outcomes. Market-based approaches tend to yield higher value estimates than other approaches, while CULT services are often valued lower. This underscores persistent methodological challenges in comprehensively capturing non-market, intangible ecosystem benefits. Valuation units fundamentally influence reported values, with lump-sum USD figures associated with significantly larger effect sizes than standardized per-hectare-per-year values, highlighting the critical role of methodological standardization in valuation interpretation. Spatial analysis offers further depth by showing that, although there are clear geographic patterns in method usage, the valuation results themselves exhibit little spatial autocorrelation and a near-zero Moran’s I statistic, indicating that high or low valuations are randomly distributed rather than regionally clustered. This is reinforced by cluster mapping, which reveals only a few regions with statistically significant spatial clustering. Despite their widespread use, all valuation methods face substantial challenges, with data availability being the most common obstacle across approaches, highlighting the need for continued methodological refinement and context-specific application.

A key finding is the very high heterogeneity in values, which cautions against uniform PES policies based on single valuations or broad meta-analyses and instead calls for flexible, adaptive schemes tailored to local conditions, especially in data-scarce regions. The study also offers practical guidance by showing that standardizing values to a per-hectare-per-year unit produces more conservative, defensible, and comparable estimates for long-term planning. It highlights the ongoing undervaluation of CULT and SUP ecosystem services and emphasizes the need for interdisciplinary, multi-method approaches to more effectively capture these intangible benefits and integrate them into agricultural decision-making and sustainable land management. Achieving truly sustainable decision-making requires complementing monetary valuation with explicit consideration of nature’s intangible and ethical values. More broadly, policy frameworks should adopt an integrated perspective that acknowledges the plurality of ecosystem values (economic, social, cultural, and ethical) to better reflect the complexity and significance of ecosystems.

## Supporting information

S1 Fig This is the conceptual framework of economic valuation. (TIF)

S2 Fig This is the PRISMA flow diagram of the systematic review process. This figure illustrates the screening and selection process for the studies included in the meta-analysis, beginning with database identification (n = 3,347) and progressing through duplicate removal, title/abstract screening, and full-text review, resulting in 146 studies.
(TIF)

S3 Fig This is the percentage of studies using valuation methods. This pie chart illustrates the proportions of studies that use different economic valuation methods for ecosystem services. The most common approaches are market price valuation (MPV, 26%), contingent valuation method (CVM, 21%), benefit transfer method (BTM, 10%), and choice experiment (CE, 7%). Other abbreviations: ACM, avoided cost method; TEV, total economic value; LCC, life cycle costing; BEA, bio-economic approach; GMA, gross margin analysis; DBDC, double-bounded dichotomous choice; OPG, observed prices for goods; MMP, Markov model-based prediction; SEV, simulated exchange value; RP, revealed preference; HPM, hedonic pricing method; MM, mathematical modelling; TCM, travel-cost method; PL, productivity loss; EM, environmental modelling; LSA, life satisfaction approach; EAM, economic assessment model; CBA, cost-benefit analysis; PFA, production function approach; EFAM, equivalent factor accounting method; NPP, net primary productivity approach; LCIA, life cycle impact assessment; RCA, replacement cost approach; EDA, economic decision analysis; NPV, net present value; MA, modelling approaches; PCA, principal component analysis; PCM, productivity change method; SITE, spatial modelling (SITE model); SP, shadow pricing; DUVE, direct-use value estimation; IBEM, integrated biophysical-economic model; ESU, economic surplus method; DRM, dependence ratio method; PEA, partial equilibrium analysis; EXP, expert scoring; RES, residual approach; SM, structural model.
(TIF)

S4 Fig This is the sunburst plot of valuation method usage by continent (in %). This sunburst plot shows the percentage use of different economic valuation methods across continents, highlighting the predominance of contingent valuation (CVM) and market price valuation (MPV) in Asia and the methodological diversity in Europe, Africa, North America, and Oceania. Valuation-method selection differed significantly across continents (Pearson’s chi-square: df = 205, N = 146, χ^2^ = 275.7; Monte Carlo, B = 50000, p < .001; Cramer’s V = 0.615). Other abbreviations: BTM, benefit transfer method; ACM, avoided cost method; TEV, total economic value; LCC, life cycle costing; BEA, bio-economic approach; GMA, gross margin analysis; DBDC, double-bounded dichotomous choice; OPG, observed prices for goods; MMP, Markov model-based prediction; SEV, simulated exchange value; RP, revealed preference; HPM, hedonic pricing method; MM, mathematical modelling; TCM, travel-cost method; PL, productivity loss; EM, environmental modelling; LSA, life satisfaction approach; EAM, economic assessment model; CE, choice experiment; CBA, cost-benefit analysis; PFA, production function approach; EFAM, equivalent factor accounting method; NPP, net primary productivity approach; LCIA, life cycle impact assessment; RCA, replacement cost approach; EDA, economic decision analysis; NPV, net present value; MA, modelling approaches; PCA, principal component analysis; PCM, productivity change method; SITE, spatial modelling (SITE model); SP, shadow pricing; DUVE, direct-use value estimation; IBEM, integrated biophysical-economic model; ESU, economic surplus method; DRM, dependence ratio method; PEA, partial equilibrium analysis; EXP, expert scoring; RES, residual approach; SM, structural model.
(TIF)

S5 Fig This is the share of valuation-method families by period (in %). The bar chart shows the percentage of studies using different valuation methods grouped by method families across periods. Multiple valuation methods are often used within a single study. It demonstrates the evolution from a focus on method concentration in the early years to a greater diversity of evaluation methods in recent years. The Pearson Chi-Square (χ^2^ = 5429.172, df = 943, p < 0.001) indicates significant variation in the use of valuation methods over time. Method families: stated preference (CVM, contingent valuation method; CE, choice experiment; DBDC, double-bounded dichotomous choice; EXP, expert scoring; LSA, life satisfaction approach); revealed preference (MPV, market price valuation; TCM, travel-cost method; ACM, avoided cost method; PFA, production function approach; RCA, replacement cost approach; DRM, dependence ratio method; PCM, productivity change method; EFAM, equivalent factor accounting method; GMA, gross margin analysis; HPM, hedonic pricing method; OPG, observed prices for goods; PL, productivity loss; RES, residual approach; RP, revealed preference; SP, shadow pricing); benefit transfer (BTM, benefit transfer method); and other modelling and integrative approaches (BEA, bio-economic approach; CBA, cost-benefit analysis; TEV, total economic value; LCIA, life cycle impact assessment; DUVE, direct-use value estimation; EAM, economic assessment model; EDA, economic decision analysis; EM, environmental modelling; ESU, economic surplus method; IBEM, integrated biophysical-economic model; LCC, life cycle costing; MA, modelling approaches; MM, mathematical modelling; MMP, Markov model-based prediction; NPP, net primary productivity approach; NPV, net present value; PCA, principal component analysis; PEA, partial equilibrium analysis; SEV, simulated exchange value; SITE, spatial modelling (SITE model); SM, structural model).
(TIF)

S6 Fig This is the network of valuation methods and ecosystem service categories. This figure illustrates the structural relationships among ecosystem service categories and the full range of quantitative and qualitative valuation methods used in ecosystem service assessments. Lines represent the connections between valuation methods and service types, while colours and their shapes indicate valuation approaches and the significance of service categories, respectively. Methods were classified as **quantitative** (MPV, market price valuation; CVM, contingent valuation method; BTM, benefit transfer method; ACM, avoided cost method; LCC, life cycle costing; GMA, gross margin analysis; DBDC, double-bounded dichotomous choice; OPG, observed prices for goods; MMP, Markov model-based prediction; SEV, simulated exchange value; RP, revealed preference; HPM, hedonic pricing method; MM, mathematical modelling; TCM, travel-cost method; PL, productivity loss; LSA, life satisfaction approach; CE, choice experiment; CBA, cost-benefit analysis; PFA, production function approach; EFAM, equivalent factor accounting method; NPP, net primary productivity approach; LCIA, life cycle impact assessment; RCA, replacement cost approach; NPV, net present value; PCA, principal component analysis; PCM, productivity change method; SP, shadow pricing; DUVE, direct-use value estimation; ESU, economic surplus method; DRM, dependence ratio method; PEA, partial equilibrium analysis; RES, residual approach; SM, structural model), **qualitative** (EXP, expert scoring), or mixed quantitative and qualitative (TEV, total economic value; BEA, bio-economic approach; EM, environmental modelling; EAM, economic assessment model; EDA, economic decision analysis; MA, modelling approaches; SITE, spatial modelling [SITE model]; IBEM, integrated biophysical-economic model; CVM; LSA; DUVE).
(TIF)

S7 Fig This is the valuation methods (A) and ecosystem service categories (B) centrality. Panel (A) shows centrality metrics (betweenness, degree, eigenvector, and closeness) for different ecosystem service valuation methods; panel (B) displays the position of ecosystem service categories. Node size indicates closeness centrality, colour represents eigenvector centrality, and position reflects degree versus betweenness centrality. Abbreviations: CVM, contingent valuation method; ACM, avoided cost method; BEA, bio-economic approach; HPM, hedonic pricing method; EAM, economic assessment model; PFA, production function approach; LCIA, life cycle impact assessment; EDA, economic decision analysis; PCA, principal component analysis; RES, residual approach.
(TIF)

S8 Fig This is the distribution of monetary units used in ecosystem service valuation across service categories. The chart displays the top 5 most frequently used monetary units for each of the four ecosystem service categories, expressed as percentages of usage across studies. The variety of units indicates different approaches to quantifying ecosystem service values. Abbreviations: USD, United States dollar; ha, hectare.
(TIF)

S9 Fig This is the distribution of ecosystem service values by valuation method and service category. Scatter plots display log-transformed monetary values (USD) for different valuation methods across four ecosystem service categories. Each point is one study positioned by the valuation method family used and grouped by ecosystem service category. Values are transformed as Log_Value = log10(mean value in USD) for positive values and Log_Value = -log10(|mean value in USD|) for negative values; the latter arises where a study reports a net cost. The transformation is applied to the numerical magnitude, making the scale additive across several orders of magnitude; point colour and shape identify the reporting unit family. Assumptions for parametric testing were not met (Shapiro-Wilk: W = 0.947, p < 0.001; Levene’s test: F(13, 131) = 1.197, p = 0.289).
(TIF)

S10 Fig This is the distribution of ecosystem service value estimates across service categories. Boxes show the interquartile range, horizontal lines the medians, whiskers 1.5 times the interquartile range, and points beyond the whiskers are outliers; overlaid points are individual studies. Each study contributes one estimate (n = 52 studies reporting USD ha-1 or USD ha-1 yr-1); only categories with at least 5 studies are shown. Values are plotted on a log10 scale. Kruskal-Wallis test followed by Dunn’s post hoc test: H = 2.1, df = 1, p = 0.147. Fewer than three categories met the minimum sample size, so no post hoc letters are shown.
(TIF)

S11 Fig This is the magnitude of ecosystem value estimates across grouped ecosystem economic valuation methods. Method codes are grouped into the four families used throughout this study; individual codes are not compared because most are represented by a single study. Boxes show the interquartile range, horizontal lines the medians, whiskers 1.5 times the interquartile range; overlaid points are individual studies. Each study contributes one estimate; only families with at least 5 studies are tested (benefit transfer was therefore excluded). Kruskal-Wallis and Dunn tests: statistic = 19.16, df = 2, p < 0.001. Different letters indicate families that differ in post hoc pairwise comparisons. Method families: stated preference (CVM, contingent valuation method; CE, choice experiment; DBDC, double-bounded dichotomous choice; EXP, expert scoring; LSA, life satisfaction approach); revealed preference (MPV, market price valuation; TCM, travel-cost method; ACM, avoided cost method; PFA, production function approach; RCA, replacement cost approach; DRM, dependence ratio method; PCM, productivity change method; EFAM, equivalent factor accounting method; GMA, gross margin analysis; HPM, hedonic pricing method; OPG, observed prices for goods; PL, productivity loss; RES, residual approach; RP, revealed preference; SP, shadow pricing); and other modelling and integrative approaches (BEA, bio-economic approach; CBA, cost-benefit analysis; TEV, total economic value; LCIA, life cycle impact assessment; DUVE, direct-use value estimation; EAM, economic assessment model; EDA, economic decision analysis; EM, environmental modelling; ESU, economic surplus method; IBEM, integrated biophysical-economic model; LCC, life cycle costing; MA, modelling approaches; MM, mathematical modelling; MMP, Markov model-based prediction; NPP, net primary productivity approach; NPV, net present value; PCA, principal component analysis; PEA, partial equilibrium analysis; SEV, simulated exchange value; SITE, spatial modelling (SITE model); SM, structural model).
(TIF)

S12 Fig This is the choropleth map of the distribution of economic value by valuation method. It shows the geographic distribution of ecosystem service value estimates on a log_10_ scale. Point locations represent individual valuation studies, colored according to quartile ranges: [−1.432: 2.448] (lightest), [2.587: 3.736] (light orange), [4.136: 6.984] (medium orange), and [7.369: 10.893] (darkest orange), with n = 12 observations in each quartile. Grey shading indicates countries or regions, while colored points show the locations and relative magnitudes of the valuation estimates. The map uses a standard global projection with country boundaries outlined. Log_10__Scale values represent log_10_-transformed monetary amounts in USD. Map data source: http://www.naturalearthdata.com/.
(TIF)

S13 Fig This is the Local Indicators of Spatial Association (LISA) cluster map for ecosystem service valuation magnitudes. The scatter plot displays the relationship between log_10_-transformed ecosystem service values (x-axis: Log10_Scal) and their spatially lagged values (y-axis: lagged Log10_Scal). Each blue circle represents a geographic location from the dataset. The purple horizontal and vertical lines intersect at the origin, dividing the plot into four quadrants that identify different types of spatial clustering: high-high (upper right), low-low (lower left), high-low (lower right), and low-high (upper left). Moran’s I = −0.024 indicates negligible spatial autocorrelation in ecosystem service value estimates across the study locations. Map data source: http://www.naturalearthdata.com/.
(TIF)

S14 Fig This is the Getis-Ord Gi spatial clustering (a) and significance map (b) pinpoint regions for geospatial interpolation. Two global maps display spatial clustering patterns of log_10_-transformed ecosystem service values using the Getis-Ord Gi* statistic with spatial weights (weights_kr). (A) The cluster map identifies statistically significant hot spots (High, red points, n = 2), cold spots (Low, blue points, n = 2), and non-significant locations (grey, n = 44). (B) The significance map shows the statistical significance levels of spatial clusters: p = 0.05 (light green, n = 4), p = 0.01 (medium green, n = 0), p = 0.001 (dark green, n = 0), and non-significant locations (grey, n = 44). Points represent individual study locations, with grey shading indicating country boundaries. Map data source: http://www.naturalearthdata.com/.
(TIF)

S15 Fig This is the temporal trend in ecosystem service valuation magnitudes with locally weighted regression (LOESS). The plot displays mean log_10_-transformed ecosystem service values (y-axis) over time from 1999 to 2025 (x-axis). The blue line represents the LOESS-smoothed trend, with the shaded blue area indicating the 95% confidence interval estimated via bootstrap resampling. Black points represent observations that do not significantly deviate from the trend, while red points indicate statistically significant deviations (outliers). The LOESS method captures non-linear temporal patterns in valuation magnitudes without assuming a specific functional form.
(TIF)

S16 Fig This is the distribution of production systems by category. Percentages (< 5%) represent the proportion pooled into “others.” Abbreviations by category. Agroforestry: AF, agroforestry (general); CAF, complex agroforestry; ATF, agrotimber farming; TAF, tree-based agroforestry; APF, agroforestry parkland farming; AFS, agroforestry system; AAF, annual agroforestry farming; AAC, agroforestry with annual crops; AA, agri-agroforestry; AG, agrisilviculture; APS, agroforestry plantation system; AIF, alley/intercrop farming; AMD, agro-multilayer diversity; AWS, agro-woodland system; AOB, agro-organic biodiversity system; AWI, agroforestry-wildlife integration; AIP, agroforestry intensification practice. Perennial cash crops: CF, coffee farm; CFC, coffee under full canopy; CFL, coffee under light shade; CFE, coffee-ecosystem; CFM, coffee mixed system; CFF, coffee full shade forest; CFB, coffee-banana system; CFA, coffee agroforestry; CFG, coffee garden; CCF, cocoa under forest canopy; CDF, cocoa-dominated farm; CBF, cocoa-banana farm; CAW, cocoa-agroforestry with wildlife; CRC, cocoa-rice-crop system; PLA, plantation (general); PLP, perennial plantation; PMA, plantation mixed agriculture; TPP, tropical perennial plantation; PEM, perennial mixed plantation; PFL, plantation forest landscape; OF, organic farming (perennial); ORC, orchards; PCF, perennial crop farm; CCL, cocoa-crop-livestock. Annual cropping: CON, conventional annual cropping; CP, crop production; CG, cereal/grain cultivation; CSG, crop-savanna grassland; CW, cereal/wheat cultivation; INT, intensive agriculture; IIA, intensified irrigated agriculture; CIR, crop irrigation system; CPB, crop production (Brazil); CLF, crop-legume farming; CLC, crop-livestock complex; IDC, intensive dryland cropping; TBI, traditional biodiverse intensive; UAS, upland agricultural system; SHF, smallholder farming; SOP, smallholder organic production; IRA, irrigated rice agriculture; CCP, compound crop production; IOG, irrigated organic garden; OPP, organic production practice; SSI, subsistence smallholder intensive. Silvopastoral: SPS, silvopastoral system; TSP, tropical silvopastoral; SGP, savanna grazing-pastoral; SHP, sheep/pastoral system; SL, silvopastoral landscape; DLS, dryland livestock system; LPB, livestock pasture (Brazil); SFT, silvopastoral farm traditional; TGS, tropical grassland-silvopastoral; SIF, silvopastoral integrated farming; LMO, livestock mixed operations. Mixed farming: MF, mixed farming; MFA, mixed farming-agroforestry; MA, mixed agriculture; MCB, mixed crop biodiversity; MB, mixed biodiversity system; MCF, mixed crop-forest; GM, grassland management mixed; IAV, integrated agri-vegetable; LAV, landscape agri-variety; LI, livestock integration; TF, traditional farming; CL, crop-livestock system; MCE, mixed crop-environment; MCS, mixed crop system; CBS, community-based system. Forest mosaic: FOR, forest; RF, rainforest; TRF, tropical rainforest; PRF, primary rainforest; TFI, traditional forest-influenced farming; ELF, edge/landscape farming; CNF, crop-natural forest mosaic; CRF, crop-rainforest mosaic; FF, forest farm; ICF, integrated crop-forest; TOF, tree-oriented farming; RFA, rainforest agriculture; EBG, edge-border grassland mosaic; TIH, traditional intensified horticulture; TTR, tropical tree restoration. Grassland/rangeland: GL, grassland; GGL, grazed grassland; GLS, grassland system; LG, livestock grazing; LIG, livestock integrated grassland; SAR, savanna/arid rangeland; SSA, sub-Saharan savanna agriculture; NG, natural grassland; SA, savanna agriculture; TOG, tree-over grassland; BK, bushland/savanna kraal; PGV, pasture/grassland variety; SCA, savanna crop agriculture. Wetland/aquatic: RPW, rice paddy wetland; EWP, estuarine wetland pasture; WMR, wetland managed rice; WRC, wetland rice cropping; AQS, aquaculture system; DSR, direct seeded rice; IA, irrigated agriculture; FISH, fishery; RBS, rice-based system.
(TIF)

S17 Fig This is the distribution of violin plots of standardized economic values reported in various monetary units (USD/ha, USD/household/year, USD, etc.) for the identified system categories. Boxplot centers indicate medians, and colors denote system categories. A Kruskal-Wallis test on the within-unit standardized values indicates no significant difference in ecosystem service value across agricultural system types (H = 6.00, df = 7, p = 0.539; η^2^ = 0.000; 127 studies in 8 categories). The same result was obtained within the physically homogeneous per-area family (H = 6.09, df = 4, p = 0.193; η^2^ = 0.053; n = 44). The measurement unit family was strongly associated with the reported value (H = 62.32, df = 3, p < 0.001; η^2^ = 0.42), and the units were unevenly distributed across system categories (chi-square Monte Carlo p = 0.026, Cramer’s V = 0.28).
(TIF)

S18 Fig This is the meta-analysis forest plot and funnel plot for ecosystem service valuation effect sizes. (A) A forest plot displays effect sizes (x-axis) and 95% confidence intervals (horizontal lines) for individual studies (y-axis), organised by year and geographic region. Each point represents a study’s effect size, with green points indicating a positive direction and red points indicating a negative direction. The vertical dashed line at zero represents no effect. (B) A funnel plot assesses publication bias by plotting effect sizes (x-axis) against precision, measured as 1/SE (y-axis, log scale). Green points represent studies with positive effects; red points represent studies with negative effects. The vertical dashed line indicates the overall mean effect size.
(TIF)

S19 Fig This is the distribution of strengths and strength categories across ecosystem service valuation methods. The stacked bar chart displays the percentage distribution of four strength categories (S1-S4) and four weakness categories (W1-W4) for each valuation method (x-axis). Each bar represents 100% coded statements for a given valuation method, with colored segments indicating the proportion of each category: S1: Empirical robustness, S2: Comprehensiveness, S3: Policy relevance, S4: Practical feasibility, W1: Data scarcity, W2: Context sensitivity, W3: Bias risk, and W4: Coverage gaps.
(TIF)

S1 Appendix Codebook. Definitions of valuation methods, ecosystem-service categories, units, and standardization rules used for data coding.
(DOCX)

S2 Appendix PRISMA checklist. Completed PRISMA checklist for the systematic review.
(DOCX)

S3 Appendix List of included studies. Full list of the 146 studies included in the systematic review and meta-analysis.
(DOCX)

S4 Appendix Risk of bias assessment. Risk of bias assessment of the included studies using the adapted ECOBIAS checklist.
(XLSX)

S5 Appendix Meta-regression report. Meta-regression report with variance inflation factors (VIF) and Akaike information criterion (AIC).
(DOCX)
