# Supplementary material for: Economic valuation of ecosystem services in agriculture and agroforestry: A meta-analysis
Source: PLoS One. 2026 Oct 16;21(10):e0360792. doi: 10.1371/journal.pone.0360792 (PMC13674745; doi:10.1371/journal.pone.0360792)
Supplement: Supplementary file 1 [file pone.0360792.s020.docx]

**Codebook for economic valuation of ecosystem services in agriculture and agroforestry: A metaanalysis**

# **1. Purpose and scope**

This systematic review and meta-analysis followed a predefined codebook to guide the economic valuation of ecosystem services (ES) in agriculture and agroforestry. The codebook was used to standardize study selection, data extraction, and analytical procedures across reviewers. All reviewers applied the codebook consistently to reduce interpretation bias and support reproducibility of the results.

# **2. Team and roles**

The review team comprised five researchers with predefined methodological roles. CPK served as Lead Reviewer (R1) and oversaw coordination, supervision, final disagreement resolution, and analysis. MMO served as Secondary Reviewer (R2) and conducted independent screening and data extraction, contributed to the analysis, and participated in disagreement resolution. AS served as Third Reviewer (R3) and acted as the primary arbiter in disagreements between R1 and R2 while validating a sub-sample to assess consistency. TEE and VP served as Additional Reviewers (R4 and R5) and were consulted in cases of persistent disagreement among R1, R2, and R3; they also provided thematic and methodological expertise for complex cases and participated in final decision validation.

All reviewers completed their assigned tasks independently before any discussion and relied exclusively on the guidelines specified in the codebook. A pilot sample of 20 studies was randomly selected to assess R2’s ability to conduct screening and data extraction under the supervision of the other reviewers.

# **3. Study selection process (Screening)**

The selection process follows PRISMA guidelines and is divided into three steps:

### **3.1. Step 1: duplicate removal**

The references imported from the databases were managed sequentially using EndNote, JabRef, and Excel to enable the manual removal of remaining duplicates, which was performed by MMO under the supervision of the other reviewers.

### **3.2. Step 2: Title and abstract screening**

From the first 20 studies, titles and abstracts of all remaining records were screened independently by two reviewers (R1 and R2). Eligibility was assessed against the predefined inclusion and exclusion criteria reported in Table 2. Records judged to be clearly irrelevant were excluded at this stage, whereas records for which eligibility remained uncertain were retained for full-text assessment pending validation by additional reviewers. After that, R2 continued the process under the supervision of the other reviewers.

### **3.3. Step 3: Full-text screening**

After stage 2, the retained studies underwent full-text review by R2 and were then presented to and independently verified by R1. The inclusion and exclusion criteria were subsequently reapplied with greater precision, in accordance with the recommendations of R1, R3, R4, and R5.

### **3.5. Disagreement resolution**

At each screening stage (title/abstract and full-text review), disagreements between R1 and R2 were identified. R1 and R2 discussed the studies under dispute to reach a consensus. When consensus could not be achieved, R3 reviewed the study and made the final decision. In cases of persistent disagreement or particular complexity, R4 (TEE) and R5 (VP) were consulted for final arbitration.

# **4. Data extraction**

### **4.1. Independence of extraction**

Two reviewers independently extracted data from all included studies using the Excel-based extraction form described in Section 5, while consulting each other's assessments.

### **4.2. Resolution of extraction disagreements**

Data were extracted by R2 with input from R1. Discrepancies were discussed between R1 and R2 until consensus was reached. If consensus was not reached, R3 reviewed the data and made the final decision. For the most complex cases, R4 and R5 were consulted for arbitration.

# 5. **Data extraction form**

Table 1. Characteristics of extracted data

| **Data category** | **Variable** | **Definition and extraction instructions** | **Format** |
| --- | --- | --- | --- |
| **Study identification** | Study_id | Unique identification number assigned to each study. | Numeric |
|  | Author(s) year | Name of the first author, followed by “et al.” If more than two authors. | Text |
|  | Publication_year | Year the study was published. | Numeric |
|  | Study_type | Nature of the study. A study may fall under several combined types (e.g. “case study and modeling”). Categories observed in the coded corpus: Empirical research (majority), case study, economic valuation framework, field experiment, modeling, methodological development, meta-analysis, spatial analysis, comparative analysis, simulation-based analysis, survey-based, choice modelling, etc. | Drop-down list (multiple choice) |
| **Geographic context** | Country | Country where the study was conducted (free text). 70 distinct countries recorded to date (e.g. China, Spain, United States, Italy, Indonesia, Ethiopia…). | Text |
|  | Continent | Continent corresponding to the country. | Drop-down list: Africa, Asia, Europe, North America, South America, Oceania |
| **Ecosystem services (es)** | Specific_es | Specific name(s) of the ecosystem service(s) valued, as described by the authors (e.g. Carbon sequestration, pollination, water regulation). | Text |
|  | Es_category | Classification according to the MEA typology (see section 6): Provisioning (prov), regulating (reg), cultural (cult), supporting (sup). A study has been linked to several categories. | Drop-down list (multiple choice) |
|  | Goods_and_services_concerned | Concrete goods or products associated with the valued es (e.g. Rice, timber, irrigation water, honey). | Text |
| **Valuation methods** | Valuation_method | Economic methods used, coded using the corresponding abbreviation (see section 7). | Drop-down list (abbreviation, multiple choice) |
|  | Valuation_approach | Broad methodological family to which the valuation belongs. Values observed in the coded corpus: Value-based approach (large majority), scoring-based approach, integrated modelling approach. | Drop-down list |
|  | Method_rationale | Reason(s) given by the authors for choosing the method (if mentioned). | Text |
| **Economic values** | Original_value | Economic value(s) as reported in the study, with its component (service, method, or scenario) when several values coexist within the same study. | Numeric / structured text |
|  | Original_unit | Original unit of measurement, transcribed as reported (e.g. Cny/ha, yuan/year, usd/m³, eur/t, inr/household/year…). | Text |
|  | Standardized_value_usd | Value converted to USD (see section 8). | Numeric |
|  | Standardized_unit | Standardized unit of measurement retained (see section 8.2). | Drop-down list: USD/ha, USD, USDyear, USD/ha/year, USD/month,  USD/person/year |
| **Agricultural system** | Agricultural_system | Description of the agricultural system as reported by the authors (verbatim extraction), then coded post hoc using a standardized abbreviation. Over 130 distinct abbreviations recorded to date (e.g. AF = agroforestry, CF = conventional farming, OF = organic farming). Full list in Appendix A of the Codebook | Text, coded post hoc |
|  | Specific_practice | Detail of the practice (e.g., silvoarable, maize monoculture, extensive grazing). | Text |
| **Strengths and weaknesses** | Method_strengths | Advantages or strong points of the method used (if mentioned in studies). | Text |
|  | Method_weaknesses | Limitations or drawbacks specific to the valuation method used (if mentioned). | Text |
| **Results and discussion** | Comparison_with_prior_studies | Explicit comparison of results with other studies (if mentioned). | Text |
|  | Key_findings | Main results or findings reported by the authors. | Text |
|  | Recommendations | Recommendations made by the authors. | Text |
|  | Study_limitations | General limitations of the study, distinct from limitations specific to the valuation method (see Method_weaknesses). | Text |
|  | Future_research | Future research directions suggested by the authors. | Text |

# **6. Harmonized classification of ecosystem services**

The classification of ecosystem services follows the Millennium Ecosystem Assessment (MEA) typology, adapted for agricultural and agroforestry contexts.

Table 2. Ecosystem service classification

| **MEA category** | **Sub-category** | **Relevant examples in agriculture/agroforestry** |
| --- | --- | --- |
| **Provisioning services (PROV)** | Food | Crops, livestock, non-timber forest products (fruit, honey), fish. |
|  | Fresh water | Water supply for irrigation, livestock watering. |
|  | Raw materials | Firewood, timber, fibers (cotton, hemp), biomass. |
|  | Genetic resources | Crop varieties, animal breeds, resources for breeding/selection. |
|  | Medicine | Medicinal plants. |
| **Regulating services (REG)** | Climate regulation | Carbon sequestration, regulation of greenhouse gases. |
|  | Water regulation | Flood regulation, groundwater recharge, water filtration. |
|  | Disease regulation | Control of pests and diseases by beneficial organisms. |
|  | Pollination | Pollination of crops by insects. |
|  | Erosion regulation | Soil retention, reduction of wind and water erosion. |
|  | Waste treatment | Decomposition of organic matter, water purification. |
| **Cultural services (CULT)** | Aesthetic values | Attractive agricultural and agroforestry landscapes. |
|  | Recreation and ecotourism | Hunting, fishing, hiking, agritourism. |
|  | Spiritual and religious values | Sacred sites, traditional practices linked to nature. |
|  | Cultural heritage | Traditional farming systems, local knowledge. |
|  | Education and inspiration | Learning opportunities and artistic inspiration. |
| **Supporting services (SUP)** | Soil formation | Pedogenesis, maintenance of soil fertility. |
|  | Nutrient cycling | Nitrogen, phosphorus, and potassium cycles. |
|  | Primary production | Photosynthesis, biomass production. |
|  | Habitat | Provision of habitat for biodiversity. |

# **7. Typology of economic valuation methods**

### **7.1. Revealed preference (RP)-based methods**

| **Code** | **Specific method** | **Description** |
| --- | --- | --- |
| MPV | Market price valuation | Uses observed market prices of goods and services associated with ecosystem service flows. |
| OPG | Observed prices for goods | Values ecosystem-related goods using directly observed transaction prices. |
| TCM | Travel cost method | Infers recreational value from travel expenditures and time costs incurred by visitors. |
| HPM | Hedonic pricing method | Estimates the implicit value of ecosystem-service-related attributes from variation in market prices of related goods. |
| RCA | Replacement cost approach | Values an ecosystem service by the cost of an equivalent human-made substitute. |
| ACM | Avoided cost method | Values a service by the costs avoided because the ecosystem function is present. |
| PFA | Production function approach | Treats the ecosystem service as an input in a production process to estimate its contribution to output. |
| PCM | Productivity change method | Measures value through changes in productivity attributable to the ecosystem service. |
| PL | Productivity loss | Estimates economic losses caused by degradation or loss of the ecosystem service. |
| GMA | Gross margin analysis | Compares gross margins between systems with and without the service-related practice. |
| SP | Shadow pricing | Assigns an implicit price to a non-market service using opportunity-cost or scarcity-based reasoning. |
| RP | Revealed preference | Umbrella term for methods inferring value from observed behavior rather than stated preferences. |
| RES | Residual approach | Derives value as the remaining return after accounting for other inputs. |
| EFAM | Equivalent factor accounting method | Converts ecosystem services into equivalent monetary units using literature-based conversion factors. |
| DRM | Dependence ratio method | Estimates value from the degree to which production depends on the ecosystem service. |

### **7.2. Stated preference (SP)-based methods**

| **Code** | **Specific method** | **Description** |
| --- | --- | --- |
| CVM | Contingent valuation method | Elicits respondents’ willingness to pay or willingness to accept for a change in ecosystem service provision. |
| CE | Choice experiment | Presents respondents with alternative bundles of ecosystem-service attributes and costs, and asks them to choose the preferred option. |
| DBDC | Double-bounded dichotomous choice | A contingent valuation variant that asks two successive yes/no questions to improve precision of WTP or WTA estimates. |
| LSA | Life satisfaction approach | Infers an implicit monetary value by relating changes in ecosystem services to self-reported subjective well-being. |
| EXP | Expert scoring | Estimates value from expert judgment when beneficiary-based valuation data are unavailable. |

### **7.3. Benefit transfer method**

| **Code** | **Specific Method** | **Description** |
| --- | --- | --- |
| BTM | Benefit transfer method | Transfers or adapts ecosystem service value estimates from existing studies at a source site to a policy or study site with similar characteristics |

### **7.4. Other methods (Modeling, integrative frameworks, economic and biophysical analyses)**

| **Code** | **Specific method** | **Description** |
| --- | --- | --- |
| CBA | Cost-benefit analysis | Compares the monetized costs and benefits of a project or practice, including ecosystem service effects. |
| NPV | Net present value | Discounts future costs and benefits associated with ecosystem services to present value. |
| TEV | Total economic value | Integrates use values and non-use values in a single conceptual framework. |
| BEA | Bio-economic approach | Combines biophysical and economic models to evaluate ecosystem services. |
| IBEM | Integrated biophysical-economic model | Explicitly links biophysical processes with economic parameters in an integrated model. |
| LCIA | Life cycle impact assessment | Assesses potential environmental impacts of a product, service, or system across its life cycle. |
| LCC | Life cycle costing | Evaluates the total costs of a product or system across its life cycle. |
| NPP | Net primary productivity approach | Values ecosystem services based on their contribution to net primary productivity. |
| EM | Environmental modeling | Uses environmental or biophysical models to estimate ecosystem service quantities before valuation. |
| MMP | Markov model-based prediction | Uses Markov chains to predict land-use change and associated ecosystem service values. |
| SITE | Spatial modeling (site model) | Uses spatially explicit modeling to estimate the distribution of ecosystem service values. |
| SM | Structural model | Uses a system of interrelated economic equations to estimate ecosystem service value. |
| MA | Modeling approaches | Umbrella category for modeling methods not classified under a more specific code. |
| EAM | Economic assessment model | Combines multiple monetary indicators to evaluate ecosystem services. |
| EDA | Economic decision analysis | Applies decision-analysis, often multi-criteria, to incorporate ecosystem service value. |
| PEA | Partial equilibrium analysis | Examines the equilibrium of a specific market affected by ecosystem services, holding other markets constant. |
| ESU | Economic surplus method | Estimates welfare change by measuring consumer and/or producer surplus associated with an ecosystem service. |
| DUVE | Direct-use value estimation | Captures only direct-use benefits and excludes indirect, option, and non-use values. |
| SEV | Simulated exchange value | Estimates value from simulated or hypothetical exchange conditions, approximating market behavior rather than observed prices. |
| MM | Mathematical modeling | Uses formal equations or generic models to estimate ecosystem-service value without necessarily embedding a spatial or bioeconomic framework. |

# **8. Standardization of economic values**

To facilitate cross-study comparison, all economic values were converted to a standardized basis.

### **8.1. Currency conversion**

Monetary values reported in currencies other than USD were converted to a common USD basis to enable cross-study comparison. Conversions were performed during database construction using publicly available exchange-rate sources at that time.

Table 3. Estimated value converted to USD

| **Id** | **Original value** | **Standardized value (USD)** | **Mean value (USD)** | **Notes** | **Orig_unit_type** | **Converted_unit** | **Currency_origin** |
| --- | --- | --- | --- | --- | --- | --- | --- |
| 1 | ~10.77 × 10³ RMB/ha | ~1,657/ha | 1657 | 1 USD ≈ 6.5 CNY | USD/ha | USD/ha | USD |
| 1 | ~7,652 RMB/ha | ~1,177/ha | 1177 |  | CNY/ha | USD/ha | 1 USD ≈ 6.5 CNY |
| 1 | ~4,286.65-4,618.20 RMB/ha | ~659-711/ha | 685 | Mean of range | CNY/ha | USD/ha | 1 USD ≈ 6.5 CNY |
| 1 | ~13.61-14.32 × 10³ RMB/ha | ~2,094-2,203/ha | 2149 | Mean of range | CNY/ha | USD/ha | 1 USD ≈ 6.5 CNY |
| 2 | 24.2 to 27.9 million Yuan/year | 3.72-4.29 million/year | 4010000 | Mean of range | Yuan/year | USD/year | 1 USD ≈ 6.5 CNY |
| 2 | 31.7 to 42.6 million Yuan/year | 4.88-6.55 million/year | 5720000 | Mean of range | Yuan/year | USD/year | 1 USD ≈ 6.5 CNY |
| 2 | 2.2 to 1.5 million Yuan/year | 0.34-0.23 million/year | 285000 | Mean of range | Yuan/year | USD/year | 1 USD ≈ 6.5 CNY |
| 2 | ~0.057 to 0.015 million Yuan/year | ~0.0088-0.0023 million/year | 5550 | Mean of range | Yuan/year | USD/year | 1 USD ≈ 6.5 CNY |
| 2 | 0.464 to 0.156 million Yuan/year | 0.071-0.024 million/year | 47500 | Mean of range | Yuan/year | USD/year | 1 USD ≈ 6.5 CNY |
| 3 | $2.05 (CEM) to $2.42 (CVM) |  | 2.24 | Mean of range | USD | USD | USD |
| 3 | $82,194.09 (CEM) to $97,107.36 (CVM) |  | 89651 | Mean of range | USD | USD | USD |
| 4 | 0.13 USD per cubic meter |  | 0.13 |  | USD/m³ | USD/m³ | USD |
| 4 | 0.01 USD per cubic meter |  | 0.01 |  | USD/m³ | USD/m³ | USD |
| 5 | Rs. 4666 per household per year | ~62.21/household/year | 62.21 | 1 USD ≈ 75 INR | INR/household/year | USD/household/year | 1 USD ≈ 75 INR |
| 6 | $1-2 billion gain |  | 1500000000 | Mean of range | USD | USD | USD |
| 7 | $2,645 per hectare per year |  | 2645 |  | USD/ha/year | USD/ha/year | USD |
| 7 | $1,634 per hectare per year |  | 1634 |  | USD/ha/year | USD/ha/year | USD |
| 7 | $54,782 per hectare |  | 1370 | Annualized | USD/ha | USD/ha | USD |
| 8 | EUR113.23 | USD 133.18 | 133.18 | 1 USD ≈ 0.85 EUR | EUR | USD | 1 USD ≈ 0.85 EUR |
| 9 | 1,100 |  | 1100 |  | USD | USD | USD |
| 9 | 120 |  | 120 |  | USD | USD | USD |
| 9 | 150 |  | 150 |  | USD | USD | USD |
| 9 | 120 |  | 120 |  | USD | USD | USD |
| 9 | 30 |  | 30 |  | USD | USD | USD |
| 9 | 2,800 |  | 2800 |  | USD | USD | USD |
| 9 | 1,200 |  | 1200 |  | USD | USD | USD |
| 9 | 180 |  | 180 |  | USD | USD | USD |
| 9 | 160 |  | 160 |  | USD | USD | USD |
| 9 | 130 |  | 130 |  | USD | USD | USD |
| 9 | 30 |  | 30 |  | USD | USD | USD |
| 9 | 1,900 |  | 1900 |  | USD | USD | USD |
| 9 | 1,000 |  | 1000 |  | USD | USD | USD |
| 9 | 110 |  | 110 |  | USD | USD | USD |
| 9 | 120 |  | 120 |  | USD | USD | USD |
| 9 | 100 |  | 100 |  | USD | USD | USD |
| 9 | 30 |  | 30 |  | USD | USD | USD |
| 9 | 3,000 |  | 3000 |  | USD | USD | USD |
| 9 | 1,300 |  | 1300 |  | USD | USD | USD |
| 9 | 200 |  | 200 |  | USD | USD | USD |
| 9 | 170 |  | 170 |  | USD | USD | USD |
| 9 | 140 |  | 140 |  | USD | USD | USD |
| 9 | 30 |  | 30 |  | USD | USD | USD |
| 10 | 0.43 € t⁻¹ | 0.51/t | 0.51 | 1 USD ≈ 0.85 EUR | EUR/t | USD/t | 1 USD ≈ 0.85 EUR |
| 10 | 802.6 € t⁻¹ | 944/t | 944 | 1 USD ≈ 0.85 EUR | EUR/t | USD/t | 1 USD ≈ 0.85 EUR |
| 11 | -0.0827 €/m² | -0.097/m² | -0.097 |  | EUR/m² | USD/m² | 1 USD ≈ 0.85 EUR |
| 12 | $1,488 |  | 1488 |  | USD | USD | USD |
| 12 | $6,171 |  | 1234 | Annualized | USD | USD | USD |
| 13 | JPY 3.8 billion | 34.5 million | 34500000 | 1 USD ≈ 110 JPY | JPY/year | USD/year | 1 USD ≈ 110 JPY |
| 13 | JPY 11.7 billion | 106.4 million | 106400000 |  | JPY/year | USD/year | 1 USD ≈ 110 JPY |
| 14 | $188/household |  | 188 |  | USD/household | USD/household | USD |
| 14 | $5.12-6.26/month | 5.69 | 5.69 |  | USD/month | USD/month | USD |
| 15 | $81.3 million |  | 81300000 |  | USD | USD | USD |
| 15 | $12.8 billion |  | 12800000000 |  | USD | USD | USD |
| 16 | 1.18 Yuan/m³ | 0.18/m³ | 0.18 |  | CNY/m³ | USD/m³ | CNY |
| 16 | 1.07 Yuan/m³ | 0.16/m³ | 0.16 |  | CNY/m³ | USD/m³ | CNY |
| 16 | 0.98 Yuan/m³ | 0.15/m³ | 0.15 |  | CNY/m³ | USD/m³ | CNY |
| 17 | $607,688,596.50 |  | 607688596.5 |  | USD | USD | USD |
| 17 | 11,048,883.57 |  | 11048883.57 |  | USD | USD | USD |
| 18 | €51 billion/year | 60 billion/year | 60000000000 |  | EUR/year | USD/year | EUR |
| 18 | €5.7 billion/year | 6.7 billion/year | 6700000000 |  | EUR/year | USD/year | EUR |
| 19 | NZD 3717/ha/year | 2655/ha/year | 2655 | 1 USD ≈ 1.4 NZD | NZD/ha/year | USD/ha/year | NZD |
| 19 | NZD 1299/ha/year | 928/ha/year | 928 |  | NZD/ha/year | USD/ha/year | NZD |
| 19 | NZD 1921/ha/year | 1372/ha/year | 1372 |  | NZD/ha/year | USD/ha/year | NZD |
| 19 | NZD 2293/ha/year | 1638/ha/year | 1638 |  | NZD/ha/year | USD/ha/year | NZD |
| 19 | NZD 4165/ha/year | 2975/ha/year | 2975 |  | NZD/ha/year | USD/ha/year | NZD |
| 19 | NZD 4548/ha/year | 3249/ha/year | 3249 |  | NZD/ha/year | USD/ha/year | NZD |
| 20 | $327-699 |  | 513 | Mean of range | USD | USD | USD |
| 20 | 6.7-7.1 billion |  | 6.9 | Mean of range | USD | USD | USD |
| 21 | £956 m/year | 1,223.68 million/year | 1223.68 | 1 GBP ≈ 1.28 USD | GBP/year | USD/year | 1 USD ≈ 0.8 GBP |
| 22 | 20.28 USD/10 m³ |  | 2.028 | Per m³ | USD/m³ | USD/m³ | USD |
| 22 | 11.49 USD/10 m³ |  | 1.149 | Per m³ | USD/m³ | USD/m³ | USD |
| 22 | 18.56 USD/10 m³ |  | 1.856 | Per m³ | USD/m³ | USD/m³ | USD |
| 23 | $20.02-46.25 |  | 33.14 | Mean of range | USD | USD | USD |
| 23 | $2.40-5.55 |  | 3.98 | Mean of range | USD | USD | USD |
| 24 | $515/taxpayer/year |  | 515 |  | USD/taxpayer/year | USD/taxpayer/year | USD |
| 24 | $105 billion |  | 105000000000 |  | USD | USD | USD |
| 25 | NT$718.92/person/year | 24/person/year | 24 | 1 USD ≈ 30 NT$ | TWD/person/year | USD/person/year | 1 USD ≈ 30 NT$ |
| 25 | NT$794.32/person/year | 26/person/year | 26 |  | TWD/person/year | USD/person/year | 1 USD ≈ 30 NT$ |
| 26 | NT$751.9/person/year | 25/person/year | 25 |  | TWD/person/year | USD/person/year | 1 USD ≈ 30 NT$ |
| 26 | NT$536.3/person/year | 18/person/year | 18 |  | TWD/person/year | USD/person/year | 1 USD ≈ 30 NT$ |
| 27 | $460-5,240/ha/yr |  | 2850 | Mean of range | USD/ha/year | USD/ha/year | USD |
| 27 | $50-1,240/ha/yr |  | 645 | Mean of range | USD/ha/year | USD/ha/year | USD |
| 28 | €559/ha/yr | 658/ha/yr | 658 |  | EUR/ha/year | USD/ha/year | 1 USD ≈ 0.85 EUR |
| 28 | €453/ha/yr | 533/ha/yr | 533 |  | EUR/ha/year | USD/ha/year | 1 USD ≈ 0.85 EUR |
| 28 | €141/ha/yr | 166/ha/yr | 166 |  | EUR/ha/year | USD/ha/year | 1 USD ≈ 0.85 EUR |
| 29 | 11,387 SEK/ha | 1,250/ha | 1250 | 1 USD ≈ 9.1 SEK | SEK/ha | USD/ha | 1 USD ≈ 9.1 SEK |
| 29 | 6,398 SEK/ha | 703/ha | 703 |  | SEK/ha | USD/ha | 1 USD ≈ 9.1 SEK |
| 30 | 10-15 THB/tonne | 0.28-0.42/tonne | 0.35 | 1 USD ≈ 36 THB | THB/tonne | USD/tonne | 1 USD ≈ 36 THB |
| 31 | 225.56 USD/ha/yr |  | 225.56 |  | USD/ha/year | USD/ha/year | USD |
| 31 | 986.69 USD/ha/yr |  | 986.69 |  | USD/ha/year | USD/ha/year | USD |
| 32 | 150.4B CNY | 23.14B | 23140000000 |  | CNY | USD | 1 USD ≈ 6.5 CNY |
| 32 | 157.28B CNY | 24.20B | 24200000000 |  | CNY | USD | 1 USD ≈ 6.5 CNY |
| 33 | $1,238-1,688/ha/yr |  | 1463 | Mean of range | USD/ha/year | USD/ha/year | USD |
| 33 | $1,052-1,234/ha/yr |  | 1143 | Mean of range | USD/ha/year | USD/ha/year | USD |
| 34 | 3,613,651 €/year | 4,251,354/year | 4251354 |  | EUR/year | USD/year | 1 USD ≈ 0.85 EUR |
| 35 | 67,956 yuan/ha/year | 10,455/ha/year | 10455 |  | CNY/ha/year | USD/ha/year | 1 USD ≈ 6.5 CNY |
| 36 | $0.32-0.58/kg |  | 0.45 | Mean of range | USD/kg | USD/kg | USD |
| 37 | 669.2B-1.4545T won | 0.56-1.21B | 885000000 | 1 USD ≈ 1,200 KRW | KRW | USD | 1 USD ≈ 1200 KRW |
| 38 | 10,820 USD/ha |  | 10820 |  | USD/ha | USD/ha | USD |
| 38 | >10,000 USD/ha |  | 10000 | Lower bound | USD/ha | USD/ha | USD |
| 38 | <2,000 USD/ha |  | 2000 | Upper bound | USD/ha | USD/ha | USD |
| 38 | 14,700 USD/ha |  | 14700 |  | USD/ha | USD/ha | USD |
| 38 | >15,000 USD/ha |  | 15000 | Lower bound | USD/ha | USD/ha | USD |
| 39 | €6,800/ha | 8,000/ha | 8000 | 1 USD ≈ 0.85 EUR | EUR/ha | USD/ha | 1 USD ≈ 0.85 EUR |
| 39 | €4,200/ha | 4,941/ha | 4941 |  | EUR/ha | USD/ha | 1 USD ≈ 0.85 EUR |
| 39 | €5,600/ha | 6,588/ha | 6588 |  | EUR/ha | USD/ha | 1 USD ≈ 0.85 EUR |
| 40 | €12.08/year | 14.21/year | 14.21 |  | EUR/year | USD/year | 1 USD ≈ 0.85 EUR |
| 40 | €12.63/year | 14.86/year | 14.86 |  | EUR/year | USD/year | 1 USD ≈ 0.85 EUR |
| 40 | €53.16/year | 62.54/year | 62.54 |  | EUR/year | USD/year | 1 USD ≈ 0.85 EUR |
| 40 | €30.85/year | 36.29/year | 36.29 |  | EUR/year | USD/year | 1 USD ≈ 0.85 EUR |
| 41 | 210-354 Mg DM/ha | 210-354 Mg DM/ha | 282 | Physical units only | Mg DM/ha | Mg DM/ha |  |
| 41 | 28-47 Mg DM/ha | 28-47 Mg DM/ha | 37.5 |  | Mg DM/ha | Mg DM/ha |  |
| 42 | IDR 17,000/month | 1.05/month | 1.05 | 1 USD ≈ 17,000 IDR | IDR/month | USD/month | 1 USD ≈ 17 IDR |
| 43 | IDR 19,100,000/ha/yr | 1,124/ha/yr | 1124 |  | IDR/ha/year | USD/ha/year | 1 USD ≈ 17 IDR |
| 43 | IDR 15,200,000/ha/yr | 894/ha/yr | 894 |  | IDR/ha/year | USD/ha/year | 1 USD ≈ 17 IDR |
| 44 | $3.20/kg |  | 3.2 |  | USD/kg | USD/kg | USD |
| 44 | $2.84/kg |  | 2.84 |  | USD/kg | USD/kg | USD |
| 45 | -$275 million |  | -275000000 |  | USD | USD | USD |
| 45 | -$116 million |  | -116000000 |  | USD | USD | USD |
| 46 | JPY 953.5 | 8.67/visit | 8.67 | 1 USD ≈ 110 JPY | JPY/visit | USD/visit | 1 USD ≈ 110 JPY |
| 47 | €1,049/ha/yr | 1,234/ha/yr | 1234 |  | EUR/ha/year | USD/ha/year | 1 USD ≈ 0.85 EUR |
| 47 | €1,590/ha/yr | 1,871/ha/yr | 1871 |  | EUR/ha/year | USD/ha/year | 1 USD ≈ 0.85 EUR |
| 48 | R300-R3600/ha/yr | 20-240/ha/yr | 130 | 1 USD ≈ 15 ZAR | ZAR/ha/year | USD/ha/year | 1 USD ≈ 15 ZAR |
| 49 | 1.5-6.7 THB/m³ | 0.04-0.19/m³ | 0.115 | 1 USD ≈ 36 THB | THB/m³ | USD/m³ | 1 USD ≈ 36 THB |
| 50 | -86,000 USD/ha |  | -86000 |  | USD/ha | USD/ha | USD |
| 50 | 5,300 USD/ha |  | 5300 |  | USD/ha | USD/ha | USD |
| 51 | 2150 €/ha | 2529/ha | 2529 | 1 USD ≈ 0.85 EUR | EUR/ha | USD/ha | 1 USD ≈ 0.85 EUR |
| 51 | 104.1-260.2 €/ha/yr | 122-306/ha/yr | 214 | Mean of range | EUR/ha/year | USD/ha/year | 1 USD ≈ 0.85 EUR |
| 52 | 1200-3000 Int$/ha/yr |  | 2100 | Mean of range | IntUSD/ha/year | IntUSD/ha/year | USD |
| 52 | >1000 Int$/ha/yr |  | 1000 | Lower bound | IntUSD/ha/year | IntUSD/ha/year | USD |
| 53 | €1043.06/ha | 1227/ha | 1227 |  | EUR/ha | USD/ha | 1 USD ≈ 0.85 EUR |
| 53 | €1494.51/ha | 1758/ha | 1758 |  | EUR/ha | USD/ha | 1 USD ≈ 0.85 EUR |
| 54 | 466.9 USD/ha |  | 466.9 |  | USD/ha | USD/ha | USD |
| 54 | 436.7 USD/ha |  | 436.7 |  | USD/ha | USD/ha | USD |
| 55 | AUD 85,524,000 | 61,088,571 | 61088571 | 1 USD ≈ 1.4 AUD | AUD | USD | 1 USD ≈ 1.4 AUD |
| 56 | €525.3M/year | 618M/year | 618000000 |  | EUR/year | USD/year | 1 USD ≈ 0.85 EUR |
| 57 | IDR 11.78B/year | 693,000/year | 693000 |  | IDR/year | USD/year | 1 USD ≈ 17 IDR |
| 58 | IDR 1.083T/year | 63.7M/year | 63700000 |  | IDR/year | USD/year | 1 USD ≈ 17 IDR |
| 59 | 5.4 €/household/month | 6.35/month | 6.35 |  | EUR/month | USD/month | 1 USD ≈ 0.85 EUR |
| 60 | Rp 61.02B/year | 3.59M/year | 3590000 |  | IDR/year | USD/year | 1 USD ≈ 17 IDR |
| 61 | CHF 410/year | 453/year | 453 | 1 USD ≈ 0.9 CHF | CHF/year | USD/year | 1 USD ≈ 0.9 CHF |
| 62 | $108,000-197,000/km² |  | 152500 | Mean of range | USD/km² | USD/km² |  |
| 63 | Rs 1,503-32,672/ha | 20-436/ha | 228 | 1 USD ≈ 75 INR | INR/ha | USD/ha | 1 USD ≈ 75 INR |
| 64 | 91969.59 |  | 91969.59 |  | USD | USD | USD |
| 65 | IDR 27,333/visit | 1.61/visit | 1.61 |  | IDR/visit | USD/visit | 1 USD ≈ 17 IDR |
| 66 | $18,674.8-56,966.4 |  | 37820.6 | Mean of range | USD | USD | USD |
| 67 | €3.8B/year | 4.47B/year | 4470000000 |  | EUR/year | USD/year | 1 USD ≈ 0.85 EUR |
| 68 | $3,174/household |  | 3174 |  | USD/household | USD/household | USD |
| 69 | €352/ha | 414/ha | 414 |  | EUR/ha | USD/ha | 1 USD ≈ 0.85 EUR |
| 70 | 136,664 MAD | 13,666/ha | 13666 | 1 USD ≈ 10 MAD | MAD/ha | USD/ha | 1 USD ≈ 10 MAD |
| 71 | $22,490-106,300/ha |  | 64395 | Mean of range | USD/ha | USD/ha | USD |
| 72 | $7,710-10,650/ha |  | 9180 | Mean of range | USD/ha | USD/ha | USD |
| 73 | 455,058.88 ¥/ha | 70,009/ha | 70009 | 1 USD ≈ 6.5 CNY | CNY/ha | USD/ha | 1 USD ≈ 6.5 CNY |
| 74 | $3,300/ha |  | 3300 |  | USD/ha | USD/ha | USD |
| 75 | €31-44/year | 36-52/year | 44 | Mean of range | EUR/year | USD/year | 1 USD ≈ 0.85 EUR |
| 76 | 1191 yuan/greenhouse | 183/greenhouse | 183 |  | CNY/greenhouse | USD/greenhouse | 1 USD ≈ 6.5 CNY |
| 77 | 180.264B RMB | 27.73B | 27730000000 |  | CNY | USD | 1 USD ≈ 6.5 CNY |
| 78 | $108,600/ha |  | 108600 |  | USD/ha | USD/ha | None |
| 79 | NT$17.757B | 592M | 592000000 | 1 USD ≈ 30 NT$ | TWD | USD | 1 USD ≈ 30 NT$ |
| 80 | $0.551B |  | 551000000 |  | USD | USD | USD |
| 81 | 352,219.4×10⁴ to 767,061.5×10⁴ yuan | 54.1M to 118M | 86.5 | 1 USD ≈ 6.5 CNY | CNY | USD | 1 USD ≈ 6.5 CNY |
| 82 | Rs. 48/visit | 0.64/visit | 0.64 |  | INR/visit | USD/visit | 1 USD ≈ 75 INR |
| 83 | 50,270 EUR/100ha | 59,141/100ha | 59141 |  | EUR/100ha | USD/100ha | 1 USD ≈ 0.85 EUR |
| 84 | RMB 6159.60/ha | 948/ha | 948 |  | CNY/ha | USD/ha | 1 USD ≈ 6.5 CNY |
| 85 | €113.9M | 134M | 134000000 |  | EUR | USD | 1 USD ≈ 0.85 EUR |
| 86 | 11.72T NT | 391B | 391000000000 |  | TWD | USD | 1 USD ≈ 30 NT$ |
| 87 | 46 €/ha | 54/ha | 54 |  | EUR/ha | USD/ha | 1 USD ≈ 0.85 EUR |
| 88 | 4.11 DA/m² | 0.037/m² | 0.037 | 1 USD ≈ 110 DZD | DZD/m² | USD/m² | 1 USD ≈ 110 DZD |
| 89 | 98.02 RMB/month | 15.08/month | 15.08 |  | CNY/month | USD/month | 1 USD ≈ 6.5 CNY |
| 90 | $12-48/ha |  | 30 | Mean of range | USD/ha | USD/ha | USD |
| 91 | A$40-66/tCO₂-e | 29-47/t | 38 | 1 USD ≈ 1.4 AUD | AUD/t | USD/t | 1 USD ≈ 1.4 AUD |
| 92 | 237 CNY mu⁻¹ yr⁻¹ | 36/ha | 36 | 1 mu ≈ 0.0667 ha | CNY/mu/year | USD/ha/year | 1 USD ≈ 6.5 CNY |
| 93 | €105.75-136.88 |  | 121.32 | Mean of range | EUR | USD | 1 USD ≈ 0.85 EUR |
| 94 | $863M-1.015B |  | 939000000 | Mean of range | USD | USD | USD |
| 95 | £1,371.71M/year | 1.71B/year | 1710000000 | 1 USD ≈ 0.8 GBP | GBP/year | USD/year | 1 USD ≈ 0.8 GBP |
| 96 | Up to R22,426 | 1,495 | 1495 |  | ZAR | USD | 1 USD ≈ 15 ZAR |
| 97 | $686-1,236/ha |  | 961 | Mean of range | USD/ha | USD/ha | USD |
| 98 | Rp831,613-831,633/ha | 49-49/ha | 49 |  | IDR/ha | USD/ha | 1 USD ≈ 17 IRD |
| 99 | CNY 9800.84/ha | 1,508/ha | 1508 |  | CNY/ha | USD/ha | 1 USD ≈ 6.5 CNY |
| 100 | £842M/year | 1.05B/year | 1050000000 |  | GBP/year | USD/year | GBP |
| 101 | 5057.99 × 10⁴ RMB | 7.78M | 7780000 |  | CNY | USD | CNY |
| 102 | 150-5500 EUR/ton/ha/yr | 176-6,471/ton/ha/yr | 3323.5 |  | EUR/ton/ha/year | USD/ton/ha/year | 1 USD ≈ 0.85 EUR |
| 103 | $1,323/ha |  | 1323 |  | USD/ha | USD/ha | USD |
| 104 | 275.64 ETB/ha | 7.9/ha | 7.9 | 1 USD ≈ 35 ETB | ETB/ha | USD/ha | 1 USD ≈ 35 ETB |
| 105 | €156K-231K | 184K-272K | 228000 |  | EUR | USD | 1 USD ≈ 0.85 EUR |
| 106 | €5083/ha | 5,980/ha | 5980 |  | EUR/ha | USD/ha | 1 USD ≈ 0.85 EUR |
| 107 | 2,026 USD/ha |  | 2026 |  | USD/ha | USD/ha | USD |
| 108 | $3,142/ha |  | 3142 |  | USD/ha | USD/ha | USD |
| 109 | €0.16/kgCO₂e | 0.19/kg | 0.19 |  | EUR/kg | USD/kg | 1 USD ≈ 0.85 EUR |
| 110 | $2,970-7,729/ha |  | 5349.5 |  | USD/ha | USD/ha | USD |
| 111 | 850 €/person | 1,000/person | 1000 |  | EUR/person | USD/person | 1 USD ≈ 0.85 EUR |
| 112 | $6,163-11,546/year |  | 8854.5 |  | USD/year | USD/year | USD |
| 113 | NZD $1.96B | 1.4B | 1400000000 |  | NZD | USD | 1 USD ≈ 1.4 NZD |
| 114 | -$3.74M to $12M |  | 4130000 |  | USD | USD | USD |
| 115 | $68-200/ha/yr |  | 134 |  | USD/ha/year | USD/ha/year | USD |
| 115 | $110-425/ha/yr |  | 267.5 |  | USD/ha/year | USD/ha/year | USD |
| 116 | $1.5-17M/year |  | 9250000 |  | USD/year | USD/year | USD |
| 117 | Ksh 70.05/kg | 0.70/kg | 0.70 |  | KES/kg | USD/kg | 1 USD ≈ 100 KES |
| 118 | 961.68-972.66 birr/year |  | 967.17 | 1 USD ≈ 35 ETB | ETB/year | USD/year | 1 USD ≈ 35 ETB |
| 119 | €121.2-196.8/person |  | 159 |  | EUR/person | USD/person | 1 USD ≈ 0.85 EUR |
| 120 | €159.30/year | 187/year | 187 |  | EUR/year | USD/year | 1 USD ≈ 0.85 EUR |
| 121 | 7,447 Yuan/ha | 1,146/ha | 1146 |  | CNY/ha | USD/ha | 1 USD ≈ 6.5 CNY |
| 122 | $73.3M to $156.7M |  | 115000000 |  | USD | USD | USD |
| 123 | 26.99 yuan/month | 4.15/month | 4.15 |  | CNY/month | USD/month | 1 USD ≈ 6.5 CNY |
| 124 | €0.39-0.74 |  | 0.565 |  | EUR | USD | 1 USD ≈ 0.85 EUR |
| 125 | 3.1B EUR | 3.65B | 3650000000 |  | EUR | USD | 1 USD ≈ 0.85 EUR |
| 126 | 3,646,368 CZK | 167,000 | 167000 | 1 USD ≈ 22 CZK | CZK | USD | 1 USD ≈ 22 CZK |
| 127 | 50,270 EUR/year | 59,141/year | 59141 |  | EUR/year | USD/year | 1 USD ≈ 0.85 EUR |
| 128 | $4,006M/year |  | 4006000000 |  | USD/year | USD/year | USD |
| 129 | €5.13/month | 6.04/month | 6.04 |  | EUR/month | USD/month | 1 USD ≈ 0.85 EUR |
| 130 | CNY 170.76/person | 26.27/person | 26.27 |  | CNY/person | USD/person | 1 USD ≈ 6.5 CNY |
| 131 | $983.2M |  | 983200000 |  | USD | USD | USD |
| 132 | $685/ha/yr |  | 685 |  | USD/ha/year | USD/ha/year | USD |
| 133 | €22.09/year | 26/year | 26 |  | EUR/year | USD/year | 1 USD ≈ 0.85 EUR |
| 134 | $245.2/unit |  | 245.2 |  | USD/unit | USD/unit | USD |
| 135 | 3.62 × 10⁸ RMB | 55.7M | 55700000 |  | CNY | USD | 1 USD ≈ 6.5 CNY |
| 136 | Birr 738-784/ha | 21.06-22.39/ha | 21.73 |  | ETB/ha | USD/ha | 1 USD ≈ 35 ETB |
| 137 | PhP 112,521-431,583/ha/yr | 2,250-8,632/ha/yr | 5441 |  | PHP/ha/year | USD/ha/year | 1 USD ≈ 50 PHP |
| 138 | $36.2-52.5 |  | 43.83 |  | USD | USD | USD |
| 139 | 10% of €1.325B | 156M/year | 156000000 |  | EUR/year | USD/year | 1 USD ≈ 0.85 EUR |
| 140 | 4.57 USD/month |  | 4.57 |  | USD/month | USD/month | USD |
| 141 | Rs 6,70,496/ha | 8,940/ha | 8940 |  | INR/ha | USD/ha | 1 USD ≈ 75 INR |
| 142 | $11.6-19.6B/year |  | 15600000000 |  | USD/year | USD/year | USD |
| 143 | $129M to $147M |  | 138000000 |  | USD | USD | USD |
| 144 | 39.194 USD/HH/year |  | 39.19 |  | USD/household/year | USD/household/year | USD |
| 145 | VND188,300/ha | 8.17/ha | 8.17 | 1 USD ≈ 23,000 VND | VND/ha | USD/ha | 1 USD ≈ 23VND |
| 146 | 30% (Ksh 52.83/kg) | 0.53/kg | 0.53 |  | KES/kg | USD/kg | 1 USD ≈ 100 KES |

### **8.2. Unit standardization**

If values were reported in nonannual time units (e.g., per month), they were annualized. Values reported per nonstandard area units (e.g., per m²) were converted to hectares. Lump-sum amounts (e.g., total USD for a project) were retained as such and converted to USD/ha/year when both area and duration were available; otherwise, they were reported as USD or USD/year. To standardize values before log transformation, the following formula was applied depending on whether the monetary value was expressed as a total amount or per unit: for mean USD values > 0, Log_Value = log10(mean USD); for mean USD values < 0, Log_Value = -log10(|mean USD|). Because log10(a × USD/unit) = log10(a) + log10(USD/unit), this transformation should be interpreted as operating on ratios of magnitudes rather than on units themselves.

Table 4. Standardization of mean value estimates

| **ID** | **Mean value (USD)** | **Log10 Scale** |
| --- | --- | --- |
| 1 | 1 657 | 3.219 |
| 2 | 4 010 000 | 6.603 |
| 3 | 2.24 | 0.350 |
| 4 | 0.130 | -0.886 |
| 5 | 62.21 | 1.794 |
| 6 | 1 500 000 000 | 9.176 |
| 7 | 2 645 | 3.422 |
| 8 | 133.18 | 2.124 |
| 9 | 1 100 | 3.041 |
| 10 | 0.510 | -0.292 |
| 11 | -0.097 | 1.013 |
| 12 | 1 488 | 3.173 |
| 13 | 34 500 000 | 7.538 |
| 14 | 188.00 | 2.274 |
| 15 | 81 300 000 | 7.910 |
| 16 | 0.180 | -0.745 |
| 17 | 607 688 596 | 8.784 |
| 18 | 60 000 000 000 | 10.778 |
| 19 | 2 655 | 3.424 |
| 20 | 513.00 | 2.710 |
| 21 | 1 224 | 3.088 |
| 22 | 2.03 | 0.307 |
| 23 | 33.14 | 1.520 |
| 24 | 515.00 | 2.712 |
| 25 | 24.00 | 1.380 |
| 26 | 25.00 | 1.398 |
| 27 | 2 850 | 3.455 |
| 28 | 658.00 | 2.818 |
| 29 | 1 250 | 3.097 |
| 30 | 0.350 | -0.456 |
| 31 | 225.56 | 2.353 |
| 32 | 23 140 000 000 | 10.364 |
| 33 | 1 463 | 3.165 |
| 34 | 4 251 354 | 6.629 |
| 35 | 10 455 | 4.019 |
| 36 | 0.450 | -0.347 |
| 37 | 885 000 000 | 8.947 |
| 38 | 10 820 | 4.034 |
| 39 | 8 000 | 3.903 |
| 40 | 14.21 | 1.153 |
| 41 | 282.00 | 2.450 |
| 42 | 1.05 | 0.021 |
| 43 | 1 124 | 3.051 |
| 44 | 3.20 | 0.505 |
| 45 | -275 000 000 | -8.439 |
| 46 | 8.67 | 0.938 |
| 47 | 1 234 | 3.091 |
| 48 | 130.00 | 2.114 |
| 49 | 0.115 | -0.939 |
| 50 | -86 000 | -4.934 |
| 51 | 2 529 | 3.403 |
| 52 | 2 100 | 3.322 |
| 53 | 1 227 | 3.089 |
| 54 | 466.90 | 2.669 |
| 55 | 61 088 571 | 7.786 |
| 56 | 618 000 000 | 8.791 |
| 57 | 693 000 | 5.841 |
| 58 | 63 700 000 | 7.804 |
| 59 | 6.35 | 0.803 |
| 60 | 3 590 000 | 6.555 |
| 61 | 453.00 | 2.656 |
| 62 | 152 500 | 5.183 |
| 63 | 228.00 | 2.358 |
| 64 | 91 970 | 4.964 |
| 65 | 1.61 | 0.207 |
| 66 | 37 821 | 4.578 |
| 67 | 4 470 000 000 | 9.650 |
| 68 | 3 174 | 3.502 |
| 69 | 414.00 | 2.617 |
| 70 | 13 666 | 4.136 |
| 71 | 64 395 | 4.809 |
| 72 | 9 180 | 3.963 |
| 73 | 70 009 | 4.845 |
| 74 | 3 300 | 3.519 |
| 75 | 44.00 | 1.643 |
| 76 | 183.00 | 2.262 |
| 77 | 27 730 000 000 | 10.443 |
| 78 | 108 600 | 5.036 |
| 79 | 592 000 000 | 8.772 |
| 80 | 551 000 000 | 8.742 |
| 81 | 86.50 | 1.937 |
| 82 | 0.640 | -0.194 |
| 83 | 59 141 | 4.772 |
| 84 | 948.00 | 2.977 |
| 85 | 134 000 000 | 8.127 |
| 86 | 391 000 000 000 | 11.592 |
| 87 | 54.00 | 1.732 |
| 88 | 0.037 | -1.432 |
| 89 | 15.08 | 1.178 |
| 90 | 30.00 | 1.477 |
| 91 | 38.00 | 1.580 |
| 92 | 36.00 | 1.556 |
| 93 | 121.32 | 2.084 |
| 94 | 939 000 000 | 8.973 |
| 95 | 1 710 000 000 | 9.233 |
| 96 | 1 495 | 3.175 |
| 97 | 961.00 | 2.983 |
| 98 | 49.00 | 1.690 |
| 99 | 1 508 | 3.178 |
| 100 | 1 050 000 000 | 9.021 |
| 101 | 7 780 000 | 6.891 |
| 102 | 3 324 | 3.522 |
| 103 | 1 323 | 3.122 |
| 104 | 7.90 | 0.898 |
| 105 | 228 000 | 5.358 |
| 106 | 5 980 | 3.777 |
| 107 | 2 026 | 3.307 |
| 108 | 3 142 | 3.497 |
| 109 | 0.190 | -0.721 |
| 110 | 5 350 | 3.728 |
| 111 | 1 000 | 3.000 |
| 112 | 8 854 | 3.947 |
| 113 | 1 400 000 000 | 9.146 |
| 114 | 4 130 000 | 6.616 |
| 115 | 134.00 | 2.127 |
| 116 | 9 250 000 | 6.966 |
| 117 | 0.700 | -0.155 |
| 118 | 967.17 | 2.985 |
| 119 | 159.00 | 2.201 |
| 120 | 187.00 | 2.272 |
| 121 | 1 146 | 3.059 |
| 122 | 115 000 000 | 8.061 |
| 123 | 4.15 | 0.618 |
| 124 | 0.565 | -0.248 |
| 125 | 3 650 000 000 | 9.562 |
| 126 | 167 000 | 5.223 |
| 127 | 59 141 | 4.772 |
| 128 | 4 006 000 000 | 9.603 |
| 129 | 6.04 | 0.781 |
| 130 | 26.27 | 1.419 |
| 131 | 983 200 000 | 8.993 |
| 132 | 685.00 | 2.836 |
| 133 | 26.00 | 1.415 |
| 134 | 245.20 | 2.390 |
| 135 | 55 700 000 | 7.746 |
| 136 | 21.73 | 1.337 |
| 137 | 5 441 | 3.736 |
| 138 | 43.83 | 1.642 |
| 139 | 156 000 000 | 8.193 |
| 140 | 4.57 | 0.660 |
| 141 | 8 940 | 3.951 |
| 142 | 15 600 000 000 | 10.193 |
| 143 | 138 000 000 | 8.140 |
| 144 | 39.19 | 1.593 |
| 145 | 8.17 | 0.912 |
| 146 | 0.530 | -0.276 |

# **Appendix A. Most frequent agricultural system acronyms and ecosystem service names**

Table 5. Frequently reported agricultural systems in the reviewed studies

| **Code** | **Agricultural system (standardized label)** |
| --- | --- |
| AA | Agricultural areas |
| AAC | Agroforestry alley cropping |
| AAF | Agroforestry in rural forested areas |
| AF | Agroforestry |
| AFS | Arable farming on small farms |
| AFS2 | Agroforestry systems |
| AG | Allotment gardens |
| AIF | Agrotourism based on integrated farming |
| AIP | Agritourism integrated with farming practices |
| AMD | Agroforestry with Melia dubia plantations |
| AOB | Agroforestry combining olives and barley |
| APF | Agroforestry farms in public coniferous forests |
| APS | Agroforestry with protective forest plantations |
| AQS | Aquatic systems |
| ATF | Agrotourism involving fruit cultivation |
| ATF2 | Agroforestry integrating timber trees with food crops |
| AWI | Agroforestry combining woody perennials with crops and livestock |
| AWS | Agroforestry systems based on wood pastures |
| BK | Beekeeping |
| CAF | Conventional arable farming |
| CAW | Conventional agriculture in arid watersheds |
| CBF | Coconut-based farming systems |
| CBS | Commercial brassica seed production |
| CCF | Conventional coffee farming |
| CCL | Conventional crop and livestock farming |
| CCP | Conventional crop production in Ontario |
| CDF | Conventional dryland farming |
| CF | Conventional farming |
| CFA | Conventional farming on arable land |
| CFB | Conventional farming of pollinated crops |
| CFB2 | Conventional farming of cassava for biofuel production |
| CFC | Conventional farming on cultivated land |
| CFE | Combined food and energy agroecosystem |
| CFF | Conventional farming focused on farmland fertility |
| CFG | Conventional farming of grain corn and hay |
| CFL | Cultivated flower land |
| CFL2 | Conventional farming of major food crops |
| CFM | Conventional farming methods |
| CG | Community gardens |
| CG1 | Conventional grazing |
| CIR | Conventional intensive row-crop agriculture |
| CL | Cropland |
| CLC | Cultivated land |
| CLF | Cropland farming |
| CNF | Conventional non-organic farming |
| CON | Conventional farming |
| CP | Conventional paddy farming |
| CP1 | Citrus plantations |
| CPB | Crop production for bio-based polymer feedstocks |
| CRC | Conventional rice-cropping systems |
| CRF | Conventional rice farming |
| CSG | Conventional sheep and beef grazing |
| CW | Conventional wheat farming |
| DLS | Dairy livestock systems |
| DSR | Direct-seeded rice |
| EBG | Extensive beef grazing |
| ELF | Extensive livestock farming |
| EWP | Energy-wood plantations |
| FF | Forest farming |
| FISH | Fishing |
| FOR | Forestry |
| GGL | Grassland-based grazing livestock management |
| GL | Grazing lands |
| GLS | Grazing livestock systems |
| GM | Grassland management |
| IA | Irrigated agriculture |
| IAV | Irrigated vegetable agriculture |
| ICF | Intensive conventional farming |
| IDC | Intensive dairy cattle production in arid conditions |
| IIA | Intensive irrigated agriculture |
| INT | Intensive farming |
| IOG | Irrigated olive groves |
| IRA | Irrigated rice agriculture |
| LAV | Leisure agriculture |
| LG | Livestock grazing |
| LI | Low-input farming |
| LIG | Low-intensity grazing |
| LMO | Livestock management aimed at reducing overgrazing |
| LPB | Livestock and poultry breeding |
| MA | Mountain agriculture |
| MB | Migratory beekeeping |
| MCB | Monocropping baseline |
| MCE | Mixed cropping involving pollinator-dependent crops in Europe |
| MCF | Mixed crop farming |
| MCS | Mixed-cropping system |
| MF | Monoculture farming |
| MFA | Multifunctional agriculture |
| NEM | Not explicitly mentioned |
| NG | Native grassland |
| NS | No-spray farming |
| OF | Organic farming |
| OPP | Oil palm plantations |
| ORC | Orchards |
| PCF | Public collective farms |
| PEM | Pasture ecosystem management |
| PFL | Paddy farming in Lenjan County |
| PGV | Plastic-greenhouse vegetable cultivation |
| PLA | Plowland |
| PLP | Peri-urban dairy livestock production systems |
| PMA | Pulpwood-based multifunctional agroforestry |
| PRF | Paddy rice fields |
| RBS | Riparian buffer strips |
| RF | Rice farming |
| RFA | Rain-fed agriculture |
| RPW | Rice paddy wetlands |
| SA | Subsistence agriculture |
| SAR | Semi-arid rangelands |
| SCA | Small-scale coffee agroforestry |
| SFT | Silvicultural fuel treatments |
| SGP | Systematic grazing practices |
| SHF | Smallholder farming |
| SHP | Smallholder poultry farming |
| SIF | Smallholder irrigation farming |
| SL | Shrubland |
| SOP | Smallholder oil-palm farming |
| SPS | Silvopastoral system |
| SSA | Smallholder and subsistence agriculture |
| SSI | Small-scale irrigation |
| TAF | Traditional alpine farming |
| TBI | Tree-based intercropping |
| TF | Tobacco farming |
| TFI | Traditional flood irrigation |
| TGS | Traditional grazing livestock systems |
| TIH | Traditional irrigated lands (huertas) |
| TOF | Traditional oasis farming |
| TOG | Traditional olive groves |
| TPP | Traditional pasture-based production |
| TRF | Traditional rice–fish co-culture |
| TSP | Traditional small-scale farming practices for native potato cultivation |
| TTR | Traditional terraced rice farming |
| UAS | Urban agriculture in school gardens |
| WMR | Wheat–maize rotation cropland |
| WRC | Wheat–rice farming with biocompost and fertilizer |

Table 6. Ecosystem service names and abbreviation codes

| **ID** | **Service name** | **Abbreviated service name** |
| --- | --- | --- |
| 1 | Carbon sequestration | CS |
| 1 | Water purification | WP |
| 1 | Biodiversity conservation | BDC |
| 1 | Gas exchange processes | GEP |
| 1 | CO^2^ uptake | CO2U |
| 1 | O^2^ emission | O2E |
| 1 | CH_4_ emission reduction | CH4ER |
| 1 | Gas regulation | GR |
| 1 | Primary product provision | PPP |
| 1 | Soil organic matter accumulation | SOMA |
| 1 | Nutrient cycling | NC |
| 1 | Flood control | FC |
| 1 | Water regulation | WR |
| 2 | Food provision | FPR |
| 2 | Raw materials provision | RMP |
| 2 | Gas regulation | GR |
| 2 | Climate regulation | CR |
| 2 | Water supply | WS |
| 2 | Soil formation | SF |
| 2 | Biodiversity conservation | BDC |
| 2 | Entertainment | ENT |
| 2 | Cultural heritage preservation | CHP |
| 3 | Biodiversity conservation | BDC |
| 3 | Medicinal plants | MP |
| 3 | Ecotourism | ET |
| 3 | Ancient places | AP |
| 4 | Water in environment | WE |
| 4 | Water in agriculture | WA |
| 5 | Soil conservation | SC |
| 6 | Agricultural landscapes | AL |
| 7 | Nutrient mineralization | NM |
| 7 | Water quality | WQ |
| 7 | Soil quality | SQ |
| 7 | Pollination | POL |
| 7 | Biological pest control | BPC |
| 7 | Agriculture provisioning | AGP |
| 7 | Climate regulation | CR |
| 8 | Recreational hunting | RH |
| 9 | Biotic production | BP |
| 9 | Fresh water recharge | FWR |
| 9 | Erosion resistance | ER |
| 9 | Mechanical water filtration | MWF |
| 9 | Physicochemical water filtration | PWF |
| 9 | Climate regulation | CR |
| 9 | Carbon sequestration | CS |
| 10 | Biomass production | BPRO |
| 10 | Groundwater recharge | GWR |
| 10 | Nutrient loss reduction | NLR |
| 10 | Soil loss reduction | SLR |
| 10 | Carbon sequestration | CS |
| 10 | Pollination | POL |
| 11 | Land consumption | LC |
| 12 | Water quality protection | WQP |
| 12 | Erosion control | EC |
| 12 | Tourism benefits | TB |
| 12 | Commercial fisheries | CF |
| 12 | Road maintenance | RM |
| 12 | Harbor protection | HP |
| 13 | Recreational fishing | RF |
| 14 | Pesticide reduction benefits | PRB |
| 15 | Pollination | POL |
| 16 | Groundwater management | GWM |
| 16 | Agricultural irrigation | AGI |
| 17 | Pollination | POL |
| 18 | Agricultural products | AGPR |
| 18 | Nutritional products | NP |
| 18 | Non-nutritional products | NNP |
| 18 | Fresh water provision | FWP |
| 18 | Domestic water uses | DWU |
| 19 | Food provision | FPR |
| 19 | Wood production | WP |
| 19 | Fiber production | FP |
| 19 | Infrastructure support | IS |
| 19 | Livestock support | LS |
| 19 | Shade for livestock | SFL |
| 19 | Shelter for livestock | SFL |
| 19 | Flood mitigation | FM |
| 19 | Nutrient filtering | NF |
| 19 | Contaminant filtering | CF |
| 19 | Waste decomposition | WD |
| 19 | Carbon accumulation | CA |
| 19 | Nitrous oxide regulation | NOR |
| 19 | Methane oxidation | MO |
| 19 | Pest regulation | PR |
| 19 | Disease regulation | DR |
| 20 | Food provision | FPR |
| 20 | Biodiversity maintenance | BDM |
| 20 | Disaster control | DC |
| 20 | Pollution control | PC |
| 20 | Cultural heritage preservation | CHP |
| 20 | Carbon absorption | CABS |
| 20 | Carbon storage | CSTO |
| 20 | Soil stabilization | SS |
| 20 | Erosion prevention | EP |
| 20 | Flood buffering | FB |
| 20 | Drought buffering | DB |
| 20 | Water purification | WP |
| 20 | Habitat provision | HP |
| 20 | Ecological maintenance | EM |
| 20 | Recreation | REC |
| 20 | Aesthetic value | AV |
| 21 | Water purification | WP |
| 21 | Subsistence agriculture | SA |
| 21 | Cultural heritage preservation | CHP |
| 22 | Irrigation water supply | IWS |
| 23 | Water quality improvement | WQI |
| 23 | Coral health protection | CHP |
| 24 | National food security | NFS |
| 24 | Rural amenities | RA |
| 24 | Recreational opportunities | RO |
| 24 | Nutrient recycling | NR |
| 24 | Carbon sequestration | CS |
| 24 | Groundwater recharge | GWR |
| 25 | Tea products | TP |
| 25 | Consumer goods | CG |
| 25 | Carbon storage | CSTO |
| 25 | Microclimate regulation | MCR |
| 25 | Green leafhoppers | GL |
| 25 | Earthworms | EW |
| 25 | Recreation | REC |
| 25 | Landscape purposes | LP |
| 26 | Water storage | WSTO |
| 26 | Water purification | WP |
| 26 | Flood mitigation | FM |
| 26 | Soil conservation | SC |
| 26 | Nutrient cycling | NC |
| 26 | Phytoremediation | PHY |
| 26 | Biodiversity conservation | BDC |
| 26 | Pollination | POL |
| 26 | Seed dissemination | SD |
| 26 | Pest control | PC |
| 26 | Air purification | AP |
| 26 | Carbon sequestration | CS |
| 26 | Carbon reduction | CRED |
| 26 | Microclimate regulation | MCR |
| 27 | Biological pest control | BPC |
| 27 | Soil formation | SF |
| 27 | Nutrient mineralization | NM |
| 27 | Pollination | POL |
| 27 | Shelterbelt effects | SE |
| 27 | Hydrological flow | HF |
| 27 | Aesthetic value | AV |
| 27 | Food provision | FPR |
| 27 | Raw materials provision | RMP |
| 27 | Carbon accumulation | CA |
| 27 | Nitrogen fixation | NFIX |
| 27 | Soil fertility | SFER |
| 28 | Carbon sequestration | CS |
| 28 | Greenhouse gas emissions reduction | GGER |
| 29 | Forest products | FPR |
| 29 | Sawlogs | SL |
| 29 | Pulpwood | PW |
| 29 | Round wood | RW |
| 29 | Fuel provision | FUP |
| 29 | Crop provision | CP |
| 29 | Meat production | MP |
| 29 | Dairy production | DP |
| 29 | Recreation | REC |
| 29 | Aesthetic value | AV |
| 31 | Food provision | FPR |
| 31 | Water regulation | WR |
| 31 | Erosion control | EC |
| 31 | Climate regulation | CR |
| 31 | Nutrient cycling | NC |
| 31 | Waste treatment | WT |
| 31 | Water supply | WS |
| 31 | Biological pest control | BPC |
| 31 | Water treatment | WT |
| 31 | Pollination | POL |
| 31 | Soil formation | SF |
| 31 | Habitat provision | HP |
| 31 | Refugium function | RF |
| 31 | Recreation | REC |
| 31 | Cultural heritage preservation | CHP |
| 32 | Food provision | FPR |
| 32 | Raw materials provision | RMP |
| 32 | Gas regulation | GR |
| 32 | Climate regulation | CR |
| 32 | Water regulation | WR |
| 32 | Waste treatment | WT |
| 32 | Soil formation | SF |
| 32 | Biodiversity conservation | BDC |
| 32 | Aesthetic landscape | AL |
| 33 | Food provision | FPR |
| 33 | Rice straw byproducts | RSB |
| 33 | Biological pest control | BPC |
| 33 | Soil formation | SF |
| 33 | Nutrient mineralization | NM |
| 33 | Carbon flow | CF |
| 33 | Nitrogen fixation | NFIX |
| 33 | Soil fertility | SFER |
| 33 | Hydrological flow | HF |
| 34 | Fruit production | FPR |
| 34 | Vegetable provision | VP |
| 34 | Landscape | LSC |
| 34 | Cultural heritage preservation | CHP |
| 34 | Historical identity | HI |
| 34 | Environmental education | EE |
| 34 | Recreation | REC |
| 34 | Leisure activities | LA |
| 35 | Vegetable provision | VP |
| 35 | Carbon fixation | CFIX |
| 35 | Soil retention | SR |
| 35 | Soil fertility protection | SFP |
| 35 | Water saving | WS |
| 36 | Improved forages | IF |
| 36 | Livestock feed | LF |
| 37 | Educational value | EV |
| 37 | Health value | HV |
| 37 | Welfare value | WV |
| 37 | Environmental quality | EQ |
| 37 | Climate change alleviation | CCA |
| 37 | Biodiversity conservation | BDC |
| 37 | Industrial ripple effects | IRE |
| 37 | Job creation | JC |
| 38 | Food provision | FPR |
| 38 | Fiber production | FP |
| 38 | Renewable raw materials | RRM |
| 38 | Climate regulation | CR |
| 38 | Carbon dioxide storage | CDS |
| 39 | Crop yields | CY |
| 39 | Tree yields | TY |
| 39 | Pasture yields | PY |
| 39 | Soil erosion control | SEC |
| 39 | Nitrate leaching control | NLC |
| 39 | Carbon sequestration | CS |
| 39 | Stakeholder perceptions | SP |
| 39 | Stakeholder motivations | SM |
| 40 | Water purification | WP |
| 40 | Water regulation | WR |
| 40 | Soil fertility | SFER |
| 40 | Fodder provision | FDP |
| 40 | Wild animal food | WAF |
| 40 | Recreational value | RV |
| 40 | Aesthetic value | AV |
| 41 | Carbon sequestration | CS |
| 42 | Flood risk mitigation | FRM |
| 42 | Climate mitigation | CM |
| 43 | Energy saving | ES |
| 43 | CO2 emission reduction | CO2ER |
| 44 | Carbon footprint reduction | CFR |
| 44 | CO2 emission reduction | CO2ER |
| 44 | Energy saving | ES |
| 44 | Waste management | WM |
| 44 | Coffee pulp biomass utilization | CPBU |
| 45 | Forest health improvement | FHI |
| 45 | Wildfire risk reduction | WRR |
| 45 | Air quality regulation | AQR |
| 45 | Renewable energy production | REP |
| 46 | Farming landscape conservation | FLC |
| 47 | Crop diversification | CD |
| 47 | Pest control | PC |
| 47 | Biodiversity conservation | BDC |
| 47 | Erosion control | EC |
| 47 | Carbon sequestration | CS |
| 47 | Rural jobs | RJ |
| 47 | Cultural heritage preservation | CHP |
| 47 | Landscape aesthetics | LA |
| 48 | Invasive species control | ISC |
| 48 | Biological pest control | BPC |
| 48 | Water regulation | WR |
| 48 | Biodiversity-based livelihoods | BBL |
| 48 | Biodiversity conservation | BDC |
| 49 | Water resource use | WRU |
| 49 | Biofuel crop production | BCP |
| 50 | Gas regulation | GR |
| 50 | Gas pollutant absorption | GPA |
| 50 | O2 emission | O2E |
| 50 | Climate regulation | CR |
| 50 | CO2 equivalent fluxes | CO2EF |
| 50 | Water regulation | WR |
| 50 | Soil water content | SWC |
| 50 | Water-holding capacity | WHC |
| 50 | Waste treatment | WT |
| 50 | Organic waste recycling | OWR |
| 50 | Erosion control | EC |
| 50 | Sediment retention | SR |
| 50 | Soil erosion reduction | SER |
| 50 | Soil formation | SF |
| 50 | Biological pest control | BPC |
| 50 | Chemical pesticide reduction | CPR |
| 50 | Nutrient cycling | NC |
| 50 | Refugium function | RF |
| 50 | Life expectancy reduction (disservice) | LERD |
| 51 | Flood protection | FP |
| 51 | Carbon sequestration | CS |
| 51 | Crop provision | CP |
| 51 | Livestock production | LP |
| 51 | Timber resources | TR |
| 52 | Food provision | FPR |
| 52 | Fresh water provision | FWP |
| 52 | Raw materials provision | RMP |
| 52 | Biochemicals provision | BCP |
| 52 | Climate regulation | CR |
| 52 | Water regulation | WR |
| 52 | Soil regulation | SR |
| 52 | Biological regulation | BR |
| 52 | Cultural heritage preservation | CHP |
| 53 | Carbon sequestration | CS |
| 53 | Biodiversity conservation | BDC |
| 53 | Environmental sustainability | ES |
| 54 | Forage production | FPR |
| 54 | Livestock support | LS |
| 54 | Soil conservation | SC |
| 55 | Carbon sequestration | CS |
| 55 | Agricultural products | AGPR |
| 55 | Biodiversity conservation | BDC |
| 55 | Timber resources | TR |
| 55 | Water provision | WP |
| 56 | Water provision | WP |
| 56 | Climate regulation | CR |
| 56 | GHG absorption | GHGA |
| 56 | Greenhouse gas emissions reduction | GGER |
| 57 | Water retention | WR |
| 57 | Soil fertility | SFER |
| 57 | Pest control | PC |
| 57 | Timber resources | TR |
| 57 | Non-timber forest products | NTFP |
| 58 | Land conversion valuation | LCV |
| 58 | Agriculture to oil palm | AOP |
| 59 | Recreation | REC |
| 59 | Climate regulation | CR |
| 60 | Agrotourism | AGT |
| 60 | Recreational service | RS |
| 61 | Agricultural heritage | AH |
| 61 | Biodiversity conservation | BDC |
| 61 | Existence value | EV |
| 61 | Bequest value | BV |
| 61 | Landscape aesthetics | LA |
| 61 | Natural hazard protection | NHP |
| 62 | Landscape aesthetics | LA |
| 63 | Carbon sequestration | CS |
| 63 | Water saving | WS |
| 63 | Soil fertility | SFER |
| 63 | Nitrogen fixation | NFIX |
| 63 | Greenhouse gas emissions reduction | GGER |
| 64 | Food provision | FPR |
| 64 | Drinking water | DW |
| 64 | Air quality regulation | AQR |
| 64 | Soil quality regulation | SQR |
| 64 | Flood control | FC |
| 64 | Disease control | DC |
| 64 | Active recreation | AREC |
| 64 | Passive recreation | PREC |
| 64 | Environmental education | EE |
| 65 | Agrotourism | AGT |
| 65 | Environmental conservation | EC |
| 66 | Livestock grazing | LG |
| 67 | Pollination | POL |
| 68 | Peatland ecosystem services | PES |
| 69 | Timber resources | TR |
| 69 | Cork | CRK |
| 69 | Firewood | FW |
| 69 | Conservation forestry | CF |
| 69 | Grazing | GRA |
| 69 | Livestock | LVS |
| 69 | Hunting | HUNT |
| 69 | Crop provision | CP |
| 70 | Agricultural water provision | AWP |
| 70 | Irrigation water supply | IWS |
| 71 | Food provision | FPR |
| 71 | Fodder provision | FDP |
| 71 | O2 emission | O2E |
| 71 | Moisture preservation | MP |
| 71 | Carbon sequestration | CS |
| 71 | Biodiversity conservation | BDC |
| 71 | Cultural heritage preservation | CHP |
| 71 | Greenhouse gas emissions reduction | GGER |
| 72 | Crop provision | CP |
| 72 | Rice provision | RPR |
| 72 | Soil carbon storage | SCS |
| 72 | Water retention | WR |
| 72 | Nutrient retention | NR |
| 73 | Carbon sequestration | CS |
| 73 | Waste disposal | WD |
| 73 | Greenhouse gas emissions reduction | GGER |
| 73 | Climate regulation | CR |
| 73 | Soil erosion control | SEC |
| 73 | Flood control | FC |
| 73 | Water storage | WSTO |
| 73 | Groundwater recharge | GWR |
| 73 | Rice provision | RPR |
| 73 | Fish provision | FPR |
| 73 | Crab provision | CPR |
| 73 | Straw provision | SPR |
| 74 | Carbon sequestration | CS |
| 74 | Water purification | WP |
| 74 | Biodiversity maintenance | BDM |
| 74 | Nutrient cycling | NC |
| 74 | Food provision | FPR |
| 74 | Pest control | PC |
| 75 | Olive grove conservation | OGC |
| 75 | Ecosystem services | ES |
| 76 | Energy conservation | EC |
| 76 | Livestock productivity | LP |
| 76 | Grassland rehabilitation | GR |
| 77 | Carbon sequestration | CS |
| 77 | O2 emission | O2E |
| 77 | Water conservation | WC |
| 77 | Nutrient cycling | NC |
| 77 | Soil conservation | SC |
| 77 | Waste purification | WP |
| 77 | Environmental purification | EP |
| 77 | Straw returning | SR |
| 78 | Soil quality enhancement | SQE |
| 78 | Nutrient cycling | NC |
| 78 | Biodiversity conservation | BDC |
| 79 | Flower sightseeing | FS |
| 79 | Recreational benefit | RB |
| 80 | Soil conservation | SC |
| 80 | Biodiversity conservation | BDC |
| 80 | Water regulation | WR |
| 80 | Carbon sequestration | CS |
| 80 | Sediment retention | SR |
| 81 | Product provision | PPR |
| 81 | Climate regulation | CR |
| 81 | Soil conservation | SC |
| 81 | Carbon fixation | CFIX |
| 81 | O2 emission | O2E |
| 81 | Nutrient maintenance | NM |
| 82 | Aesthetic value | AV |
| 82 | Recreation | REC |
| 82 | Educational value | EV |
| 82 | Spiritual well-being | SWB |
| 82 | Biodiversity conservation | BDC |
| 83 | Pollination | POL |
| 84 | Farmland non-market values | FNMV |
| 84 | Farmland area | FA |
| 84 | Farmland fertility | FF |
| 84 | Water quality | WQ |
| 84 | Air quality regulation | AQR |
| 84 | Species richness | SPR |
| 84 | Recreational value | RV |
| 85 | Timber resources | TR |
| 85 | Watershed protection | WSP |
| 85 | Carbon sequestration | CS |
| 85 | Biodiversity conservation | BDC |
| 85 | Hunting | HUNT |
| 85 | Fishing | FISH |
| 85 | Grazing | GRA |
| 85 | Recreation | REC |
| 85 | Non-timber forest products | NTFP |
| 85 | Resin | RES |
| 85 | Mushrooms | MUSH |
| 85 | Aromatics | ARO |
| 85 | Wind energy | WE |
| 85 | Quarrying | QUAR |
| 86 | Water preservation | WP |
| 86 | Land protection | LP |
| 86 | Flood prevention | FP |
| 86 | Soil erosion prevention | SEP |
| 86 | Microclimate regulation | MCR |
| 87 | Pollination | POL |
| 88 | Irrigation water supply | IWS |
| 89 | Livestock waste utilization | LWU |
| 89 | Poultry waste utilization | PWU |
| 90 | Pollination | POL |
| 90 | Nutrient cycling | NC |
| 90 | Soil formation | SF |
| 90 | Pest regulation | PR |
| 90 | Watershed protection | WSP |
| 90 | Landscape beauty | LB |
| 90 | Biodiversity protection | BDP |
| 91 | Carbon sequestration | CS |
| 91 | Dryland salinity reduction | DSR |
| 92 | Grassland ecosystem restoration | GER |
| 92 | Soil carbon sequestration | SCS |
| 92 | Biodiversity conservation | BDC |
| 92 | Climate regulation | CR |
| 93 | Cultural heritage preservation | CHP |
| 93 | Sustainable pasture use | SPU |
| 93 | Wildlife protection | WP |
| 93 | Traditional products | TP |
| 94 | Food provision | FPR |
| 94 | Genetic resources | GR |
| 94 | Raw materials provision | RMP |
| 94 | Water supply | WS |
| 94 | Water regulation | WR |
| 94 | Climate regulation | CR |
| 94 | Disturbance regulation | DR |
| 94 | Gas regulation | GR |
| 94 | Biological pest control | BPC |
| 94 | Erosion control | EC |
| 94 | Water treatment | WT |
| 94 | Nutrient cycling | NC |
| 94 | Pollination | POL |
| 94 | Soil formation | SF |
| 94 | Habitat provision | HP |
| 94 | Refugium function | RF |
| 94 | Recreation | REC |
| 94 | Cultural heritage preservation | CHP |
| 95 | Livestock production | LP |
| 95 | Arable crops | AC |
| 95 | Timber resources | TR |
| 95 | Water supply | WS |
| 95 | Carbon sequestration | CS |
| 95 | Local flood mitigation | LFM |
| 95 | Potable water quality | PWQ |
| 95 | Recreation | REC |
| 95 | Greenhouse gas emissions reduction | GGER |
| 96 | Livestock production | LP |
| 96 | Poultry production | PP |
| 97 | Carbon sequestration | CS |
| 97 | Nutrient dynamics | ND |
| 97 | Soil health improvement | SHI |
| 98 | Pest control | PC |
| 98 | Biological regulation | BR |
| 98 | Food provision | FPR |
| 98 | Medicinal plants | MP |
| 98 | Recreation | REC |
| 99 | Water conservation | WC |
| 99 | Wind prevention | WP |
| 99 | Sand fixation | SFIX |
| 99 | Carbon sequestration | CS |
| 99 | Habitat provision | HP |
| 99 | Climate mitigation | CM |
| 100 | Bee pollination | BP |
| 101 | Recreational value | RV |
| 101 | Existence value | EV |
| 101 | Agricultural landscapes | AL |
| 102 | Nitrate leaching control | NLC |
| 102 | Soil erosion control | SEC |
| 103 | Soil stabilization | SS |
| 103 | Erosion control | EC |
| 103 | Microclimate regulation | MCR |
| 103 | Regional climate regulation | RCR |
| 103 | Timber resources | TR |
| 103 | Crop provision | CP |
| 103 | Recreational value | RV |
| 104 | Erosion control | EC |
| 104 | Air quality regulation | AQR |
| 104 | Climate regulation | CR |
| 104 | Water regulation | WR |
| 104 | Soil fertility | SFER |
| 105 | Pollination | POL |
| 106 | Biodiversity conservation | BDC |
| 106 | Carbon sequestration | CS |
| 106 | Soil fertility | SFER |
| 106 | Nutrient cycling | NC |
| 106 | Erosion prevention | EP |
| 106 | Shelterbelt effects | SE |
| 106 | Pollination | POL |
| 106 | Pest control | PC |
| 106 | Disease control | DC |
| 106 | Soil formation | SF |
| 106 | Aesthetic value | AV |
| 107 | Microclimate regulation | MCR |
| 107 | Methane emissions reduction | MER |
| 107 | Carbon sequestration | CS |
| 107 | Nitrogen fixation | NFIX |
| 107 | Biodiversity conservation | BDC |
| 108 | Food provision | FPR |
| 108 | Fodder provision | FDP |
| 108 | Bio-energy | BE |
| 108 | Wood production | WP |
| 108 | Water holding capacity | WHC |
| 108 | Carbon sequestration | CS |
| 108 | Soil erosion prevention | SEP |
| 108 | Shelter belt effects | SBE |
| 108 | Nitrogen fixation | NFIX |
| 108 | Pollination | POL |
| 108 | Biological pest control | BPC |
| 108 | Nitrogen mineralization | NM |
| 108 | Soil formation | SF |
| 108 | Aesthetic value | AV |
| 109 | Carbon sequestration | CS |
| 110 | Carbon sequestration | CS |
| 110 | Water regulation | WR |
| 110 | Soil erosion control | SEC |
| 110 | Cultural heritage preservation | CHP |
| 111 | Agricultural landscapes | AL |
| 111 | Biodiversity conservation | BDC |
| 111 | Soil fertility | SFER |
| 111 | Quality food products | QFP |
| 112 | Insect pollination | IP |
| 113 | Pollination | POL |
| 114 | Carbon sequestration | CS |
| 114 | Water purification | WP |
| 115 | Biological pest control | BPC |
| 115 | Nitrogen mineralization | NM |
| 116 | Biological pest control | BPC |
| 117 | Insect-based feeds | IBF |
| 117 | Alternative protein source | APS |
| 117 | Animal feed | AF |
| 118 | Agricultural water supply | AWS |
| 119 | Cultural landscape | CL |
| 119 | Biodiversity maintenance | BDM |
| 119 | Forest fire prevention | FFP |
| 119 | Quality food products | QFP |
| 120 | Water quality | WQ |
| 120 | Biodiversity conservation | BDC |
| 120 | Landscape | LSC |
| 120 | Local food products | LFP |
| 121 | Carbon sequestration | CS |
| 121 | CH4 emission reduction | CH4ER |
| 121 | Nutrient retention | NR |
| 121 | Pest control | PC |
| 121 | Water regulation | WR |
| 121 | Tourism benefits | TB |
| 121 | Water pollution reduction | WPR |
| 122 | Pollination | POL |
| 123 | Cultivated land protection | CLP |
| 123 | Climate regulation | CR |
| 123 | Air purification | AP |
| 123 | Water conservation | WC |
| 123 | Soil conservation | SC |
| 123 | Wildlife habitat | WH |
| 123 | Aesthetic value | AV |
| 123 | National food security | NFS |
| 123 | Farmer employment | FE |
| 124 | Irrigation water supply | IWS |
| 125 | Pollination | POL |
| 126 | Pollination | POL |
| 126 | Honeybee products | HP |
| 126 | Cultural heritage preservation | CHP |
| 126 | Educational services | ES |
| 127 | Pollination | POL |
| 128 | Climate regulation | CR |
| 128 | Water flow regulation | WFR |
| 128 | Waste treatment | WT |
| 128 | Biodiversity conservation | BDC |
| 128 | Recreation | REC |
| 128 | Food provision | FPR |
| 128 | Water provision | WP |
| 128 | Raw materials provision | RMP |
| 129 | Water purification | WP |
| 129 | Agricultural irrigation | AGI |
| 130 | Carbon sequestration | CS |
| 130 | Sand fixation | SFIX |
| 130 | Water conservation | WC |
| 130 | Soil conservation | SC |
| 130 | Biodiversity conservation | BDC |
| 130 | Primary product provision | PPP |
| 130 | Secondary product provision | SPP |
| 130 | Tourism benefits | TB |
| 130 | Entertainment | ENT |
| 130 | Ethnic culture inheritance | ECI |
| 131 | Pollination | POL |
| 132 | Biological pest control | BPC |
| 132 | Nitrogen regulation | NR |
| 132 | Soil formation | SF |
| 132 | Food provision | FPR |
| 132 | Forage production | FPR |
| 132 | Raw materials provision | RMP |
| 132 | Carbon accumulation | CA |
| 132 | Hydrological flow | HF |
| 132 | Landscape aesthetics | LA |
| 132 | Pollination | POL |
| 133 | Pasture maintenance | PM |
| 133 | Grazing | GRA |
| 133 | Biodiversity conservation | BDC |
| 133 | Water management | WM |
| 133 | Land management | LM |
| 133 | Recreational service | RS |
| 134 | Forage production | FPR |
| 134 | Erosion control | EC |
| 134 | Sediment control | SC |
| 134 | Runoff regulation | RR |
| 134 | Carbon sequestration | CS |
| 134 | O2 emission | O2E |
| 135 | Grassland ecosystem restoration | GER |
| 136 | Irrigation water supply | IWS |
| 137 | Food provision | FPR |
| 137 | Fuel provision | FUP |
| 137 | Soil conservation | SC |
| 137 | Community diversity | CD |
| 137 | Nutrient cycling | NC |
| 137 | CO2 sequestration | CO2S |
| 138 | Agricultural heritage | AH |
| 138 | Cultural heritage preservation | CHP |
| 139 | Irrigation water supply | IWS |
| 139 | Pollination | POL |
| 139 | Natural pest control | NPC |
| 139 | Bird pest control | BPC |
| 139 | Bat pest control | BPC |
| 140 | Recreation | REC |
| 140 | Tourism benefits | TB |
| 140 | Landscape beauty | LB |
| 140 | Healthy food access | HFA |
| 140 | Organic food access | OFA |
| 140 | Natural resource protection | NRP |
| 140 | Water protection | WP |
| 140 | Soil protection | SP |
| 141 | Carbon sequestration | CS |
| 141 | Water conservation | WC |
| 141 | Nutrient recycling | NR |
| 141 | O2 emission | O2E |
| 141 | Bird habitat | BH |
| 142 | Gas regulation | GR |
| 142 | Climate regulation | CR |
| 142 | Disturbance prevention | DP |
| 142 | Water regulation | WR |
| 142 | Water supply | WS |
| 142 | Soil retention | SR |
| 142 | Soil formation | SF |
| 142 | Nutrient regulation | NR |
| 142 | Waste treatment | WT |
| 142 | Pollination | POL |
| 142 | Biological pest control | BPC |
| 142 | Refugium function | RF |
| 142 | Wildlife conservation | WC |
| 142 | Aesthetic value | AV |
| 142 | Recreational value | RV |
| 142 | Cultural heritage preservation | CHP |
| 142 | Spiritual value | SV |
| 143 | Food provision | FPR |
| 143 | Water supply | WS |
| 143 | Erosion control | EC |
| 143 | Climate regulation | CR |
| 143 | Habitat provision | HP |
| 143 | Refugium function | RF |
| 143 | Raw materials provision | RMP |
| 143 | Genetic resources | GR |
| 143 | Medicinal plants | MP |
| 143 | Water regulation | WR |
| 143 | Water treatment | WT |
| 143 | Nutrient cycling | NC |
| 143 | Pollination | POL |
| 143 | Soil formation | SF |
| 143 | Recreation | REC |
| 143 | Cultural heritage preservation | CHP |
| 144 | Drought mitigation | DM |
| 144 | Water quality | WQ |
| 144 | Environmental conservation | EC |
| 144 | Rural landscapes | RL |
| 145 | Irrigation water supply | IWS |
| 145 | Rural water supply | RWS |
| 145 | Livestock water supply | LWS |
| 145 | Water quality maintenance | WQM |
| 145 | Wastewater treatment | WT |
| 145 | Flow regulation | FR |
| 145 | Natural hazard mitigation | NHM |
| 146 | Nutrient cycling | NC |
| 146 | Water infiltration | WI |
| 146 | Biodiversity conservation | BDC |
| 146 | Soil structure maintenance | SSM |
| 146 | Agricultural products | AGPR |
| 146 | Water quality protection | WQP |
| 146 | Food provision | FPR |
