# Supplementary material for: Economic valuation of ecosystem services in agriculture and agroforestry: A meta-analysis
Source: PLoS One. 2026 Oct 16;21(10):e0360792. doi: 10.1371/journal.pone.0360792 (PMC13674745; doi:10.1371/journal.pone.0360792)
Supplement: Supplementary file 3 [file pone.0360792.s022.docx]

# **References (146)**

1.	Shihong Y, Yanan X, Junzeng X. The Economic Value of Gas Exchange in a Paddy Field Ecosystem Using Water-Saving Irrigation. Pol J Environ Stud. 2018;27(5):2267-75. doi: 10.15244/pjoes/79273.

2.	Luo D, Zhang W. A comparison of Markov model-based methods for predicting the ecosystem service value of land use in Wuhan, central China. Ecosyst Serv. 2014;7:57-65. doi: 10.1016/j.ecoser.2013.11.001.

3.	Bostan Y, Fatahi Ardakani A, Fehresti Sani M, Sadeghinia M. A comparison of stated preferences methods for the valuation of natural resources: the case of contingent valuation and choice experiment. Int J Environ Sci Technol. 2020;17(9):4031-46. doi: 10.1007/s13762-020-02714-z.

4.	Karami O, Yazdani S, Saleh I, Rafiee H, Riahi A. A comparison of Zayandehrood River water values for agriculture and the environment. River Res Appl. 2020;36(7):1279-85. doi: 10.1002/rra.3627.

5.	Khan A, Ali G. A contingent valuation study of soil conservation program in swat district, Khyber Pakhtunkhwa, Pakistan. Sarhad J Agric. 2019;35(3):816-22. doi: 10.17582/journal.sja/2019/35.3.816.822.

6.	Severen C, Costello C, Deschênes O. A Forward-Looking Ricardian Approach: Do land markets capitalize climate change forecasts? J Environ Econ Manag. 2018;89:235-54. doi: 10.1016/j.jeem.2018.03.009.

7.	Alam M, Olivier A, Paquette A, Dupras J, Revéret J-P, Messier C. A general framework for the quantification and valuation of ecosystem services of tree-based intercropping systems. Agrofor Syst. 2014;88(4):679-91. doi: 10.1007/s10457-014-9681-x.

8.	Fagarazzi C, Sergiacomi C, Stefanini FM, Marone E. A Model for the Economic Evaluation of Cultural Ecosystem Services: The Recreational Hunting Function in the Agroforestry Territories of Tuscany (Italy). Sustainability. 2021;13(20):11229-14. doi: 10.3390/su132011229.

9.	Cao V, Margni M, Favis BD, Deschênes L. Aggregated indicator to assess land use impacts in life cycle assessment (LCA) based on the economic value of ecosystem services. J Clean Prod. 2015;94:56-66. doi: 10.1016/j.jclepro.2015.01.041.

10.	Kay S, Graves A, Palma JH, Moreno G, Roces-Díaz JV, Aviron S, et al. Agroforestry is paying off–Economic evaluation of ecosystem services in European landscapes with and without agroforestry systems. Ecosyst Serv. 2019;36(100896):1-22. doi: 10.1016/j.ecoser.2019.100896.

11.	Bravi M, Bottero M, Dell’Anna F. An Application of the Life Satisfaction Approach (LSA) to Value the Land Consumption and Ecosystem Services. J Knowl Econ. 2024;15(1):2988-3013. doi: 10.1007/s13132-023-01150-x.

12.	Rein FA. An economic analysis of vegetative buffer strip implementation - Case study: Elkhorn Slough, Monterey Bay, California. Coast Manag. 1999;27(4):377-90. doi: 10.1080/089207599263785. PubMed PMID: CCC:000083147600007.

13.	Terashima Y, Yamashita Y, Asano K. An economic evaluation of recreational fishing in Tango Bay, Japan. Fish Sci. 2020;86(5):925-37. PubMed PMID: CCC:000565776100019.

14.	Brethour C, Weersink A. An economic evaluation of the environmental benefits from pesticide reduction. Agric Econ. 2001;25(2-3):219-26. doi: 10.1111/j.1574-0862.2001.tb00202.x. PubMed PMID: CCC:000172267200009.

15.	Mulatu DW. An Economic Valuation and Mapping of Pollination Services in Ethiopia. In: Hufnagel L, editor. Changing Ecosystems and Their Services 2019. p. 1-14.

16.	Ma S, Duggan JM, Eichelberger BA, McNally BW, Foster JR, Pepi E, et al. Valuation of ecosystem services to inform management of multiple-use landscapes. Ecosyst Serv. 2016;19(C):6-18.

17.	Barfield A, Bergstrom JC, Ferreira S, editors. An Economic Valuation of Pollination Services in Georgia. Southern Agricultural Economics Association Annual Meeting; 2012; Birmingham.

18.	García-rubio N, Larraz B, Gámez M, Raimonet M, Cakir R, Sauvage S, et al. An economic valuation of the provisioning ecosystem services in the south-west of Europe. Environ Dev Sustain. 2024:1-27. doi: 10.1007/s10668-024-04877-y.

19.	Dominati EJ, Mackay A, Lynch B, Heath N, Millner I. An ecosystem services approach to the quantification of shallow mass movement erosion and the value of soil conservation practices. Ecosyst Serv. 2014;9:204-15. doi: 10.1016/j.ecoser.2014.06.006. PubMed PMID: CCC:000363661900020.

20.	Myeong S, Yi D. An Estimation of Ecosystem Service Value of Rice Paddy Wetland in Korea Using Contingent Valuation Method. Water. 2023;15(24):4263-76. doi: 10.3390/w15244263.

21.	Christie M, Fazey I, Cooper R, Hyde T, Kenter JO. An evaluation of monetary and non-monetary techniques for assessing the importance of biodiversity and ecosystem services to people in countries with developing economies. Ecol Econ. 2012;83:67-78. doi: 10.1016/j.ecolecon.2012.08.012. PubMed PMID: CCC:000312054700008.

22.	Harun R, Muresan IC, Arion FH, Dumitras DE, Lile R. Analysis of Factors that Influence the Willingness to Pay for Irrigation Water in the Kurdistan Regional Government, Iraq. Sustainability. 2015;7(7):9574-86. doi: 10.3390/su7079574. PubMed PMID: CCC:000360354500073.

23.	Rolfe J, Windle J. Assessing community values for reducing agricultural emissions to improve water quality and protect coral health in the Great Barrier Reef. Water Resour Res. 2011;47:12506-. doi: 10.1029/2010WR010355. PubMed PMID: CCC:000297987900001.

24.	Moon W, Griffith JW. Assessing holistic economic value for multifunctional agriculture in the US. Food Policy. 2011;36(4):455-65. doi: 10.1016/j.foodpol.2011.05.003. PubMed PMID: CCC:000293120300001.

25.	Liu WY, Chiang CY, Yap JL, Lin CC. Assessing the ecosystem service values of tea plantations using conventional and organic farming methods: Is organic farming always better? Ecol Indic. 2024;158:111355-67. doi: 10.1016/j.ecolind.2023.111355.

26.	Hsieh C-H, Lin H-W, Liu W-Y. Assessing the ecosystem services provided by conventional and organic farmlands: A better outcome for organic farmlands? Ecosyst Serv. 2023;60:101514. doi: 10.1016/j.ecoser.2023.101514.

27.	Sandhu HS, Wratten SD, Cullen R, Case B. The future of farming: The value of ecosystem services in conventional and organic arable land. An experimental approach. Ecol Econ. 2008;64(4):835-48. doi: 10.1016/j.ecolecon.2007.05.007.

28.	Kaske KJ, Garcia de Jalon S, Williams AG, Graves AR. Assessing the Impact of Greenhouse Gas Emissions on Economic Profitability of Arable, Forestry, and Silvoarable Systems. Sustainability. 2021;13(7):3637-. doi: 10.3390/su13073637. PubMed PMID: CCC:000638928900001.

29.	Nikodinoska N, Paletto A, Pastorella F, Granvik M, Franzese PP. Assessing, valuing and mapping ecosystem services at city level: The case of Uppsala (Sweden). Ecol Model. 2018;368:411-24. doi: 10.1016/j.ecolmodel.2017.10.013. PubMed PMID: CCC:000424187200036.

30.	Kaenchan P, Guinee J, Gheewala SH. Assessment of ecosystem productivity damage due to land use. Sci Total Environ. 2018;621:1320-9. doi: 10.1016/j.scitotenv.2017.10.096.

31.	Teshome DS, Tolessa T, Gemeda DO, Taddese H, You S. Impact of land use/land cover (LU/LC) changes on ecosystem service values in Muger Sub-basin, Upper Blue Nile Basin, Ethiopia. Environ Chall. 2024;17:101041. doi: 10.1016/j.envc.2024.101041.

32.	Chen X, He L, Luo F, He Z, Bai W, Xiao Y, et al. Dynamic characteristics and impacts of ecosystem service values under land use change: A case study on the Zoigê plateau, China. Ecol Inform. 2023;78:102350. doi: 10.1016/j.ecoinf.2023.102350.

33.	Nayak AK, Shahid M, Nayak AD, Dhal B, Moharana KC, Mondal B, et al. Assessment of ecosystem services of rice farms in eastern India. Ecol Process. 2019;8(35):1-16. doi: 10.1186/s13717-019-0189-1.

34.	Martínez-Paz JM, Banos-González I, Martínez-Fernández J, Esteve-Selma MÁ. Assessment of management measures for the conservation of traditional irrigated lands: The case of the Huerta of Murcia (Spain). Land Use Policy. 2019;81:382-91. doi: 10.1016/j.landusepol.2018.10.050.

35.	Chang J, Wu X, Liu A, Wang Y, Xu B, Yang W, et al. Assessment of net ecosystem services of plastic greenhouse vegetable cultivation in China. Ecol Econ. 2011;70(4):740-8. doi: 10.1016/j.ecolecon.2010.11.011.

36.	Ouédraogo A, Zampaligré N, Mulubrhan B, Adesogan AT. Assessment of peri-urban livestock producers’ willingness to pay for improved forages as cash crops. Agron J. 2022;114(1):63-74. doi: 10.1002/agj2.20953.

37.	Hong IK, Yun HK, Jung YB, Lee SM, Lee SD. Assessment of Socio-Economic Values of Agricultural Experience Services in School Garden Education. J People Plants Environ. 2022;25(3):239-52. doi: 10.11628/ksppe.2022.25.3.239.

38.	Niccolucci V, Coscieme L, Marchettini N. Benefit transfer and the economic value of Biocapacity: Introducing the ecosystem service Yield factor. Ecosyst Serv. 2021;48:101256-6. doi: 10.1016/j.ecoser.2021.101256.

39.	Crous-Duran J. Bio-economic process-based modelling methodology for measuring and evaluating the ecosystem services provided by agroforestry systems: Forestry Engineering and Natural Resources. University of Lisbon; 2022.

40.	Garcia-Llorente M, Martin-Lopez B, Diaz S, Montes C. Can ecosystem properties be fully translated into service values? An economic valuation of aquatic plant services. Ecol Appl. 2011;21(8):3083-103. doi: 10.1890/10-1744.1.

41.	Winans KS, Tardif A-S, Lteif AE, Whalen JK. Carbon sequestration potential and cost-benefit analysis of hybrid poplar, grain corn and hay cultivation in southern Quebec, Canada. Agrofor Syst. 2015;89(3):421-33. doi: 10.1007/s10457-014-9776-4. PubMed PMID: CCC:000354947500004.

42.	Saptutyningsih E, Akhtar R, Setyawati Dewanti D, Anggoro T, editors. Climate Change and Agriculture: An Economic Valuation of Flood Risk Mitigation. E3S Web of Conferences; 2024: EDP Sciences.

43.	Rahmah DM, Mardawati E, Kastaman R, Pujianto T, Pramulya R. Coffee Pulp Biomass Utilization on Coffee Production and Its Impact on Energy Saving, CO2 Emission Reduction, and Economic Value Added to Promote Green Lean Practice in Agriculture Production. Agronomy-Basel. 2023;13(3):904-30. doi: 10.3390/agronomy13030904.

44.	Petcharat A, Jo J-H, Lee Y. Comparing Local Residents' Willingness to Pay (WTP) and Willingness to Volunteer (WTV) for Water Onion (*Crinum thaianum*) Habitat Conservation. Forests. 2022;13(5):706-29. doi: 10.3390/f13050706.

45.	Campbell RM, Anderson NM. Comprehensive comparative economic evaluation of woody biomass energy from silvicultural fuel treatments. J Environ Manag. 2019;250:9422-34. doi: 10.1016/j.jenvman.2019.109422.

46.	Chen B, Qiu Z, Usio N, Nakamura K. Conservation and contingent valuation of farming landscape amenities by visitors: A case study of terraced paddy fields in Central Japan. Paddy Water Environ. 2018;16(3):561-70. doi: 10.1007/s10333-018-0648-9.

47.	Alcon F, Albaladejo-Garcia JA, Martinez-Garcia V, Rossi ES, Blasi E, Lehtonen H, et al. Cost benefit analysis of diversified farming systems across Europe: Incorporating non-market benefits of ecosystem services. Sci Total Environ. 2024;912:69272-302. doi: 10.1016/j.scitotenv.2023.169272.

48.	van Wilgen BW, de Wit MP, Anderson HJ, Le Maitre DC, Kotze IM, Ndala S, et al. Costs and benefits of biological control of invasive alien plants: case studies from South Africa. S Afr J Sci. 2004;100(1):113-22.

49.	Kaenchan P, Gheewala SH. Cost - Benefit of water resource use in biofuel feedstock production. J Clean Prod. 2017;142:1192-9. doi: 10.1016/j.jclepro.2016.06.129.

50.	Zhen H, Qiao Y, Zhao H, Ju X, Zanoli R, Waqas MA, et al. Developing a conceptual model to quantify eco-compensation based on environmental and economic cost-benefit analysis for promoting the ecologically intensified agriculture. Ecosyst Serv. 2022;56:101442-53. doi: 10.1016/j.ecoser.2022.101442.

51.	Pinke Z, Kiss M, Lövei GL. Developing an integrated land use planning system on reclaimed wetlands of the Hungarian Plain using economic valuation of ecosystem services. Ecosyst Serv. 2018;30:299-308. doi: 10.1016/j.ecoser.2017.09.007.

52.	Schild JEM, Vermaat JE, van Bodegom PM. Differential effects of valuation method and ecosystem type on the monetary valuation of dryland ecosystem services: A quantitative analysis. J Arid Environ. 2018;159:11-21. doi: 10.1016/j.jaridenv.2017.09.001.

53.	Tziolas E, Ispikoudis S, Mantzanas K, Koutsoulis D, Pantera A. Economic and Environmental Assessment of Olive Agroforestry Practices in Northern Greece. Agriculture. 2022;12(6):851-. doi: 10.3390/agriculture12060851.

54.	Dong SK, Kang MY, Yun XJ, Long RJ, Hu ZZ. Economic comparison of forage production from annual crops, perennial pasture and native grassland in the alpine region of the Qinghai-Tibetan Plateau, China. Grass Forage Sci. 2007;62(4):405-15. doi: 10.1111/j.1365-2494.2007.00594.x. PubMed PMID: CCC:000250941200001.

55.	Baral H, Keenan RJ, Sharma SK, Stork NE, Kasel S. Economic evaluation of ecosystem goods and services under different landscape management scenarios. Land Use Policy. 2014;39:54–64. doi: 10.1016/j.landusepol.2014.03.008.

56.	Navarrete-Molina C, Meza-Herrera CA, Ramirez-Flores JJ, Herrera-Machuca MA, Lopez-Villalobos N, Lopez-Santiago MA, et al. Economic evaluation of the environmental impact of a dairy cattle intensive production cluster under arid lands conditions. Animal. 2019;13(10):2379-87. doi: 10.1017/S175173111900048X. PubMed PMID: CCC:000487064700034.

57.	Hamid Arrum H. Economic Valuation and Payment for Ecosystem Services (Pes) in Smallholder Farmers: A Case Study in The Batang Toru Forest, North Sumatra: Universitas Andalas; 2025.

58.	Sari PH, Erpinda M. Economic valuation of agricultural land conversion to oil palm plantations: Assessing economic benefits and environmental impacts. Kemakmuran Hij: J Ekon Pembang. 2025;2(1):1–14. doi: 10.61511/jekop.v2i1.2025.1767.

59.	Albaladejo-García JA, Alcon F, Martínez-Paz JM. Economic valuation of allotment gardens in peri-urban degraded agroecosystems: The role of citizens’ preferences in spatial planning. Sustain Cities Soc. 2021;68:102771-9. doi: 10.1016/j.scs.2021.102771.

60.	Handoyo Mulyo J, Muna N, Perwitasari H, Rohmah F, editors. Economic valuation of Bhumi Merapi Agrotourism in Sleman Regency. IOP Conference Series: Earth and Environmental Science; 2020: IOP Publishing Ltd.

61.	Rewitzer S, Huber R, Grêt-Regamey A, Barkmann J. Economic valuation of cultural ecosystem service changes to a landscape in the Swiss Alps. Ecosyst Serv. 2017;26:197-208. doi: 10.1016/j.ecoser.2017.06.014.

62.	Hatan S, Fleischer A, Tchetchik A. Economic valuation of cultural ecosystem services: The case of landscape aesthetics in the agritourism market. Ecol Econ. 2021;184:107005-. doi: 10.1016/j.ecolecon.2021.107005.

63.	Birthal P, Kumar A, Kumara T M K, Meena DC. Economic valuation of ecosystem services of selected interventions in agriculture in India. International Food Policy Research Institute (IFPRI), 2024.

64.	Konstantinova E, Brunina L, Persevica A, Zvaigzne A, editors. Economic valuation of ecosystem services: A case study for sustainable management of degraded peatlands in Latvia. Vide Tehnologija Resursi - Environment, Technology, Resources; 2019: Rezekne Higher Education Institution.

65.	Al-Azizah LH, Mulyo JH, Perwitasari H, editors. Economic Valuation of Kebun Buah Mangunan at Sub-district Dlingo, Bantul. IOP Conference Series: Earth and Environmental Science; 2021: IOP Publishing Ltd.

66.	Barrera-Perales OT, Sagarnaga-Villegas LM, Tudela-Mamani JW, Salas-Gonzalez JM, Islas-Moreno A, Leos-Rodriguez JA. Economic valuation of rangelands in the north of Mexico: A study for its conservation. Span J Agric Res. 2021;19(3):110-22. doi: 10.5424/sjar/2021193-17041. PubMed PMID: CCC:000684940400009.

67.	Huber R, Le’Clec’h S, Buchmann N, Jour. Economic value of three grassland ecosystem services when managed at the regional and farm scale. Sci Rep. 2022;12(4149):1-13. doi: 10.1038/s41598-022-08198-w.

68.	Mohammad Y, Adcharaporn P, Himlal B. Economics of Peatland Ecosystem Services: A Study of Use and Non-Use Values and People Interplays in Sumatra, Indonesia. Land. 2024;13:866-86. doi: 10.3390/land13060866.

69.	Ovando P, Campos P, Oviedo J, Caparrós A. Ecosystem accounting for measuring total income in private and public agroforestry farms. For Policy Econ. 2016;71(C):43-51. doi: 10.1016/j.forpol.2016.06.031.

70.	Mahjoubi I, Frör O. Ecosystem service change from lost surface water for farming in the Middle Drâa Valley, southern Morocco: an economic valuation through a replacement cost approach. J Water Clim Change. 2024;15(10):5129-48. doi: 10.2166/wcc.2024.231.

71.	Sharafi S, Marzban Z, Dragovich D. Ecosystem Service Values and Wheat Agroecosystem Management Types in a Semi-Arid Region, Iran. Land. 2025;14(4):1-22. doi: 10.3390/land14040865.

72.	Lin K-T, Pan S-Y, Yuan M-H, Guo H-Y, Huang Y-C. Quantifying and monetarizing cropland ecosystem services towards sustainable soil management. Ecol Indic. 2024;159:111751. doi: 10.1016/j.ecolind.2024.111751.

73.	Zhou T, Hu H, Hu J, Yang Z, Lv Q, Wang Y, et al. Ecosystem services and cost-effective benefits from the reclamation of saline sodic land under different paddy field systems. Ecosyst Serv. 2024;70:101682-91. doi: 10.1016/j.ecoser.2024.101682.

74.	Xiao Y, Xie G-D, An K, Liu C-L, Chen C-C. Ecosystem services of wheat-maize cropland systems in the North China Plain. Chin J Eco-Agric. 2011;19(2):429-35. doi: 10.3724/sp.j.1011.2011.00429.

75.	Torres-Miralles M, Grammatikopoulou I, Rescia AJ. Employing contingent and inferred valuation methods to evaluate the conservation of olive groves and associated ecosystem services in Andalusia (Spain). Ecosyst Serv. 2017;26:258-69. doi: 10.1016/j.ecoser.2017.07.002.

76.	Zheng Y, Takahashi T, Kemp D. Energy and economic values of greenhouse sheds to replace winter grazing in northwestern China. Grassl Sci. 2013;59(3):156-9. doi: : 10.1111/grs.12027. PubMed PMID: CCC:000323848200004.

77.	Bai Y, Ouyang ZY, Zheng H, Xu WH, Jiang B, Fang Y. Environmental benefit-loss analysis of agro-ecosvstem in Haihe River basin, China. Chin J Appl Ecol. 2010;21(11):2938-45. PubMed Central PMCID: PMC21361021.

78.	Al-Saedi TK, Sayed K, Syakir MI, Umar Y, Azhar B, Tohiran KA, et al. Enhancing soil quality and economic value in oil palm plantations: Effects of livestock integration on earthworm abundance and ecosystem services. J Nat Conserv. 2025;86:126939-52. doi: 10.1016/j.jnc.2025.126939.

79.	Huang C-H, Wang C-H. Estimating the Total Economic Value of Cultivated Flower Land in Taiwan. Sustainability. 2015;7(4):4764-82. doi: 10.3390/su7044764. PubMed PMID: CCC:000353715400066.

80.	Shiferaw H, Alamirew T, Kassawmar T, Zeleke G. Evaluating ecosystems services values due to land use transformation in the Gojeb watershed, Southwest Ethiopia. Environ Syst Res. 2021;10(1):1-12. doi: 10.1186/s40068-021-00227-3.

81.	Zhang H-F, Ouyang Z-Y, Zheng H, Xiao Y. Evaluation of agricultural ecosystem services value in Manas River Watershed of China. Chin J Eco-Agric. 2009;17(6):1259-64. doi: 10.3724/sp.j.1011.2009.01259.

82.	Madiwalar AF, Parthiban KT. Evaluation of Cultural Ecosystem Services of Pulpwood Multifunctional Agroforestry: A Case Study from the Foothills of the Nilgiris, Western Ghats, India. Appl Ecol Environ Res. 2023;21(3):2611-24. doi: 10.15666/aeer/2103_26112624.

83.	Feketéné FA, Szűcs I, Bauerné Gáthy A. Evaluation of the Pollination Ecosystem Service of the Honey Bee (*Apis mellifera*) Based on a Beekeeping Model in Hungary. Sustainability. 2023;15(13):9906-19. doi: 10.3390/su15139906. PubMed PMID: doi:10.3390/su15139906.

84.	Yang X, Burton M, Cai Y, Zhang A. Exploring Heterogeneous Preference for Farmland Non-market Values in Wuhan, Central China. Sustainability. 2016;8(1):12-24. doi: 10.3390/su8010012.

85.	Lopes LFG, Bento JMRdS, Cristovão AFAC, Baptista FO. Exploring the effect of land use on ecosystem services: the distributive issues. Land Use Policy. 2017;45:141–9. doi: 10.1016/j.landusepol.2014.12.008.

86.	Chang K, Ying YH. External benefits of preserving agricultural land: Taiwan's rice fields. Soc Sci J. 2005;42(2):285-93. doi: 10.1016/j.soscij.2005.03.008. PubMed PMID: CCC:000229898200009.

87.	Priess JA, Mimler M, Klein A-M, Schwarze S, Tscharntke T, Steffan-Dewenter I. Linking deforestation scenarios to pollination services and economic returns in coffee agroforestry systems. Ecol Appl. 2007;17(2):407–17. doi: 10.3410/f.1087776.540693.

88.	Azzi M, Calatrava J, Bedrani S. Farmers' willingness to pay for surface water in the West Mitidja irrigated perimeter, northern Algeria. Span J Agric Res. 2018;16(1):101-16. doi: 10.5424/sjar/201816112073. PubMed PMID: CCC:000431124100018.

89.	Tao J, Wang J. Farmers’ willingness to accept compensation for livestock and poultry waste resource utilization and its determinants. Chin J Popul Resour Environ. 2020;18(2):144-54. doi: 10.1016/j.cjpre.2021.04.019.

90.	Bani BK, Damnyag L. Farmers’ Willingness to Pay for the Provision of Ecosystem Services to Enhance Agricultural Production in Sene East District, Ghana. Small-scale For. 2017;16(4):451-67. doi: 10.1007/s11842-017-9364-3.

91.	Flugge F, Abadi A. Farming carbon : an economic analysis of agroforestry for carbon sequestration and dryland salinity reduction in Western Australia. Agrofor Syst. 2006;68(3):181-92. doi: 10.1007/s10457-006-9008-7.

92.	Liu H, Hou L, Nan Z, Huang J, Su L. Herders’ willingness to accept compensation for grassland grazing ban in Northwest China. J Integr Agric. 2025;24(1):366-79. doi: 10.1016/j.jia.2024.11.036.

93.	Skordos D, Ragkos A, Karanikolas P, Vlahos G. How valuable are the ecosystem services of local animal breeds? The case of Kalaritiko sheep in Greece. Livest Sci. 2024;279:105391-9. doi: 10.1016/j.livsci.2023.105391.

94.	Biratu AA, Bedadi B, Gebreyohannis SG, Melesse AM, Nebi TH, Abera W, et al. Impact of landscape management scenarios on ecosystem service values in Central Ethiopia. Land. 2022;11:1266-81. doi: 10.3390/land11081266.

95.	Hardaker A, Pagella T, Rayment M. Integrated assessment, valuation and mapping of ecosystem services and dis-services from upland land use in Wales. Ecosyst Serv. 2020;43:101098-128. doi: 10.1016/j.ecoser.2020.101098.

96.	Herd-Hoare SC, Shackleton CM. Integrating Ecosystem Services and Disservices in Valuing Smallholder Livestock and Poultry Production in Three Villages in South Africa. Land. 2020;9(9):294-313. doi: 10.3390/land9090294.

97.	Pradhan G, Meena RS. Interaction impact of biocompost on nutrient dynamics and relations with soil biota, carbon fractions index, societal value of CO_2_ equivalent and ecosystem services in the wheat-rice farming. Chemosphere. 2023;339:139695-706. doi: 10.1016/j.chemosphere.2023.139695. PubMed PMID: MEDLINE:37536540.

98.	Cahyandito MF, Ramadhan R. Is converting shrub land to palm oil plantation worth it? - An ecosystem services analysis. J Bus Retail Manag Res. 2015;10(1):77-86.

99.	Guo T, Wu S, Zhang X, Zhang C, Yang J, Cheng S. Measurement and Influencing Factors of Willingness to Accept Payment for Ecosystem Service Provision: A Case Study of a Leading Forest Farm in China. Forests. 2023;14(12):2417-32. doi: 10.3390/f14122417. PubMed PMID: CCC:001131289600001.

100.	Mwebaze P, Marris GC, Brown M, MacLeod A, Jones G, Budge GE. Measuring public perception and preferences for ecosystem services: A case study of bee pollination in the UK. Land Use Policy. 2018;71:355-62. doi: 10.1016/j.landusepol.2017.11.045. PubMed PMID: CCC:000423643400033.

101.	Cai YY, Zhang AL. Measuring recreational value and existence value of agricultural land in Shiliuhong leisure farm in Wuhan. Acta Ecol Sin. 2008;28(3):1201-10. doi: 10.0000/j.1000-0933.2008280312011210.

102.	Hosseini-Yekani S-A, Tomaczewski S, Zander P. Model-Based Valuation of Ecosystem Services Using Bio-Economic Farm Models: Insights for Designing Green Tax Policies and Payment for Ecosystem Services. Agriculture. 2024;15(1):60-. doi: 10.3390/agriculture15010060.

103.	Korneeva EA. Monetary Valuation of Ecosystem Services Provided by Protective Forest Plantations in the Agroforestry System in the South of the Volga Upland. Forests. 2023;14(10):1955-71. doi: 10.3390/f14101955.

104.	Admasu WF, Van Passel S, Minale AS, Tsegaye EA, Nyssen J. Nexus between land development and the value of ecosystem services in Ethiopia: A contingent valuation study. Environ Dev Sustain. 2024;26(2):3193-213. doi: 10.1007/s10668-022-02803-8. PubMed PMID: CCC:001159754600039.

105.	Picanço A, Gil AJF, Rigal F, Borges PAV. Pollination services mapping and economic valuation from insect communities : a case study in the Azores (Terceira Island). Nat Conserv. 2017;18:1-25. doi: 10.3897/natureconservation.18.11523.

106.	Lehmann LM, Smith J, Westaway S, Pisanelli A, Russo G, Borek R, et al. Productivity and Economic Evaluation of Agroforestry Systems for Sustainable Production of Food and Non-Food Products. Sustainability. 2020;12(13):5429-37. doi: 10.3390/su12135429.

107.	Sandoval D, Florez F, Enciso K, Sotelo M, Burkart S, editors. Profitability analysis of a silvo-pastoral system in Colombia: Economic and environmental benefits2023: International Center for Tropical Agriculture.

108.	Ghaley BB, Vesterdal L, Porter JR. Quantification and valuation of ecosystem services in diverse production systems for informed decision-making. Environ Sci Policy. 2014;39:139-49. doi: 10.1016/j.envsci.2013.08.004. PubMed PMID: CCC:000336948800013.

109.	Oddabasawa, Chittapur B. Quantification of ecoservices in traditional agroforestry systems in semi arid tropics. Bangladesh J Bot. 2021;50(2):427-32. doi: 10.3329/bjb.v50i2.54102.

110.	Ando Fahda A, Sandhu H, Millington AC. Quantifying the Economic Value of Ecosystem Services in Oil Palm Dominated Landscapes in Riau Province in Sumatra, Indonesia. Land. 2020;9:194-215. doi: 10.3390/land9060194.

111.	Bernués A, Rodríguez-Ortega T, Alfnes F, Clemetsen M, Eik LO. Quantifying the multifunctionality of fjord and mountain agriculture by means of sociocultural and economic valuation of ecosystem services. Land Use Policy. 2015;48:170-8. doi: 10.1016/j.landusepol.2015.05.022.

112.	Ratto F, Breeze TD, Cole LJ, Garratt MPD, Kleijn D, Kunin B, et al. Rapid assessment of insect pollination services to inform decision-making. Conserv Biol. 2022;36(4):13886-98. doi: 10.1111/cobi.13886. PubMed PMID: CCC:000831343200018.

113.	Sandhu H, Waterhouse B, Boyer S, Wratten S. Scarcity of ecosystem services: an experimental manipulation of declining pollination rates and its economic consequences for agriculture. PeerJ. 2016;4:2099-20115. doi: 10.7717/peerj.2099.

114.	Connor JD, Summers D, Regan C, Abbott H, Linden LVD, Frizenschaf J. Sensitivity analysis in economic evaluation of payments for water and carbon ecosystem services. Ecosyst Serv. 2022;54:101416-28. doi: 10.1016/j.ecoser.2022.101416.

115.	Sandhu H, Wratten S, Costanza R, Pretty J, Porter JR, Reganold J. Significance and value of non-traded ecosystem services on farmland. PeerJ. 2015:1-22. doi: 10.7717/peerj.762.

116.	Letourneau DK, Ando A, Jedlicka JA, Narwani A, Barbier E. Simple-but-sound methods for estimating the value of changes in biodiversity for biological pest control in agriculture. Ecol Econ. 2015;120:215-25. doi: 10.1016/j.ecolecon.2015.10.015.

117.	Chia SY, Macharia J, Diiro GM, Kassie M, Ekesi S, van Loon JJA, et al. Smallholder farmers' knowledge and willingness to pay for insect-based feeds in Kenya. PLOS ONE. 2020;15(3):e0230552. doi: 10.1371/journal.pone.0230552. PubMed Central PMCID: PMC32210461.

118.	Wassihun AN, Nega YM, Kebede WM, Fenta EE, Ayalew AA. Smallholder households’ willingness to pay for sustainable agricultural water supply in case of North West Ethiopia. Lett Spat Resour Sci. 2022;15(1):79-98. doi: 10.1007/s12076-022-00300-0.

119.	Bernués A, Rodríguez-Ortega T, Ripoll-Bosch R, Alfnes F. Socio-Cultural and Economic Valuation of Ecosystem Services Provided by Mediterranean Mountain Agroecosystems. PLOS ONE. 2014;9(7):1-11. doi: 10.1371/journal.pone.0102479.

120.	Faccioni G, Sturaro E, Ramanzin M, Bernués A. Socio-economic valuation of abandonment and intensification of Alpine agroecosystems and associated ecosystem services. Land Use Policy. 2019;81:453–62. doi: 10.1016/j.landusepol.2018.10.044.

121.	Liu M-C, Lun F, Zhang C-Q, Li W-H. Standards of payments for paddy ecosystem services: using Hani Terrace as case study. Chin J Eco-Agric. 2012;20(6):703-9. doi: 10.3724/sp.j.1011.2012.00703.

122.	Sabino W, Costa L, Andrade T, Teixeira J, Araújo G, Acosta AL, et al. Status and trends of pollination services in Amazon agroforestry systems. Agriculture, Ecosystems and Environment. 2022;335:108012-23. doi: 10.1016/j.agee.2022.108012.

123.	Jin J, Jiang C, Li L. The economic valuation of cultivated land protection: A contingent valuation study in Wen ling City, China. Landsc Urban Plan. 2013;119:158-64. doi: 10.1016/j.landurbplan.2013.06.010. PubMed PMID: CCC:000325196300017.

124.	Azahara Mesa-Jurado M, Martin-Ortega J, Ruto E, Berbel J. The economic value of guaranteed water supply for irrigation under scarcity conditions. Agric Water Manag. 2012;113:10-8. PubMed PMID: CCC:000308384800002.

125.	Feuerbacher A, Herbold T, Krumbe F. The Economic Value of Pollination Services for Seed Production: A Blind Spot Deserving Attention. Environ Resour Econ. 2024;87:881–905. doi: 10.1007/s10640-024-00840-7.

126.	Vrabcová P, Hájek M. The Economic Value of the Ecosystem Services of Beekeeping in the Czech Republic. Sustainability. 2020;12(23):10179-89. doi: 10.3390/su122310179.

127.	Perrot T, Gaba S, Roncoroni M, Gautier J-L, Bretagnolle V. Bees increase oilseed rape yield under real field conditions. Agriculture, Ecosystems & Environment. 2018;266:39-48. doi: 10.1016/j.agee.2018.07.020.

128.	Sujetoviene G, Dabasinskas G. The impact of restoration of Lithuanian peatlands on the economic value of certain ecosystem services. Ecol Eng. 2025;212:107475-83. doi: 10.1016/j.ecoleng.2024.107475. PubMed PMID: CCC:001407602300001.

129.	Alcon F, Pedrero F, Martin-Ortega J, Arcas N, Alarcon JJ, de Miguel MD. The non-market value of reclaimed wastewater for use in agriculture: a contingent valuation approach. Span J Agric Res. 2010;8:187-96. PubMed PMID: CCC:000284660300018.

130.	Ning J, Jin J, Kuang F, Wan X, Zhang C, Guan T. The Valuation of Grassland Ecosystem Services in Inner Mongolia of China and Its Spatial Differences. Sustainability. 2019;11(24):7117-31. doi: 10.3390/su11247117. PubMed PMID: CCC:000506899000220.

131.	Borges RC, de Brito RM, Imperatriz-Fonseca VL, Giannini TC. The Value of Crop Production and Pollination Services in the Eastern Amazon. Neotrop Entomol. 2020;49(4):545-56. doi: 10.1007/s13744-020-00791-w.

132.	Porter J, Costanza R, Sandhu H, Sigsgaard L, Wratten S. The value of producing food, energy, and ecosystem services within an agro-ecosystem. Ambio 2009;38(4):186-93.

133.	Mazzocchi C, Sali G. Tourists’ Perception of Ecosystem Services Provided by Mountain Agriculture. Sustainability. 2022;14(19):1-14. doi: 10.3390/su141912171.

134.	Mousavi SA, Mohammadi Kharaji A, Soltani S. Tradeoff between production and regulation functions of semi-arid rangelands. Rangel J. 2023;45(1):36-44. doi: 10.1071/RJ22065.

135.	Cao JJ, Ren ZW, Yang Y, Du GZ. The Combined Application of WTP and WTA in Contingent Valuation Methods. J Environ Prot. 2008;1:284-92.

136.	Mekonnen A, Gebreegziabher Z, Beyene AD, Hagos F, editors. Valuation of access to irrigation water in rural Ethiopia: Application of choice experiment and contingent valuation methods. Ageconsearch; 2018.

137.	Garcia JNM, Aguilar EA, Sangalang JB, Alcantara AJ, Habito RCF, Medina CP, et al. Valuation of ecosystem services of coconut types: framework and methodology development. J Environ Sci Manag. 2009;12(1):68-78.

138.	Barrena J, Nahuelhual L, Báez A, Schiappacasse I, Cerda C. Valuing cultural ecosystem services: Agricultural heritage in Chiloé island, southern Chile. Ecosyst Serv. 2014;7:66-75. doi: 10.1016/j.ecoser.2013.12.005.

139.	Thomasz EO, Kasanzew A, Massot JM, Garcia-Garcia A. Valuing ecosystem services in agricultural production in southwest Spain. Ecosyst Serv. 2024;68:101636-7651. doi: 10.1016/j.ecoser.2024.101636. PubMed PMID: CCC:001247069000001.

140.	Baniasadi M, Eydipour M. Valuing ecosystem services of community gardens in developing countries: a case study of Dezful City in Iran. Nat Conserv. 2024;56:127-49. doi: 10.3897/natureconservation.56.127283. PubMed PMID: CCC:001338490800001.

141.	Karthick V, Ganesh M, Sangeetha R. Valuing ecosystem services of Melia dubia tree plantation in farm lands. Range Manag Agrofor. 2023;44(1):102 -7. doi: 10.59515/rma.2023.v44.i1.12.

142.	Liu S, Costanza R, Troy A, D'Aagostino J, Mates W. Valuing New Jersey's ecosystem services and natural capital: A spatially explicit benefit transfer approach. Environ Manage. 2010;45(6):1271-85. doi: 10.1007/s00267-010-9483-5. PubMed Central PMCID: PMC20396885.

143.	Temesgen H, Wu W, Shi X, Yirsaw E, Bekele B, Kindu M. Variation in Ecosystem Service Values in an Agroforestry Dominated Landscape in Ethiopia: Implications for Land Use and Conservation Policy. Sustainability. 2018;10(4):1-20. doi: 10.3390/su10041126.

144.	Vivithkeyoonvong S, Jourdain D. Willingness to pay for ecosystem services provided by irrigated agriculture in Northeast Thailand. Int J Biodivers Sci Ecosyst Serv Manag. 2016;13(1):14-26. doi: 10.1080/21513732.2016.1242093.

145.	My NHD, Kien ND, Hung PX, Anh LTQ. Does the Value of Water-Related Ecosystem Services Capture Water Scarcity? Application to Rice Farming in the Mekong Delta of Vietnam? Water Econ Policy. 2024;10(01):131-67. doi: 10.1142/S2382624X23500066. PubMed PMID: CCC:001225963900003.

146.	Huguenin MT, Leggett CG, Paterson RW. Economic valuation of soil fauna. Eur J Soil Biol. 2006;42:S16-S22. doi: 10.1016/j.ejsobi.2006.10.003. PubMed PMID: CCC:000243263800003.
