# Supplementary material for: Economic valuation of ecosystem services in agriculture and agroforestry: A meta-analysis
Source: PLoS One. 2026 Oct 16;21(10):e0360792. doi: 10.1371/journal.pone.0360792 (PMC13674745; doi:10.1371/journal.pone.0360792)
Supplement: Supplementary file 4 [file pone.0360792.s024.docx]

# 1. Report of metaregression (USD.month excluded with severe VIF)

## 1.1. 1.Multicollinearity - VIF

| Variable | VIF | Interpretation |
| --- | --- | --- |
| PROV | 7.605233 | Modere (5-10) |
| REG | 5.239858 | Modere (5-10) |
| CULT | 4.123481 | OK (<5) |
| SUP | 2.344482 | OK (<5) |
| MPV | 7.196733 | Modere (5-10) |
| CVM | 5.053365 | Modere (5-10) |
| BTM | 2.801648 | OK (<5) |
| CE | 2.880764 | OK (<5) |
| ACM | 2.095872 | OK (<5) |
| TEV | 1.726926 | OK (<5) |
| CBA | 1.858341 | OK (<5) |
| BEA | 3.299448 | OK (<5) |
| RCA | 2.788972 | OK (<5) |
| LCIA | 6.148777 | Modere (5-10) |
| DBDC | 1.693079 | OK (<5) |
| TCM | 1.688366 | OK (<5) |
| USD.ha | 5.931127 | Modere (5-10) |
| USD | 2.400081 | OK (<5) |
| USD.year | 3.200642 | OK (<5) |
| USD.ha.year | 4.219380 | OK (<5) |
| USD.person.year | 2.035959 | OK (<5) |
| AF | 1.852316 | OK (<5) |
| CAF | 1.932614 | OK (<5) |
| CP | 2.885023 | OK (<5) |
| CF | 2.887863 | OK (<5) |
| CG | 3.131784 | OK (<5) |

## 1.2. 2. Complete model (z = Wald z ; IC à 95 %)

Thresholds: *p<0.1 **p<0.05 ***p<0.01

| term | estimate | std.error | z_value | conf.low | conf.high | p.value | Stars | Group |
| --- | --- | --- | --- | --- | --- | --- | --- | --- |
| intercept | 3.819290225 | 0.7175509 | 5.32267478 | 2.41291625 | 5.2256642 | 0.0000001022525 | *** | Other |
| PROV | -0.395608340 | 0.3720839 | -1.06322342 | -1.12487943 | 0.3336627 | 0.2876806450595 |  | Ecosystem |
| REG | -0.853500461 | 0.5543287 | -1.53970109 | -1.93996470 | 0.2329638 | 0.1236332320465 |  | Ecosystem |
| CULT | -1.569819693 | 0.7380375 | -2.12701902 | -3.01634652 | -0.1232929 | 0.0334185031354 | ** | Ecosystem |
| SUP | -0.053555277 | 1.2087030 | -0.04430805 | -2.42256956 | 2.3154590 | 0.9646588516116 |  | Ecosystem |
| MPV | 0.860248503 | 0.4727879 | 1.81952318 | -0.06639871 | 1.7868957 | 0.0688316492832 | * | Valuation |
| CVM | -0.016466063 | 0.3956635 | -0.04161632 | -0.79195236 | 0.7590202 | 0.9668045595589 |  | Valuation |
| BTM | 1.192621247 | 0.6128741 | 1.94594819 | -0.00858992 | 2.3938324 | 0.0516609639708 | * | Valuation |
| CE | -0.007950554 | 0.4380585 | -0.01814952 | -0.86652947 | 0.8506284 | 0.9855195699236 |  | Valuation |
| ACM | 1.903054936 | 0.9801165 | 1.94166197 | -0.01793811 | 3.8240480 | 0.0521780360492 | * | Valuation |
| TEV | 0.894552400 | 0.8698501 | 1.02839835 | -0.81032246 | 2.5994273 | 0.3037624863395 |  | Valuation |
| CBA | 1.317220851 | 0.8994715 | 1.46443862 | -0.44571096 | 3.0801527 | 0.1430741426017 |  | Valuation |
| BEA | -2.274207319 | 1.4695325 | -1.54757198 | -5.15443809 | 0.6060235 | 0.1217253817638 |  | Valuation |
| RCA | 0.508884038 | 1.0875048 | 0.46793727 | -1.62258623 | 2.6403543 | 0.6398294470937 |  | Valuation |
| LCIA | 2.941634404 | 1.4577081 | 2.01798596 | 0.08457910 | 5.7986897 | 0.0435927238997 | ** | Valuation |
| DBDC | 1.261956314 | 0.6187780 | 2.03943318 | 0.04917379 | 2.4747388 | 0.0414068138269 | ** | Valuation |
| TCM | 1.118743669 | 0.8142574 | 1.37394354 | -0.47717147 | 2.7146588 | 0.1694592111919 |  | Valuation |
| USD.ha | 0.046778832 | 0.4590709 | 0.10189894 | -0.85298352 | 0.9465412 | 0.9188368945825 |  | Conversion |
| USD | 1.051842554 | 0.4078694 | 2.57887072 | 0.25243314 | 1.8512520 | 0.0099123875052 | *** | Conversion |
| USD.year | 0.527563415 | 0.4473500 | 1.17930802 | -0.34922643 | 1.4043533 | 0.2382755473753 |  | Conversion |
| USD.ha.year | -1.625990388 | 0.6485322 | -2.50718531 | -2.89709013 | -0.3548906 | 0.0121696893069 | ** | Conversion |
| USD.person.year | 0.530390450 | 0.5014471 | 1.05771973 | -0.45242773 | 1.5132086 | 0.2901832377220 |  | Conversion |
| AF | 1.175402485 | 1.1800882 | 0.99602935 | -1.13752789 | 3.4883329 | 0.3192358839774 |  | Agricultural |
| CAF | -0.316730473 | 1.5342642 | -0.20643803 | -3.32383299 | 2.6903720 | 0.8364487672415 |  | Agricultural |
| CP | -0.369763825 | 1.2395152 | -0.29831325 | -2.79916905 | 2.0596414 | 0.7654640885997 |  | Agricultural |
| CF | 2.597628385 | 1.5835573 | 1.64037535 | -0.50608691 | 5.7013437 | 0.1009271464175 |  | Agricultural |
| CG | 0.896791581 | 1.6438262 | 0.54555135 | -2.32504848 | 4.1186316 | 0.5853743730437 |  | Agricultural |

## 1.3. 3. Selection of model — 3 models comparison

| Model | k | QM | QM_pval | QE | QE_pval | tau^2^ | I^2^ | logLik | AIC | BIC | pseudoR^2^ |
| --- | --- | --- | --- | --- | --- | --- | --- | --- | --- | --- | --- |
| Complet | 61 | 85.36568947 | 3.04836E-08 | 456808.3636 | 0 | 2.957925643 | 99.98405886 | -121.6576108 | 299.3152216 | 358.4196898 | 59.57802524 |
| Reduced Significants | 61 | 37.01740998 | 1.14244E-05 | 1847792.207 | 0 | 4.501763224 | 99.9945464 | -133.4453039 | 286.8906078 | 307.9993464 | 38.48048214 |
| Non-significant reductions | 61 | 25.12895106 | 0.121405222 | 1335150.471 | 0 | 5.106401824 | 99.99372096 | -137.4500929 | 314.9001858 | 357.1176631 | 30.21770303 |

### 1.3.1. VIF of submodels

| Model | Variable | VIF |
| --- | --- | --- |
| Complet | PROV | 7.605233 |
| Complet | REG | 5.239858 |
| Complet | CULT | 4.123481 |
| Complet | SUP | 2.344482 |
| Complet | MPV | 7.196733 |
| Complet | CVM | 5.053365 |
| Complet | BTM | 2.801648 |
| Complet | CE | 2.880764 |
| Complet | ACM | 2.095872 |
| Complet | TEV | 1.726926 |
| Complet | CBA | 1.858341 |
| Complet | BEA | 3.299448 |
| Complet | RCA | 2.788972 |
| Complet | LCIA | 6.148777 |
| Complet | DBDC | 1.693079 |
| Complet | TCM | 1.688366 |
| Complet | USD.ha | 5.931127 |
| Complet | USD | 2.400081 |
| Complet | USD.year | 3.200642 |
| Complet | USD.ha.year | 4.219380 |
| Complet | USD.person.year | 2.035959 |
| Complet | AF | 1.852316 |
| Complet | CAF | 1.932614 |
| Complet | CP | 2.885023 |
| Complet | CF | 2.887863 |
| Complet | CG | 3.131784 |
| Reduced Significants | CULT | 1.454905 |
| Reduced Significants | MPV | 1.963299 |
| Reduced Significants | BTM | 1.087948 |
| Reduced Significants | ACM | 1.453874 |
| Reduced Significants | LCIA | 1.224406 |
| Reduced Significants | DBDC | 1.111917 |
| Reduced Significants | USD | 1.240896 |
| Reduced Significants | USD.ha.year | 1.881897 |
| Non-significant reductions | PROV | 3.468632 |
| Non-significant reductions | REG | 2.170114 |
| Non-significant reductions | SUP | 1.513671 |
| Non-significant reductions | CVM | 2.863534 |
| Non-significant reductions | CE | 1.827868 |
| Non-significant reductions | TEV | 1.495956 |
| Non-significant reductions | CBA | 1.201317 |
| Non-significant reductions | BEA | 1.757960 |
| Non-significant reductions | RCA | 1.918486 |
| Non-significant reductions | TCM | 1.100429 |
| Non-significant reductions | USD/ha | 3.558199 |
| Non-significant reductions | USD/year | 1.839747 |
| Non-significant reductions | USD/person/year | 1.764818 |
| Non-significant reductions | AF | 1.157191 |
| Non-significant reductions | CAF | 1.426902 |
| Non-significant reductions | CP | 1.924255 |
| Non-significant reductions | CF | 2.107619 |
| Non-significant reductions | CG | 2.268083 |

## 1.4. 4. Publication biases - Egger test

| z_Egger | p_Egger | interpretation |
| --- | --- | --- |
| -0.6584586 | 0.5102435 | No significant asymmetry |
